# Weighted Gene Correlation Network Analysis Identifies Specific Functional Modules and Genes in Esophageal Cancer

**DOI:** 10.1155/2021/8223263

**Published:** 2021-12-27

**Authors:** Wei Xu, Jian Xu, Zhiqiang Wang, Yuequan Jiang

**Affiliations:** Chongqing Key Laboratory of Translational Research for Cancer Metastasis and Individualized Treatment, Chongqing University Cancer Hospital, Chongqing 400030, China

## Abstract

**Objective:**

Esophageal cancer (ESCA) is one of the most aggressive malignancies globally with an undesirable five-year survival rate. Here, this study was conducted for determining specific functional genes linked with ESCA initiation and progression.

**Methods:**

Gene expression profiling of ESCA was curated from TCGA (containing 160 ESCA and 11 nontumor specimens) and GSE38129 (30 paired ESCA and nontumor tissues) datasets. Differential expression analysis was conducted between ESCA and nontumor tissues with adjusted *p* value <0.05 and |log2fold-change|>1. Weighted gene coexpression network analysis (WGCNA) was conducted for determining the ESCA-specific coexpression modules and genes. Thereafter, ESCA-specific differentially expressed genes (DEGs) were intersected. Functional enrichment analysis was then presented with clusterProfiler package. Protein-protein interaction was conducted, and hub genes were determined. Association of hub genes with pathological staging was evaluated, and survival analysis was presented among ESCA patients.

**Results:**

This study determined 91 ESCA-specific DEGs following intersection of DEGs and ESCA-specific genes in TCGA and GSE38129 datasets. They were remarkably linked to cell cycle progression and carcinogenic pathways like the p53 signaling pathway, cellular senescence, and apoptosis. Ten ESCA-specific hub genes were determined, containing *ASPM*, *BUB*1*B*, *CCNA*2, *CDC*20, *CDK*1, *DLGAP*5, *KIF*11, *KIF*20* A*, *TOP*2*A*, and *TPX*2. They were prominently associated with pathological staging. Among them, KIF11 upregulation was in relation to undesirable prognosis of ESCA patients.

**Conclusion:**

Collectively, we determined ESCA-specific coexpression modules and hub genes, which offered the foundation for future research concerning the mechanistic basis of ESCA.

## 1. Introduction

Esophageal cancer (ESCA) ranks the eighth major cancer type as well as the sixth major cause of cancer-relevant deaths across the globe [1]. Tobacco and alcohol consumption are the main environmental risk factors of ESCA. The five-year survival rate is nearly 15% [2]. It mainly contains two histological subtypes: esophageal squamous cell carcinoma (approximately 90%) and esophageal adenocarcinoma (around 10%) [3]. Patients' advanced clinical presentation is linked to locally late and distant metastasis, which contributes to undesirable survival outcome. Additionally, because of tumor heterogeneity and acquired drug resistance, inherent resistance to radiotherapy and chemotherapy triggers therapeutic failure and unfavorable survival rate [2]. ESCA therapy depends upon patients' and tumors' features, especially the tumor, node, metastasis (TNM) staging system [4]. In the early stage, patients are suitable for endoscopic resection, while those in the advanced stage receive surgical resection, chemotherapy, chemoradiotherapy, or their combination [4]. For patients with unresectable ESCA, systemic chemotherapy is applied. Additionally, immunotherapy has emerged as a therapeutic option for advanced or metastatic patients [5]. Although the therapeutic options have been steadily increasing, the molecular mechanisms underlying ESCA remain indistinct.

The pathogenesis of ESCA is a multistep process, involving distinct stages until eventually cancers [6]. Hence, to focus on the molecular mechanisms underlying the initiation and progression of ESCA may assist uncover underlying diagnostic markers or treatment targets. Weighted gene coexpression network analysis (WGCNA) is a reliable systematic biological algorithm, which may emphasize coexpression genomic modules and effectively evaluate the interactions between coexpression modules and clinical phenotypes [7]. This algorithm has been widely utilized for discovering cancer-specific modules and hub genes like bladder cancer [7], hepatocellular carcinoma [8], and lung cancer [9]. Limited studies have applied the WGCNA method to uncover the pathogenesis of ESCA. For instance, Nangraj et al. identified hub genes shared between Barrett's esophagus and esophageal adenocarcinoma through integrated analysis of protein-protein interaction (PPI) and WGCNA [10]. Through WGCNA, *miR-*92*b-*3*p* was determined as a pathogenic gene in ESCA [11]. Integrated analysis of WGCNA and network pharmacology deciphered the molecular mechanisms of compound Kushen injection in ESCA treatment [12]. Here, this study adopted the WGCNA algorithm for determining specific functional modules and genes in ESCA, offering the foundation for future research concerning the mechanistic basis of ESCA.

## 2. Materials and Methods

### 2.1. Data Collection and Preprocessing

The RNA-seq data of ESCA were retrieved from the Cancer Genome Atlas (TCGA) GDC Application Programming Interface. Gene expression profiling data (read counts) were processed and transformed into gene ID Ensembl (version 90). In total, 160 ESCA and 11 normal tissues were included. Microarray expression profiling of 30 ESCC tumors and adjacent normal tissues was curated from the GSE38129 dataset [13] in the Gene Expression Omnibus (GEO; https://www.ncbi.nlm.nih.gov/gds/) repository. This dataset was in accordance with the GPL571 platform ((HG-U133 A_2) Affymetrix Human Genome U133A 2.0 Array). The raw expression profiling was background-corrected and normalized by quantile utilizing the robust multiarray average (RMA) method.

### 2.2. Differential Expression Analysis

Differentially expressed genes (DEGs) were selected utilizing the linear models for microarray data (limma; version 3.50.0) package through comparison of the expression profiling between ESCA and normal tissues [14]. The matched *p* values of gene symbols following the *t-*test were calculated, and adjusted *p* value <0.05 and |log2fold-change|>1 were set as the selection criteria. The volcano and heatmap of the DEGs were drawn.

### 2.3. WGCNA

Coexpression networks were separately established in TCGA and GSE38129 datasets utilizing WGCNA package (version 1.69) [15]. The genes with the first 25% standard deviation were chosen as the input genes. For constructing a scale-free network, the optimal soft threshold power value (*β*; ranging from 1 to 20) was determined with the “pickSoftThreshold” function through calculation of the scale-free fit index. Pearson's correlation matrix was conducted for evaluating the similarity among the pairwise genes utilizing the “cor” function. Thereafter, the adjacency was determined in accordance with *β* and Pearson's correlation matrix utilizing the “TOMsimilarity” function. Meanwhile, the corresponding dissimilarity (dissTOM) was determined. The modules were segmented with a dynamic cut tree algorithm, and similar modules were merged into one. Module eigengenes (MEs) that were the first principal component of gene expression patterns within a specific module were identified for each module.

### 2.4. Identification of ESCA-Relevant Coexpression Models

In this study, the most crucial critical feature was tissue type that was designated as ESCA tumor and normal specimens. Pearson correlation between MEs and clinical feature was analyzed. Modules that possessed the strongest correlation coefficient were determined as the ESCA-relevant coexpression models. Module membership indicates the intramodule connectivity of any gene within a given module. The higher the absolute value of module membership, the higher the negative or positive correlation between the gene with the module eigengenes. Gene significance was utilized for incorporating external information to the coexpression network. The higher the absolute value of gene significance, the higher the biological significance of a gene for tissue type. ESCA-relevant genes within the ESCA-relevant coexpression models were determined in accordance with module membership >0.8 and gene significance >0.5.

### 2.5. Identification of ESCA-Specific DEGs

For achieving the intersection of DEGs and coexpressed genes, an online web tool (http://bioinfogp.cnb.csic.es/tools/venny/index.html) was adopted for plotting Venn diagram.

### 2.6. Function Enrichment Analysis

Functional annotation of ESCA-specific DEGs was presented with the clusterProfiler package (version 4.2.0), containing Gene Ontology (GO) and Kyoto Encyclopedia of Genes and Genomes (KEGG) pathway analysis [16]. GO terms comprised of the biological process (BP), cellular component (CC), and molecular function (MF).

### 2.7. Protein-Protein Interaction (PPI) Analysis

The PPI network of ESCA-specific DEGs was conducted on the basis of the Search Tool for the Retrieval of Interacting Genes/Proteins (STRING; version 11.0; https://string-db.org) online tool [17]. The CytoHubba plugin [18] of Cytoscape software (version 3.7.2) [19] was adopted for selecting the hub genes within the PPI network [18]. Herein, the first 10 genes were determined as hub genes.

### 2.8. Survival Analysis

In accordance with the optimal cutoff value determined by survival package, ESCA patients were stratified into high and low expression groups of the 10 ESCA-specific hub genes. Kaplan–Meier curves of overall survival were conducted between groups, and log-rank tests were utilized for comparing the survival differences.

### 2.9. Statistical Analysis

All the analyses in this study were implemented utilizing *R* software (version 3.5.1). Student's *t* test or Wilcoxon test was adopted for comparisons between groups. Spearman correlation analysis was carried out to evaluate the interactions of the 10 ESCA-specific hub genes with pathological staging of ESCA patients. *P* value<0.05 indicated the statistical significance.

## 3. Results

### 3.1. Exploration of DEGs in ESCA

**Figure 1:**
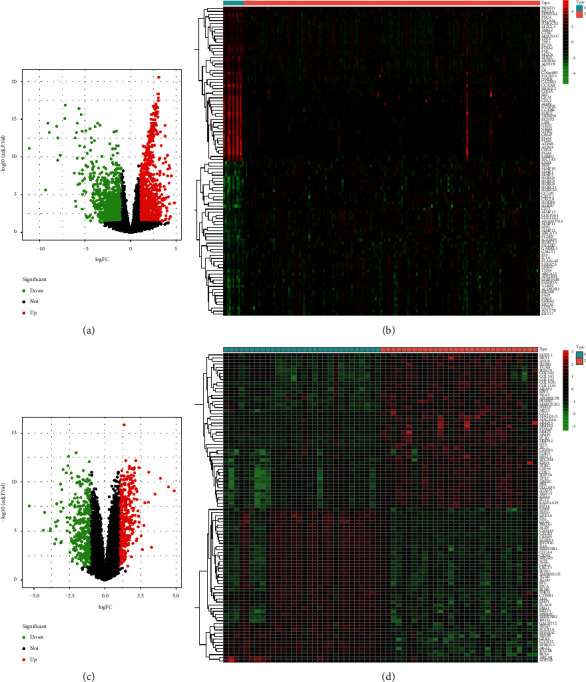
Analysis of DEGs of ESCA both in TCGA and GSE38129 datasets. (a) Volcano plots depict the results of differential expression analysis between 160 ESCA tumors and 11 normal tissues in TCGA cohort. Red bubble indicates upregulated gene in ESCA; green bubble represents downregulated gene in ESCA; black bubble is indicative of nonsignificant gene. (b) Heatmap visualizes the expression patterns of DEGs with adjusted *p* value <0.05 and |log2fold-change|>1 in 160 ESCA tumors (T) and 11 normal tissues (N) in TCGA cohort. Red represents upregulation, while green indicates downregulation. (c) Volcano plots present the results of differential expression analysis between 30 paired ESCA tumors and nontumor tissues in the GSE38129 dataset. Red bubble expresses upregulated gene in ESCA; green bubble is indicative of downregulated gene in ESCA; black bubble represents nonsignificant gene. (d) Heatmap displays the expression patterns of DEGs with adjusted *p* value <0.05 and |log2fold-change|>1 in 30 paired ESCA tumors (T) and nontumor tissues (N) in the GSE38129 dataset. Red is indicative of upregulation while green is indicative of downregulation.

For investigating the genetic alterations during the progression from normal to ESCA, we conducted differential expression analysis between ESCA tumors and normal tissues both in TCGA and GSE38129 datasets. In TCGA cohort, compared with 11 normal tissues, 1221 genes presented remarkable downregulation while 1169 genes displayed prominent upregulation in 160 ESCA tumors in accordance with adjusted *p* value <0.05 and |log2fold-change|>1 (Figures 1(a) and 1(b); Supplementary Table 1). With the same selection criteria, in the GSE38129 dataset, we determined 360 upregulated and 376 downregulated genes in 30 ESCA tumors in comparison to 30 nontumor tissues (Figures 1(c) and 1(d); Supplementary Table 2).

### 3.2. Establishment of a Coexpression Network and Discovery of ESCA-Specific Coexpression Module in the TCGA Dataset

**Figure 2:**
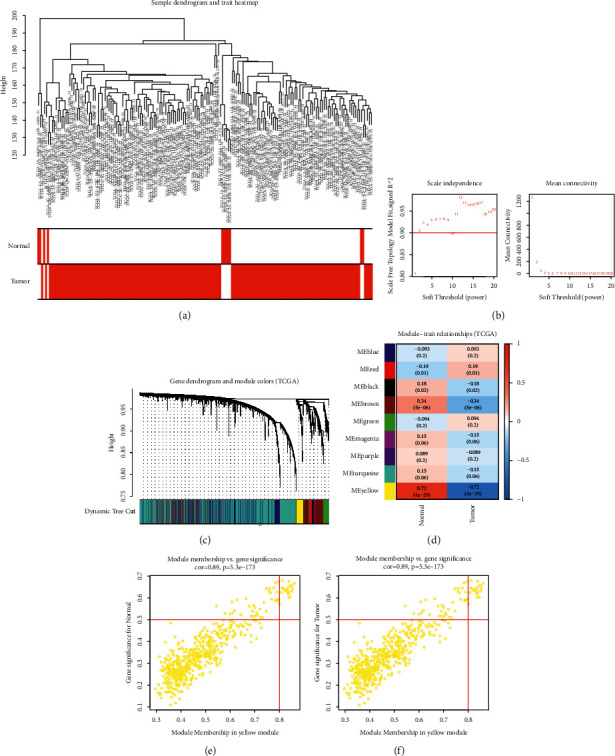
Establishment of a coexpression network and discovery of ESCA-specific coexpression module in the TCGA dataset. (a) Sample cluster analysis. (b) The scale-free network topology (left) as well as mean connectivity (right) under distinct soft threshold power values. (c) Gene dendrogram clustered in accordance with a dissimilarity measure. The upper panel indicates gene tree, and the bottom panel represents gene modules identified by diverse colors. (d) Heatmap visualizes the interaction between coexpression modules and clinical trait-tissue type. The upper number in each cell presents Pearson correlation coefficient between each module and tissue type. Meanwhile, the lower number indicates the *p* value. (e) Scatter plots depict the interaction between module membership and gene significance for normal tissue type for the yellow module. (f) Scatter plots present the interaction between module membership and gene significance for ESCA tumor tissue type for the yellow module.

We first curated gene expression matrix of ESCA patients from TCGA cohort and chose the genes with the top 25% variances for subsequent analysis. No outlier sample was found, and we conducted a sample clustering tree, as shown in Figure 2(a). Thereafter, the soft threshold power value was set as 10 (scale-free topology *R*^2^ = 0.90) for constructing a scale-free network (Figure 2(b)). The adjacency matrix and the topological overlap matrix were separately developed. In total, 9 coexpression modules were clustered in accordance with the average hierarchical clustering and dynamic cutting tree (Figure 2(c)). The association of coexpression modules with clinical trait was analyzed. In Figure 2(d), the yellow module displayed the strongest correlation to tissue type, indicating that this module was strongly linked to ESCA progression. In line with module membership >0.8 and gene significance >0.5, we determined ESCA-specific genes (Figures 2(e) and 2(f)).

### 3.3. Development of a Coexpression Network and Discovery of ESCA-Specific Coexpression Module in the GSE38129 Cohort

**Figure 3:**
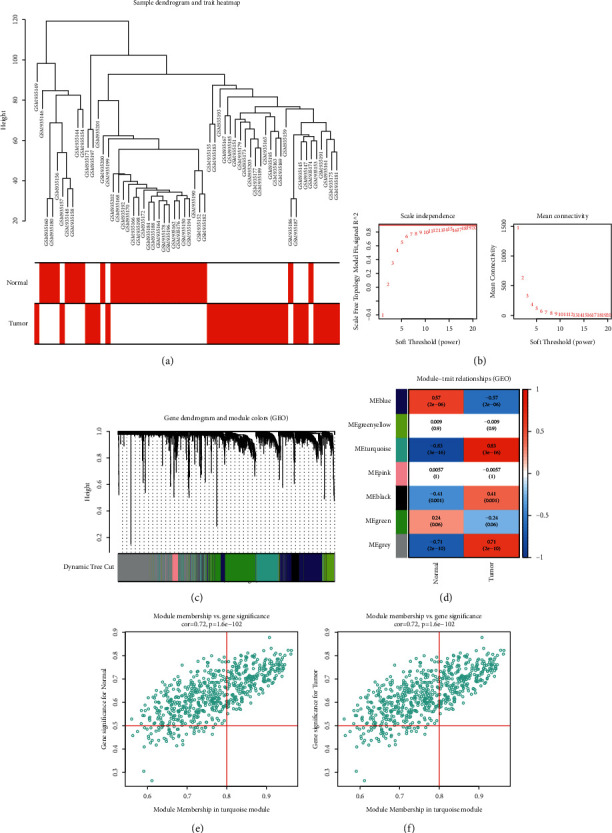
Development of a coexpression network and discovery of ESCA-specific coexpression module in the GSE38129 cohort. (a) Sample cluster analysis of 30 paired ESCA tumors and nontumors. (b) The scale-free network topology (left) and mean connectivity (right) following diverse soft threshold power values. (c) Gene dendrogram clustered in line with a dissimilarity measure. The upper panel presents gene tree and the bottom panel is indicative of gene modules signed by diverse colors. (d) Heatmap displays the relationship between coexpression modules and clinical trait-tissue type. The upper number in each cell presents Pearson correlation coefficient between each module and tissue type. Additionally, the lower number represents the *p* value. (e) Scatter plots showing the association between module membership and gene significance for normal tissue type for the turquoise module. (f) Scatter plots present the correlation between module membership and gene significance for ESCA tumor tissue type for the turquoise module.

The coexpression network was also developed in the GSE38129 dataset. In accordance with the mRNA expression matrix, we selected the genes with the top 25% variances. As shown in Figure 3(a), there was no outlier sample among 30 paired ESCA tumors and nontumors. Afterwards, we established a scale-free network in line with the soft threshold power value = 20 (scale-free topology *R*^2^ = 0.90; Figure 3(b)). Following construction of the adjacency matrix and the topological overlap matrix, we determined 7 coexpression modules on the basis of the average hierarchical clustering and dynamic cutting tree (Figure 3(c)). In Figure 3(d), the turquoise module presented the strongest association with tissue type, demonstrating that this module was strongly linked to ESCA progression. Following module membership >0.8 and gene significance >0.5, ESCA-specific genes were determined (Figures 3(e) and 3(f)).

### 3.4. Identification of ESCA-Specific DEGs and Their Biological Significance

**Figure 4:**
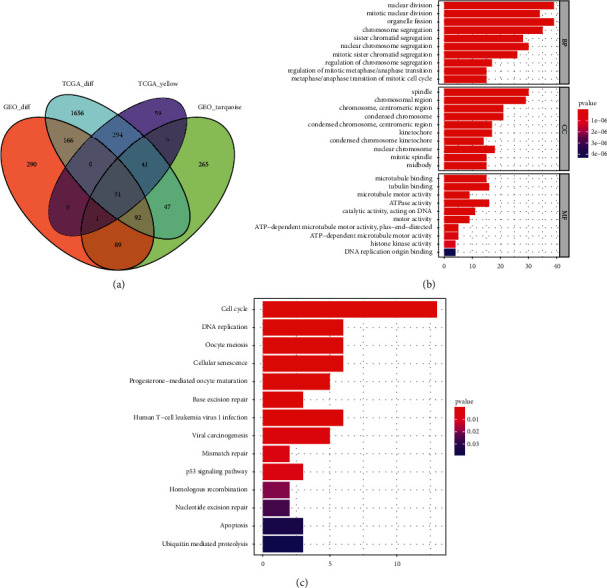
Identification of ESCA-specific DEGs and their biological significance. (a) Venn diagram depicts the intersection of the DEGs and the ESCA-specific genes in TCGA and GSE38129 cohorts. (b) GO enrichment results of the ESCA-specific DEGs. The first 10 enrichment results of BP, CC, and MF categories are separately displayed. (c) KEGG pathway enrichment results of the ESCA-specific DEGs.

For determining ESCA-specific DEGs, we intersected the DEGs and the ESCA-specific genes in TCGA and GSE38129 cohorts. As a result, 91 ESCA-specific DEGs were finally identified (Figure 4(a) and Table 1). Their biological significance was further evaluated through GO and KEGG enrichment analysis. In Figure 4(b) and Table 2, we noted that the ESCA-specific DEGs were remarkably linked to cell cycle progression like chromosome segregation, nuclear division, mitotic nuclear division, and sister chromatid segregation. Additionally, the ESCA-specific DEGs were in relation to ESCA progression-relevant KEGG pathways like cell cycle, DNA replication, cellular senescence, base excision repair, mismatch repair, p53 signaling pathway, homologous recombination, nucleotide excision repair, and apoptosis (Figure 4(c) and Table 3).

### 3.5. Establishment of a PPI Network and Discovery of ESCA-Specific Hub Genes

**Figure 5:**
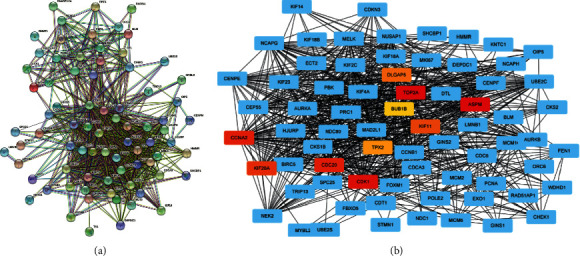
Establishment of a PPI network and discovery of ESCA-specific hub genes. (a) The PPI network of ESCA-specific DEGs through the STRING online tool. (b) Discovery of the ESCA-specific hub genes utilizing CytoHubba plugin. The ten hub genes are marked in orange.

For uncovering the interactions of the ESCA-specific DEGs, we conducted a PPI network in accordance with the STRING online tool. As shown in Figure 5(a), there were close interactions of proteins derived from the ESCA-specific DEGs. Utilizing CytoHubba plugin, we further determined the 10 ESCA-specific hub genes among them, containing *TOP*2*A* (score = 4.45 E + 23), *ASPM* (score = 4.45 E + 23), *CDK*1 (score = 4.45 E + 23), *CDC*20 (score = 4.45 E + 23), *CCNA*2 (score = 4.45 E + 23), *KIF*20* A* (score = 4.45 E + 23), *KIF*11 (score = 4.45 E + 23), *DLGAP*5 (score = 4.45 E + 23), *TPX*2 (score = 4.45 E + 23), and *BUB*1*B* (score = 4.45 E + 23; Figure 5(b)). These ESCA-specific hub genes might exert crucial roles in ESCA progression.

### 3.6. Association of the ESCA-Specific Hub Genes with Pathological Staging of ESCA

**Figure 6:**
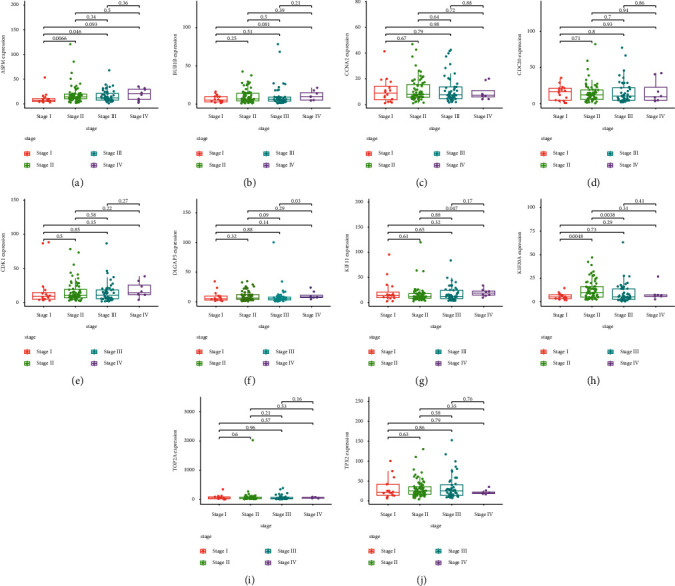
Association of the ESCA-specific hub genes with pathological staging of ESCA patients. (a–j) Box plots depict the difference in (a) *ASPM*, (b) *BUB*1*B*, (c) *CCNA*2, (d) *CDC*20, (e) *CDK*1, (f) *DLGAP*5, (g) *KIF*11, (h) *KIF*20*A*, (i) *TOP*2*A*, and (j) *TPX*2 among diverse pathological staging of ESCA patients in TCGA cohort.

Further analysis was carried out for evaluating the associations of the ten ESCA-specific hub genes with diverse pathological staging of ESCA patients in TCGA cohort. Our results demonstrated that *ASPM*, *BUB*1*B*, *CCNA*2, *CDC*20, *CDK*1, *DLGAP*5, *KIF*11, *KIF*20* A*, *TOP*2*A*, and *TPX*2 presented the different expression in diverse pathological stages across ESCA patients (Figures 6(a)–6(j)). This indicated that the 10 ESCA-specific hub genes were remarkably linked to pathological staging of ESCA.

### 3.7. Association of the ESCA-Specific Hub Genes with ESCA Patients' Prognosis

**Figure 7:**
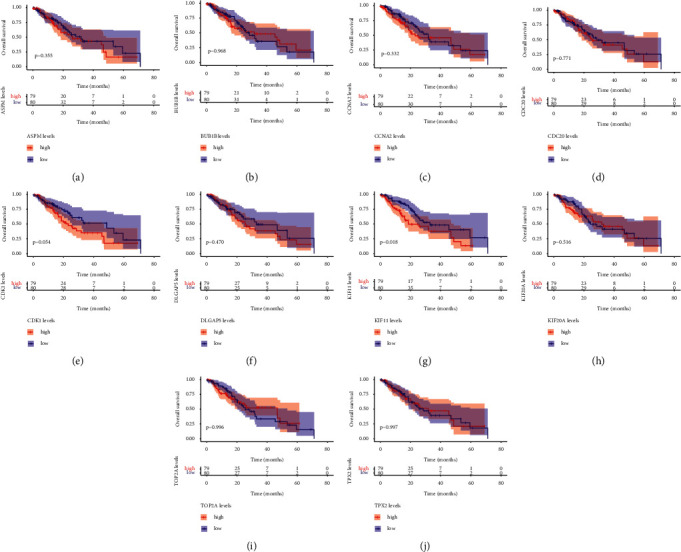
Association of the ESCA-specific hub genes with ESCA patients' prognosis in TCGA cohort. (a–j) Kaplan–Meier curves display the difference in overall survival between high and low expression of (a) *ASPM*, (b) *BUB*1*B*, (c) *CCNA*2, (d) *CDC*20, (e) *CDK*1, (f) *DLGAP*5, (g) *KIF*11, (h) *KIF*20*A*, (i) *TOP*2*A*, and (j) *TPX*2 groups. Survival difference between groups is determined with the log-rank test.

In accordance with the optimal cutoff value of the expression of the ESCA-specific hub genes, we stratified ESCA patients in TCGA cohort into high and expression groups of *ASPM*, *BUB*1*B*, *CCNA*2, *CDC*20, *CDK*1, *DLGAP*5, *KIF*11, *KIF*20*A*, *TOP*2*A*, and *TPX*2 (Figures 7(a)–7(j)). Among them, we noted that ESCA patients in the high expression of the *KIF*11 group presented more undesirable overall survival outcome in comparison to those in the low expression of the *KIF*11 group.

## 4. Discussion

High-throughput sequencing technologies have improved our understanding about the heterogeneity and molecular basis underlying ESCA. At present, available biomarkers for prediction of ESCA patients' survival outcome remain nonsufficiently sensitive and specific. Hence, this study was conducted for discovering novel biomarkers for efficiently predicting ESCA patients' prognosis through the WGCNA algorithm, eventually lowering patients' morbidity and mortality.

Combining the DEGs and ESCA-specific genes in TCGA and GSE38129 cohorts, we determined 91 ESCA-specific DEGs. Our functional enrichment analyses uncovered that the ESCA-specific DEGs were remarkably linked to cell cycle progression and carcinogenic pathways like the p53 signaling pathway, cellular senescence, and apoptosis. This indicated that the ESCA-specific DEGs exerted crucial roles in ESCA progression. Additionally, there were prominent interactions between proteins derived from the ESCA-specific DEGs in accordance with the PPI network. Among them, the 10 ESCA-specific hub genes were finally determined, containing *ASPM*, *BUB*1*B*, *CCNA*2, *CDC*20, *CDK*1, *DLGAP*5, *KIF*11, *KIF*20*A*, *TOP*2*A*, and *TPX*2.

The tumorigenic roles of *ASPM* have been proposed in diverse cancer types. For instance, *ASPM* triggers prostate carcinoma stemness and progression through enhancing the Wnt-Dvl-3-beta-catenin pathway [20]. It is predictive of undesirable prognosis and modulates cellular proliferation in bladder carcinoma [21]. Its upregulation accelerates glioblastoma growth through modulating G1 restriction point progression as well as the Wnt-beta-catenin pathway [22]. Aberrantly expressed *ASPM* regulated by transcriptional factor *FoxM*1 triggers the malignant progression of gliomas [23]. Additionally, it is linked to poor survival outcome as well as induces carcinogenesis in diffuse large B cell lymphoma [24]. Abnormally expressed *ASPM* induces the progression of lung squamous cell carcinoma through modulating *CDK*4 [25]. Increasing evidences demonstrate the crucial role of *ASPM* in cancer progression. For example, *BUB*1*B* accelerates prostate carcinoma proliferation through transcriptionally modulating *MELK* [26]. It triggers hepatocellular carcinoma development through activating *mTORC*1 signaling [27]. It can facilitate extrahepatic cholangiocarcinoma development through *JNK/c-Jun* signaling [28]. Moreover, it participates in the tumorigenicity and radioresistance of glioblastoma [29]. For *CCNA*2, it can be suppressed by *miR-*219-5p, thereby affecting cellular proliferation and cell cycle progression in ESCA [30]. A previous study has proposed that *CDC*20 modulates *E*2*F*1 degradation and thymidylate synthase expression, thereby triggering ESCA chemoresistance [31]. Furthermore, *CDK*1 has been considered as an underlying diagnostic and cancer progression biomarker as well as a drug target for ESCA [32]. Previous bioinformatics and experimental evidences have demonstrated the tumorigenic role of *DLGAP*5 in ESCA [33]. *KIF*11 is essential for spheroid formation of ESCA cells [34]. ScRNA-seq and qPCR analysis uncovered that *KIF*20*A* possesses the potential to diagnose and predict ESCA patients' prognosis [35]. For *TOP*2*A*, experimental data demonstrate that it can affect the resistance of ESCA cells to paclitaxel [36]. Targeting *TPX*2 relieves ESCA progression through weakening tumor growth and invasion [37, 38]. Additionally, its upregulation is mediated by *LINC*00337 and triggers autophagy and resistance to cisplatin in ESCA cells [39]. On the basis of previously published literature and our findings, *ASPM*, *BUB*1*B*, *CCNA*2, *CDC*20, *CDK*1, *DLGAP*5, *KIF*11, *KIF*20*A*, *TOP*2*A*, and *TPX*2 play crucial roles in ESCA progression.

Currently, approach of predicting ESCA patients' prognosis primarily depends on the conventional TNM staging system. Although conventional TNM staging is crucial for diagnosis and treatment interventions, it cannot roundly uncover the intrinsic biological processes and pathological development due to the high heterogeneity in tumor microenvironment and individual discrepancy. Our results demonstrated that the 10 ESCA-specific hub genes (*ASPM*, *BUB*1*B*, *CCNA*2, *CDC*20, *CDK*1, *DLGAP*5, *KIF*11, *KIF*20*A*, *TOP*2*A*, and *TPX*2) presented the remarkable associations with pathological staging, indicating that their roles in ESCA progression. Among them, KIF11 upregulation was indicative of an unfavorable survival outcome of ESCA patients, indicative of the potential of KIF11 as a prognostic indicator of ESCA.

However, there are certain drawbacks in our study. First, the influence of expression alteration of the ESCA-specific hub genes upon patients' prognosis remains to be explored. Hence, in our future, the interactions of the ESCA-specific hub genes with patients' prognosis will be monitored and verified in the large-scale clinical data. Additionally, it is of importance to consider statistical bias because the sample size is relatively small. Moreover, in-depth investigation will be presented for validating the biological significance of the ESCA-specific hub genes through in vitro and in vivo experiments.

## 5. Conclusion

Overall, this study determined the 10 ESCA-specific hub genes as novel markers for ESCA with the WGCNA algorithm based on distinct datasets, which offered promising targets for ESCA precision medicine. Nevertheless, in-depth exploration is required for validating the biological function of the specific hub genes in large‐scale clinical cohorts.

## Figures and Tables

**Table 1:** The list of ESCA-specific DEGs.

ESCA-specific DEGs				
CBX3	FOXM1	DBF4	LMNB2	BLM
KAT2B	DLGAP5	MCM10	ASPM	C1orf112
KIF4A	PCNA	NUSAP1	UBE2S	AURKB
AURKA	KIF18B	CDT1	POLE2	FBXO5
CKS1B	CENPE	BUB1B	OIP5	MYBL2
ECT2	CDC6	BIRC5	CCNA2	CHEK1
HOXB7	CEP55	NCAPH	CCNB1	TFRC
KIF14	MCM6	DTL	MKI67	UBE2C
TRIP13	PRC1	NCAPG	DEPDC1	CDKN3
CITED2	CDCA3	FAM189A2	LMNB1	KIF20 A
MCM2	FEN1	HJURP	NDC1	GINS2
WDHD1	NUDT1	ORC6	KIF11	CENPF
RAD51AP1	RNASEH2A	HMMR	GINS1	STMN1
MAD2L1	PBK	ECHDC2	RUVBL1	EXO1
NDC80	CKS2	FYCO1	CENPM	DNMT1
NEK2	KIF18A	DDX39A	KNTC1	CDK1
KIF2C	SECISBP2L	MELK	CDC20	TPX2
KIF23	RAD54L	SHCBP1	TK1	TOP2A
SPC25				

**Table 2:** The detailed information of GO enrichment results of ESCA-specific genes.

ID	Description	Gene ratio	BgRatio	*P* value	Adjusted *p*	*Q* value	Count
GO:0000280	Nuclear division	39/90	436/18862	4.56 *E* − 40	6.17 *E* − 37	4.56 *E* − 37	39
GO:0140014	Mitotic nuclear division	34/90	296/18862	2.13 *E* − 38	1.44 *E* − 35	1.07 *E* − 35	34
GO:0048285	Organelle fission	39/90	486/18862	3.31 *E* − 38	1.49 *E* − 35	1.10 *E* − 35	39
GO:0007059	Chromosome segregation	35/90	337/18862	5.24 *E* − 38	1.77 *E* − 35	1.31 *E* − 35	35
GO:0000819	Sister chromatid segregation	28/90	199/18862	5.58 *E* − 34	1.51 *E* − 31	1.12 *E* − 31	28
GO:0098813	Nuclear chromosome segregation	30/90	273/18862	4.07 *E* − 33	9.16 *E* − 31	6.77 *E* − 31	30
GO:0000070	Mitotic sister chromatid segregation	26/90	164/18862	6.09 *E* − 33	1.18 *E* − 30	8.70 *E* − 31	26
GO:0051983	Regulation of chromosome segregation	17/90	89/18862	4.02 *E* − 23	6.80 *E* − 21	5.02 *E* − 21	17
GO:0030071	Regulation of mitotic metaphase/anaphase transition	15/90	59/18862	1.50 *E* − 22	2.25 *E* − 20	1.66 *E* − 20	15
GO:0007091	Metaphase/anaphase transition of mitotic cell cycle	15/90	61/18862	2.63 *E* − 22	3.55 *E* − 20	2.63 *E* − 20	15
GO:0005819	Spindle	30/90	381/19520	3.83 *E* − 29	4.34 *E* − 27	2.77 *E* − 27	30
GO:0098687	Chromosomal region	29/90	345/19520	5.72 *E* − 29	4.34 *E* − 27	2.77 *E* − 27	29
GO:0000775	Chromosome, centromeric region	21/90	196/19520	3.40 *E* − 23	1.72 *E* − 21	1.10 *E* − 21	21
GO:0000793	Condensed chromosome	21/90	217/19520	3.00 *E* − 22	1.14 *E* − 20	7.26 *E* − 21	21
GO:0000779	Condensed chromosome, centromeric region	17/90	117/19520	3.24 *E* − 21	9.84 *E* − 20	6.27 *E* − 20	17
GO:0000776	Kinetochore	17/90	137/19520	5.31 *E* − 20	1.35 *E* − 18	8.57 *E* − 19	17
GO:0000777	Condensed chromosome kinetochore	14/90	106/19520	5.13 *E* − 17	1.11 *E* − 15	7.10 *E* − 16	14
GO:0000228	Nuclear chromosome	18/90	250/19520	7.75 *E* − 17	1.47 *E* − 15	9.38 *E* − 16	18
GO:0072686	Mitotic spindle	15/90	157/19520	5.26 *E* − 16	8.89 *E* − 15	5.67 *E* − 15	15
GO:0030496	Midbody	15/90	193/19520	1.17 *E* − 14	1.77 *E* − 13	1.13 *E* − 13	15
GO:0008017	Microtubule binding	15/89	269/18337	3.07 *E* − 12	5.56 *E* − 10	3.91 *E* − 10	15
GO:0015631	Tubulin binding	16/89	368/18337	2.33 *E* − 11	2.11 *E* − 09	1.49 *E* − 09	16
GO:0003777	Microtubule motor activity	9/89	69/18337	4.41 *E* − 11	2.66 *E* − 09	1.87 *E* − 09	9
GO:0016887	ATPase activity	16/89	478/18337	1.09 *E* − 09	4.92 *E* − 08	3.46 *E* − 08	16
GO:0140097	Catalytic activity, acting on DNA	11/89	204/18337	4.22 *E* − 09	1.53 *E* − 07	1.08 *E* − 07	11
GO:0003774	Motor activity	9/89	129/18337	1.26 *E* − 08	3.67 *E* − 07	2.59 *E* − 07	9
GO:0008574	ATP-dependent microtubule motor activity, plus-end-directed	5/89	17/18337	1.42 *E* − 08	3.67 *E* − 07	2.59 *E* − 07	5
GO:1990939	ATP-dependent microtubule motor activity	5/89	35/18337	6.96 *E* − 07	1.57 *E* − 05	1.11 *E* − 05	5
GO:0035173	Histone kinase activity	4/89	16/18337	9.02 *E* − 07	1.81 *E* − 05	1.28 *E* − 05	4
GO:0003688	DNA replication origin binding	4/89	23/18337	4.28 *E* − 06	7.06 *E* − 05	4.97 *E* − 05	4

**Table 3:** The detailed information of KEGG pathways enriched by ESCA-specific genes.

ID	Description	Gene ratio	BgRatio	*P* value	Adjusted *p*	*Q* value	Count
hsa04110	Cell cycle	13/43	126/8104	4.08 *E* − 14	2.08 *E* − 12	1.59 *E* − 12	13
hsa03030	DNA replication	6/43	36/8104	2.69 *E* − 08	6.85 *E* − 07	5.23 *E* − 07	6
hsa04114	Oocyte meiosis	6/43	131/8104	5.94 *E* − 05	0.001009	0.000771	6
hsa04218	Cellular senescence	6/43	156/8104	0.000156	0.001923	0.001468	6
hsa04914	Progesterone-mediated oocyte maturation	5/43	102/8104	0.000189	0.001923	0.001468	5
hsa03410	Base excision repair	3/43	33/8104	0.00068	0.005776	0.004411	3
hsa05166	Human T cell leukemia virus 1 infection	6/43	222/8104	0.001031	0.007513	0.005737	6
hsa05203	Viral carcinogenesis	5/43	204/8104	0.004257	0.02714	0.020726	5
hsa03430	Mismatch repair	2/43	23/8104	0.006483	0.034097	0.026039	2
hsa04115	p53 signaling pathway	3/43	73/8104	0.006686	0.034097	0.026039	3
hsa03440	Homologous recombination	2/43	41/8104	0.019784	0.091726	0.070049	2
hsa03420	Nucleotide excision repair	2/43	47/8104	0.025565	0.108651	0.082974	2
hsa04210	Apoptosis	3/43	136/8104	0.035027	0.137412	0.104938	3
hsa04120	Ubiquitin mediated proteolysis	3/43	142/8104	0.039049	0.14225	0.108633	3

## Data Availability

The data used to support the findings of this study are included within the supplementary information files.

## Abbreviations

ESCA: Esophageal cancer
TNM: Tumor, node, metastasis
WGCNA: Weighted gene coexpression network analysis
TCGA: The Cancer Genome Atlas
GEO: Gene Expression Omnibus
DEGs: Differentially expressed genes
GO: Gene Ontology
KEGG: Kyoto Encyclopedia of Genes and Genomes
BP: Biological process
CC: Cellular component
MF: Molecular function
PPI: Protein-protein interaction
STRING: Search Tool for the Retrieval of Interacting Genes/Proteins.

## Supplementary Materials

Supplementary Materials Supplementary Table 1. The detailed information of DEGs between ESCA tumors and normal tissues in TCGA cohort. Supplementary Table 2. The detailed information of DEGs between ESCA tumors and normal tissues in the GSE38129 dataset. .
