# Supplementary material for: Weighted Gene Correlation Network Analysis Identifies Specific Functional Modules and Genes in Esophageal Cancer
Source: J Oncol. 2021 Dec 27;2021:8223263. doi: 10.1155/2021/8223263 (PMC8723838; doi:10.1155/2021/8223263)
Supplement: Supplementary Materials — Supplementary Table 1. The detailed information of DEGs between ESCA tumors and normal tissues in TCGA cohort. Supplementary Table 2. The detailed information of DEGs between ESCA tumors and normal tissues in the GSE38129 dataset. . [file 8223263.f1.zip › 8223263.f1/Supplementary table 1.pdf]

Supplementary table 1. The detailed information of DEGs between ESCA tumors and normal tissues in TCGA cohort.

ID	logFC	AveExpr	t	P.Value
TPX2	3.100966191	6.713060899	12.33809323	1.78E-25
NEK2	3.082716264	4.197880299	11.41772488	7.56E-23
KIF18B	2.989041773	5.126892699	11.39434082	8.81E-23
UBE2C	3.055240616	5.55299974	11.1745889	3.71E-22
BIRC5	3.041871898	5.063380147	11.00005687	1.16E-21
BUB1	2.786567419	5.205940831	10.83029452	3.50E-21
KIF4A	2.796139841	4.801704968	10.7192843	7.19E-21
PGA4	-7.195333751	-3.889373426	-10.71871028	7.22E-21
NCAPH	2.767874699	4.362417898	10.67645615	9.50E-21
KIFC1	2.625496735	4.611675883	10.63737468	1.22E-20
EXO1	2.877478964	3.820513429	10.62093259	1.36E-20
KIF23	2.470268676	5.116677858	10.59512	1.61E-20
KIF2C	2.58650047	5.057273651	10.59189731	1.64E-20
TROAP	2.713930634	4.579350385	10.56591814	1.94E-20
NUF2	2.887325504	3.940917499	10.54694176	2.20E-20
DLGAP5	2.80601708	4.69724106	10.4862287	3.25E-20
CDC45	2.983537502	3.796527196	10.48245585	3.33E-20
CCKAR	-5.679725654	-3.457995171	-10.4432435	4.29E-20
BUB1B	2.720591514	4.888372615	10.43378625	4.56E-20
MELK	2.671251042	4.709907962	10.38138808	6.40E-20
CENPA	2.977149613	3.207867968	10.26039278	1.39E-19
AURKB	2.955991145	4.313623259	10.23440784	1.65E-19
CDCA5	2.798166631	4.568058689	10.22286248	1.78E-19
UBE2T	2.517558374	4.16936144	10.1602787	2.65E-19
GTSE1	2.458733036	4.266268807	10.14491434	2.93E-19
KIF18A	2.663734251	3.819925566	10.13947772	3.03E-19
NUSAP1	2.541902422	5.668934261	10.1308345	3.20E-19
KIF14	2.817827292	4.342246435	10.12996984	3.22E-19
CENPF	2.737382993	6.822704727	10.11844204	3.47E-19
CDC20	2.832993998	5.578501183	10.11364144	3.58E-19
CEP55	2.837587342	5.350958494	10.08810968	4.21E-19
CKAP2L	2.703709594	4.242547318	10.08232081	4.37E-19
CDK1	2.442452664	5.623284555	10.07247745	4.66E-19
ASPM	2.775845999	5.695755779	10.07112357	4.70E-19
MCM10	2.925621016	3.689437272	10.06824389	4.79E-19
CLSPN	2.283316026	4.323212334	10.0427611	5.63E-19
PDILT	-5.442997467	-3.717823109	-10.03334822	5.98E-19
DEPDC1	2.7309022	4.466400028	9.978967499	8.47E-19
MYBL2	3.015927006	6.427914251	9.908669504	1.33E-18
CHIA	-7.241670634	-3.56948624	-9.829448543	2.20E-18
KIF11	2.260867933	5.799372842	9.796076427	2.72E-18
SKA3	2.681463742	3.950515909	9.773846079	3.13E-18
ASF1B	2.50922918	4.762263773	9.760875923	3.40E-18
HJURP	2.752706679	4.235234008	9.600912854	9.36E-18
C16orf89	-5.855774903	-1.095843728	-9.600860509	9.37E-18
CDCA2	2.609980886	3.815820825	9.594714525	9.74E-18
PGA5	-9.099492378	-2.781306811	-9.589609189	1.01E-17
RRM2	2.736106853	6.342134255	9.589208058	1.01E-17
MAD2L1	2.281962926	4.939386194	9.581729662	1.06E-17
NCAPG	2.456828831	4.669301644	9.579530786	1.07E-17

CKMT2	-3.632011249	-0.07711993	-9.576718356	1.09E-17
ECT2	2.382943374	6.715365583	9.534938686	1.42E-17
CKS2	2.365566153	5.095823731	9.481275562	1.99E-17
KIF20A	2.778835254	4.897263622	9.464293718	2.22E-17
TMED6	-3.367721167	-0.321791065	-9.462411235	2.24E-17
HROB	2.442321474	3.162984042	9.454505971	2.36E-17
HOXC9	3.805074702	1.234093332	9.454383468	2.36E-17
ANLN	2.826659933	6.484597865	9.451286192	2.41E-17
RAD54L	2.306193771	3.244724027	9.405214357	3.22E-17
ATP4B	-8.11483615	-2.818128331	-9.402227425	3.28E-17
CDKN3	2.441015417	3.710543154	9.385776411	3.63E-17
GPER1	-3.912265954	1.418990651	-9.380312787	3.76E-17
WDR62	2.30697301	4.512801319	9.373729608	3.92E-17
POLQ	2.537448746	4.390923343	9.36841379	4.05E-17
MKI67	2.568080952	7.741270397	9.367200201	4.08E-17
ORC1	2.567219656	3.508126281	9.298264975	6.29E-17
IQGAP3	2.482318686	5.931304293	9.292182692	6.53E-17
STIL	2.242000136	4.841547396	9.258640586	8.06E-17
SLC1A2	-4.46343357	-0.093975723	-9.252886853	8.36E-17
PTTG1	2.677352773	4.81978777	9.218124897	1.04E-16
PLK1	2.350398904	5.812448695	9.21274798	1.07E-16
PBK	2.587637345	4.190930224	9.163255315	1.46E-16
RAD51AP1	2.380828858	3.936624943	9.160981654	1.48E-16
TK1	2.574953664	6.127411451	9.153661028	1.55E-16
GHRL	-4.898859141	-0.43374209	-9.145465349	1.63E-16
CDCA8	2.149457196	4.813443438	9.143642392	1.65E-16
NDC80	2.496770556	4.01021961	9.136549368	1.73E-16
PKMYT1	2.842566401	4.73892496	9.133196739	1.76E-16
STX12	-1.57616473	5.63078495	-9.122751985	1.88E-16
SGO1	2.169207309	3.182877925	9.113344784	2.00E-16
TRIM50	-5.33156867	-2.81254641	-9.110514447	2.03E-16
HOXB7	3.280761639	3.457717547	9.077042898	2.50E-16
GINS1	2.328527704	4.370119019	9.068660903	2.63E-16
FANCA	1.984893302	4.760635243	9.064601247	2.70E-16
DTL	2.460936898	4.722006647	9.063398939	2.72E-16
BLM	2.113162382	3.857808237	9.061593011	2.75E-16
SIK2	-1.837702917	5.93426096	-9.06145912	2.76E-16
PRC1	2.100530602	5.849676133	9.056049728	2.85E-16
CCKBR	-5.959890253	-2.781483528	-9.034622345	3.25E-16
PGA3	-8.946028267	-2.856215777	-9.032809882	3.29E-16
E2F7	2.949259155	3.955277161	8.991259245	4.26E-16
SHCBP1	2.252062743	3.87250988	8.986689893	4.38E-16
CDC6	2.858713855	4.863646557	8.948645878	5.54E-16
KNL1	2.272249779	4.998701551	8.943179938	5.73E-16
TOP2A	2.979956513	7.755674745	8.9415318	5.79E-16
MTFR2	2.418028761	2.520011996	8.935073686	6.03E-16
CDCA3	2.267852732	4.167098318	8.933982015	6.07E-16
CDC25C	2.327682323	2.530312769	8.926859812	6.34E-16
PRR11	2.47499162	5.078465624	8.916241042	6.77E-16
SKA1	2.512085588	3.163681726	8.907497142	7.15E-16
AURKA	2.174876408	4.912395405	8.902282445	7.38E-16
ARHGAP11A	2.059473004	5.372917058	8.878653715	8.54E-16
CENPI	2.2025043	2.995866283	8.844557626	1.05E-15

CCNA2	2.22333008	5.271714268	8.827997718	1.17E-15
CCNB1	2.323627606	5.516448822	8.826930209	1.17E-15
SGO2	1.90491368	3.91829909	8.826144631	1.18E-15
HOXC8	3.818778294	1.172284254	8.820211422	1.22E-15
PCLAF	2.382663868	4.861039623	8.809702259	1.31E-15
FOXM1	2.551306736	6.317266702	8.806685315	1.33E-15
EME1	2.197461103	2.265832958	8.763774085	1.73E-15
PLK4	2.012514261	4.087511058	8.757898754	1.80E-15
CCNB2	2.470166247	5.112073006	8.7406317	2.00E-15
KIF15	2.148537958	3.847940488	8.732042235	2.10E-15
ATP4A	-8.004379658	-2.12296728	-8.724148649	2.21E-15
SIGLEC11	-4.353850863	-1.791915029	-8.718633639	2.28E-15
KCNE2	-6.096532428	-1.676967793	-8.717063806	2.31E-15
RECQL4	2.165227446	5.113831396	8.709577344	2.42E-15
ORC6	2.110018015	3.37132536	8.685590725	2.80E-15
CENPE	2.182428306	4.973000468	8.675192278	2.98E-15
UHRF1	2.258275016	4.774298982	8.619223754	4.20E-15
CKAP2	2.064596217	5.649320117	8.604742814	4.58E-15
RAD51	2.214096121	3.423199687	8.586546572	5.12E-15
ESRRB	-3.988457942	-1.621386138	-8.575577842	5.48E-15
TICRR	2.484719787	4.539905177	8.564550152	5.86E-15
CENPK	2.038136364	3.265550868	8.556267028	6.16E-15
CENPM	2.173487311	3.009647041	8.514060884	7.96E-15
GRIA4	-4.290141779	-2.187425452	-8.47771233	9.93E-15
UBE2S	2.091238126	5.425226833	8.46773845	1.05E-14
KPNA2	1.891923171	7.205667632	8.422828142	1.38E-14
MTURN	-2.281980603	4.415801303	-8.405184686	1.54E-14
DDIAS	2.453465457	3.625584679	8.333350969	2.38E-14
CDT1	2.375997074	4.883970646	8.33221523	2.39E-14
LMNB1	1.921036257	5.537414486	8.32091166	2.56E-14
TRIP13	2.649312761	4.727521222	8.316751559	2.63E-14
HOXC6	3.359431665	2.05142462	8.304803183	2.82E-14
ALAD	-1.526292209	5.406079879	-8.298923054	2.92E-14
AQP4	-5.599154113	-2.686513421	-8.29515117	2.99E-14
MLYCD	-1.352114139	3.421236471	-8.293120803	3.03E-14
C1orf112	1.412218654	3.451899034	8.269897953	3.48E-14
TBC1D31	1.522638208	3.724705568	8.258398682	3.73E-14
HMMR	2.36705541	4.508344578	8.242948937	4.09E-14
CIP2A	2.096560769	3.917223647	8.232375043	4.36E-14
ETNPPL	-5.07554088	-3.024222137	-8.228011345	4.47E-14
TIMELESS	1.672019099	5.616116851	8.195219088	5.44E-14
CKS1B	1.764791836	4.843729451	8.176404622	6.09E-14
LIPF	-11.14392476	-0.838610797	-8.153569703	6.98E-14
PARPBP	1.940974084	3.535895046	8.146601431	7.28E-14
SIDT2	-1.679703468	5.671471921	-8.129767716	8.05E-14
RGMB	-1.985575364	3.912412862	-8.123374388	8.36E-14
WDHD1	1.951986244	4.408991109	8.119172093	8.57E-14
CKM	-4.420330373	-1.772276598	-8.049744994	1.30E-13
FANCI	1.781008744	5.830012768	8.040003058	1.37E-13
SPC24	1.981000709	2.984522917	8.018770689	1.56E-13
TEDC2	2.301104229	2.378826107	7.939125385	2.49E-13
CCNF	1.812600366	4.879605632	7.930976184	2.62E-13
CDC25B	2.192633123	6.808791499	7.924601622	2.72E-13

MTBP	1.693996561	3.317895486	7.922468063	2.75E-13
DEPDC1B	2.255973102	3.738169949	7.914234864	2.89E-13
CRY2	-1.444927616	4.764918418	-7.894540865	3.24E-13
MCM4	1.895707988	6.965796538	7.891825039	3.30E-13
FANCB	1.868784443	1.425132966	7.884437993	3.44E-13
SH3GL2	-4.764170756	-2.460630013	-7.869407438	3.76E-13
KNTC1	1.588410494	5.759626895	7.863779681	3.89E-13
KIF22	1.378906689	5.604830135	7.85944707	3.99E-13
HASPIN	1.879253194	2.914300347	7.854408546	4.11E-13
ERCC6L	2.076980826	3.010253551	7.834590123	4.61E-13
BRIP1	1.860729602	3.866553213	7.789726844	6.00E-13
PIMREG	2.432635805	2.898448625	7.773139902	6.61E-13
NBPF12	-1.933938723	3.543546941	-7.772520246	6.64E-13
CHEK1	1.769399715	4.375322245	7.771896869	6.66E-13
CBLIF	-7.737890459	-1.965951322	-7.770819228	6.70E-13
HOXC11	4.069845657	1.425393875	7.762469675	7.04E-13
FAM72B	2.403116586	0.950167877	7.737665125	8.14E-13
XRCC2	2.230417708	3.288311989	7.732298893	8.40E-13
EZH2	1.588930314	4.666539644	7.726789961	8.67E-13
FAM72A	2.109197221	0.573978315	7.721901908	8.92E-13
GPR155	-3.157082185	4.089255766	-7.705915828	9.79E-13
NEIL3	2.512497074	2.05872128	7.661993897	1.26E-12
MCM2	2.230943077	6.214150174	7.640306905	1.43E-12
FAM72D	2.208434076	0.263597975	7.624723197	1.57E-12
CENPW	2.235445526	3.454807231	7.613529218	1.67E-12
CPA2	-5.885737965	-2.358591952	-7.609294855	1.72E-12
TDO2	3.190745114	1.49467616	7.606709857	1.74E-12
PLCXD3	-5.267079593	-1.613629753	-7.604351512	1.77E-12
ULBP2	3.791592148	2.308699899	7.598748087	1.82E-12
CCDC150	1.996928822	1.725904996	7.596990932	1.84E-12
PCNA	1.627642054	6.872202535	7.589930018	1.92E-12
DUSP19	-2.143661352	2.133223498	-7.566774485	2.19E-12
RNASEH2A	1.73396975	4.339233148	7.551011496	2.40E-12
LAMP3	3.151628148	4.591098186	7.531975024	2.68E-12
TYMS	1.862048861	4.951026458	7.524046912	2.81E-12
BRCA1	1.7712884	5.1215684	7.498481129	3.25E-12
RACGAP1	1.542260793	5.82315398	7.495764228	3.30E-12
GKN1	-8.020867939	-1.986610618	-7.49204704	3.38E-12
ESPL1	2.295519295	5.34543584	7.480466643	3.61E-12
OIP5	2.020083304	2.01074492	7.470142838	3.83E-12
HDC	-3.750875736	-0.363355031	-7.420059493	5.10E-12
MND1	2.034946205	1.75493364	7.411453516	5.36E-12
B3GAT1	-4.306828199	-0.53918526	-7.396348501	5.84E-12
CDCA7	2.44779405	5.320144571	7.394641057	5.90E-12
IBSP	3.891937779	-0.366165737	7.388909368	6.10E-12
SLC25A4	-1.976033955	4.25814728	-7.357790338	7.28E-12
GINS4	2.133371836	3.044445053	7.352521711	7.50E-12
TTK	1.799695353	4.552329049	7.342922703	7.92E-12
NUDT1	1.816035707	3.652530587	7.338339603	8.13E-12
ACAT1	-1.565927584	4.755207369	-7.334823351	8.30E-12
SPC25	1.631128432	2.867509305	7.331450741	8.46E-12
ESM1	3.583255288	1.923062933	7.313764802	9.35E-12
DDX39A	1.251870403	6.152321183	7.291312094	1.06E-11

MT1M	-4.171282558	0.383778393	-7.286747199	1.09E-11
GINS2	2.206557501	3.585367042	7.283885497	1.11E-11
SPAG5	2.076020573	5.379422088	7.280811015	1.13E-11
ALDH6A1	-1.910518147	4.736583932	-7.263592221	1.24E-11
NRM	1.695910031	4.604795123	7.239870096	1.42E-11
TACC3	1.713560346	5.840675883	7.23904577	1.43E-11
TBC1D14	-1.568408201	4.830546356	-7.223670274	1.56E-11
FAM111B	2.107854538	4.049256497	7.221409645	1.58E-11
LRP8	2.473442535	4.753366	7.213933164	1.65E-11
PIF1	1.799406857	2.847971788	7.206313705	1.72E-11
RBL1	1.430618912	4.673167205	7.204985644	1.73E-11
TMEM161B	-1.299891457	4.039570747	-7.204627204	1.73E-11
RAB11FIP2	-1.474743181	4.548062752	-7.201577975	1.76E-11
ESRRG	-4.932711227	-0.374675456	-7.184587595	1.94E-11
E2F1	1.559674712	4.381896188	7.1832804	1.96E-11
MMP12	4.243223361	4.147512617	7.178451468	2.01E-11
H2BC9	2.625345179	0.344659661	7.170521402	2.10E-11
SNRPG	1.167068943	5.414102532	7.156678423	2.27E-11
CYRIB	1.264689987	6.235857521	7.156631992	2.27E-11
TRAIP	1.551632577	2.345799891	7.138592225	2.52E-11
CHEK2	1.411012897	3.417849754	7.129873781	2.64E-11
BRCA2	1.786690728	4.471895745	7.125664629	2.70E-11
KLF15	-4.076631601	0.564898367	-7.125118755	2.71E-11
CADM2	-4.734280598	-1.675690842	-7.103408912	3.06E-11
F12	2.741017673	3.119312351	7.093586363	3.24E-11
RFC3	1.614236455	4.683583081	7.089549005	3.31E-11
FEN1	1.580425749	5.14306008	7.088513024	3.33E-11
ANGPTL3	-2.92807123	-1.135338988	-7.079041971	3.51E-11
RCC2	1.355546759	7.784349409	7.054292387	4.03E-11
MTFR1L	-1.032990845	4.703510123	-7.033639954	4.52E-11
PLIN5	-3.532015106	-0.196655827	-7.03050067	4.60E-11
MMP11	4.316987259	5.417845183	7.026173295	4.72E-11
MYRIP	-4.204431578	0.283886046	-7.014435258	5.03E-11
PBXIP1	-1.498282855	6.122605717	-6.999763739	5.46E-11
SYNJ2BP	-1.313436866	5.476476019	-6.995518854	5.59E-11
PRIM2	1.237467948	3.982545786	6.991764106	5.71E-11
SEC14L5	-2.712778434	-0.234584264	-6.986416582	5.88E-11
HMGB3	1.924366508	5.516367861	6.985212691	5.92E-11
CIRBP	-1.131160137	6.309917099	-6.981217433	6.06E-11
RELT	1.652726706	3.254179316	6.977290856	6.19E-11
CXCL8	4.096523808	5.022109172	6.963283969	6.69E-11
RCC1	1.482361936	5.445659091	6.946819309	7.33E-11
ARHGDIG	-4.194551804	-1.755956961	-6.933464055	7.89E-11
FRMD1	-5.311264852	-1.594712017	-6.907390304	9.11E-11
CTIF	-1.671005361	4.887666356	-6.899470508	9.52E-11
MRGBP	1.20083865	4.338474287	6.896706423	9.66E-11
CDK2	1.184543189	4.921225703	6.884932872	1.03E-10
ALYREF	1.185073176	5.383385968	6.873158663	1.10E-10
STMN1	1.799495598	6.90101948	6.854544137	1.22E-10
CLDN7	3.23999702	6.104135177	6.849071984	1.26E-10
CBFB	1.139435503	5.927494951	6.84161683	1.31E-10
CDCA4	1.840230433	4.857163365	6.838820331	1.33E-10
AUNIP	1.773568957	1.938114138	6.826077511	1.43E-10

SNRPB	1.247149252	7.650147135	6.787080089	1.76E-10
CKB	-3.164945314	5.422729552	-6.773844558	1.90E-10
RMI2	1.944075939	3.855987506	6.760350826	2.04E-10
CNTD1	-1.925315677	0.783283998	-6.75814643	2.07E-10
POLE2	1.412716744	3.156609699	6.757674904	2.07E-10
ZWINT	1.619627897	5.07291958	6.752427073	2.13E-10
ADAMTS12	3.256858523	4.021586527	6.748817208	2.17E-10
CUX2	-3.990913262	-1.910953365	-6.742140495	2.25E-10
CAB39L	-2.148278061	3.253896748	-6.738912707	2.29E-10
SMIM14	-1.493014262	5.589083237	-6.737685071	2.31E-10
CTXND1	-4.099302947	-1.727799955	-6.73076087	2.40E-10
SECISBP2L	-1.236203477	5.518906542	-6.726667785	2.45E-10
GKN2	-6.195246642	-2.516298487	-6.726628326	2.45E-10
DGKD	-2.181342206	5.458054277	-6.723990687	2.49E-10
SPDL1	1.276381923	4.131928711	6.713504592	2.63E-10
NME1	1.487576149	5.448029224	6.701698569	2.81E-10
TRIM59	1.623182158	3.870558363	6.700493289	2.83E-10
SLC9A4	-5.620573161	0.02408022	-6.697850765	2.87E-10
SUCLG2	-1.64917799	5.420049375	-6.695008351	2.91E-10
PRKN	-2.667725283	0.402773049	-6.690167791	2.99E-10
DSCC1	1.581573574	3.428360407	6.68447938	3.08E-10
MCM7	1.700705104	7.012622872	6.681730212	3.13E-10
LMNB2	1.490794958	7.029903001	6.677737831	3.20E-10
SNX10	2.232272898	4.263966056	6.676646629	3.22E-10
ASPA	-3.360282908	-0.788659768	-6.67540168	3.24E-10
CENPU	1.767269718	4.240957911	6.669319076	3.35E-10
JPT1	1.529376677	6.686794883	6.668474061	3.36E-10
PLAU	2.895794011	6.297628821	6.667020522	3.39E-10
PDIA2	-5.314900269	-0.314989662	-6.661584028	3.49E-10
MFSD4A	-4.441512919	2.851261611	-6.658371151	3.55E-10
LY6E	2.18608746	7.882097079	6.647028938	3.78E-10
PCSK9	3.63319293	4.095157867	6.646427109	3.79E-10
PDCD4	-1.638627582	5.978547518	-6.641768737	3.89E-10
UBL3	-1.744895186	5.839306974	-6.631001772	4.12E-10
ERFE	2.532551244	1.263992427	6.628267416	4.18E-10
HELLS	1.482666948	5.288792773	6.627923721	4.19E-10
ESCO2	1.909905827	3.728071018	6.626758224	4.21E-10
RFC4	1.670717921	4.462562793	6.621733204	4.33E-10
CDC7	1.492318755	3.493955562	6.620207739	4.37E-10
DNMT1	1.170058395	6.810564766	6.618311443	4.41E-10
HDGF	1.043003532	8.516311586	6.605502652	4.73E-10
KCNJ13	-2.44929487	-0.743340512	-6.598747338	4.90E-10
ACACB	-2.435676799	4.178118912	-6.595150658	5.00E-10
HOXA10	3.193291401	2.913205716	6.591994459	5.08E-10
CENPL	1.119731809	3.551416505	6.580547205	5.41E-10
FOXS1	2.287200372	0.380596807	6.578658973	5.46E-10
TEAD4	1.456763263	4.443797841	6.547557107	6.45E-10
HOXC10	4.074939868	2.882243042	6.543945911	6.58E-10
ZHX3	-1.241494184	5.210922205	-6.543332072	6.60E-10
JADE1	-1.82224538	4.822075588	-6.541025984	6.68E-10
IGF2BP3	4.482387118	3.374357016	6.537428916	6.81E-10
PRR7	2.025606941	3.640410762	6.528016307	7.17E-10
POLD1	1.258904689	5.089004181	6.525276523	7.27E-10

C6	-4.440720673	-2.139299073	-6.519464776	7.50E-10
RIMS4	-4.058260786	-1.987703717	-6.514516976	7.70E-10
AK3	-1.130889039	6.259728009	-6.495929893	8.51E-10
RUVBL1	1.365526756	5.47923372	6.493117606	8.63E-10
TUBB	1.193816263	9.4431709	6.490928656	8.74E-10
DNA2	1.441677538	3.60931035	6.45486205	1.06E-09
FAM107A	-3.559363216	1.471013594	-6.443387414	1.13E-09
GPRIN1	2.155994322	4.019315108	6.441381079	1.14E-09
FBXO5	1.181782067	3.707944186	6.441120092	1.14E-09
SCUBE2	-3.083439649	1.425195125	-6.43309561	1.19E-09
DNER	-4.507338637	-0.360812145	-6.432313266	1.19E-09
ACO2	-1.105676359	6.56011211	-6.424958483	1.24E-09
CBX3	1.117654091	7.21649857	6.421104733	1.27E-09
P2RY14	-2.826979888	0.734351129	-6.418771308	1.28E-09
POC1A	1.523197543	3.087764867	6.414337412	1.31E-09
BTD	-1.516473605	3.464171032	-6.399790962	1.42E-09
CENPN	1.276024112	4.302317822	6.397553378	1.43E-09
CHAF1A	1.188745686	4.819647523	6.390434178	1.49E-09
GABRD	2.49952338	0.04462	6.388190774	1.51E-09
MDFI	2.4118148	4.874015048	6.380173647	1.57E-09
MT1G	-4.184912752	3.223877515	-6.37652383	1.60E-09
LIG1	1.201529872	5.13299847	6.37007939	1.66E-09
ANP32E	1.122256011	6.13648082	6.369041897	1.67E-09
SST	-5.075206998	-2.766101596	-6.357389532	1.77E-09
ACADSB	-1.625956457	4.406282583	-6.354193155	1.80E-09
FOXN3	-1.242019588	6.445937476	-6.346836358	1.87E-09
MT-ND6	-1.985939699	9.044135934	-6.344546851	1.90E-09
TMEM26	2.274441631	0.424783223	6.338913674	1.95E-09
E2F2	1.84797326	3.876528391	6.338459196	1.96E-09
ZWILCH	1.229817113	4.904104535	6.337394896	1.97E-09
ATOH8	-2.888408955	0.679619363	-6.327232495	2.08E-09
ARHGEF37	-2.036947894	4.342162212	-6.315530204	2.21E-09
DKC1	1.050629312	6.427367048	6.314410091	2.22E-09
DBT	-1.234049515	5.060181363	-6.304637373	2.34E-09
MMS22L	1.199996275	4.610822337	6.286475646	2.57E-09
ZBTB16	-4.409311811	0.722812742	-6.276504399	2.71E-09
EIF4A3	1.026081384	6.472207178	6.27494049	2.73E-09
PNPLA7	-2.583104969	2.377987344	-6.274920733	2.73E-09
TTYH3	1.737951164	7.764588422	6.27295673	2.76E-09
CD3EAP	1.290804065	2.4072264	6.272589688	2.77E-09
RAB27A	-1.409679663	4.937064273	-6.270685523	2.80E-09
DGLUCY	-1.240948995	4.906281041	-6.269849811	2.81E-09
ETV4	2.689975216	5.146990548	6.265257713	2.88E-09
DPT	-3.663892956	1.376356802	-6.264834217	2.88E-09
CTHRC1	2.9077801	4.542429158	6.263617789	2.90E-09
BAX	1.068950505	4.953439092	6.260257291	2.95E-09
LIFR	-3.221037619	3.905884369	-6.256632127	3.01E-09
FANCD2	1.512906485	4.437955238	6.255011137	3.03E-09
C3orf18	-1.992082353	0.90130958	-6.247025336	3.16E-09
KL	-2.820232242	0.293551012	-6.246603347	3.17E-09
LEPR	-2.540866882	3.569982255	-6.240727463	3.27E-09
CLEC5A	2.658986897	0.979484966	6.233700959	3.39E-09
PXMP2	-1.538445579	2.958226197	-6.231851921	3.42E-09

FAAP24	1.335233011	2.09450097	6.229301273	3.47E-09
MYOC	-5.292150838	-1.872251307	-6.217018776	3.70E-09
SLC26A7	-3.717328187	-1.355668579	-6.212039447	3.80E-09
ACTL6A	1.274658286	6.111206191	6.210490463	3.83E-09
FGD6	1.549825787	5.720885621	6.206405832	3.91E-09
SAPCD2	1.987261946	5.307543418	6.196048522	4.12E-09
GNG7	-2.696855185	1.46140894	-6.191325453	4.23E-09
NCAPG2	1.536238109	5.175956034	6.190398997	4.25E-09
ECI2	-1.522997657	4.375473497	-6.186664514	4.33E-09
GRIP2	-2.919158719	0.495786743	-6.183807034	4.40E-09
TCF19	1.324511167	4.874485329	6.182851257	4.42E-09
SLC16A13	1.618279581	2.719612905	6.177978109	4.53E-09
MICB	2.403684998	2.884626902	6.174713971	4.61E-09
C5orf34	1.643772437	2.011820479	6.153106352	5.15E-09
PBLD	-2.374509675	3.032585296	-6.150737478	5.22E-09
CHTF18	1.366985421	4.366809024	6.150672082	5.22E-09
GSTM5	-2.503540634	0.02671534	-6.147210033	5.31E-09
CPEB3	-1.501176237	3.225284809	-6.145864933	5.35E-09
CHAF1B	1.491416726	3.741258977	6.14374302	5.41E-09
MT-ND5	-1.786081334	11.10755086	-6.135502535	5.64E-09
NICN1	-1.145908502	2.773307233	-6.134129174	5.68E-09
TMC6	1.280323287	5.865746934	6.134066034	5.69E-09
PFDN2	1.194700933	5.018651664	6.131953862	5.75E-09
FYCO1	-1.355168414	5.813002623	-6.117666495	6.19E-09
LRFN4	1.713443565	4.935405325	6.107441464	6.52E-09
CARNS1	-2.406222582	1.019145894	-6.107325074	6.53E-09
KIF20B	1.389211313	5.16679304	6.103529925	6.65E-09
MCM6	1.213493253	5.848002539	6.096986307	6.88E-09
FGD4	-2.055034066	5.252985043	-6.095766534	6.93E-09
MTHFD1L	1.377074717	5.048773922	6.094137078	6.98E-09
SGSM3	-1.064886017	5.65672186	-6.093129442	7.02E-09
DSN1	1.128201729	4.450057735	6.090424798	7.12E-09
CBX7	-1.70906917	4.024866063	-6.09022061	7.13E-09
BCL2L12	1.241657014	3.858950354	6.085299866	7.31E-09
MMP3	4.242257917	3.666650734	6.068965857	7.95E-09
AUH	-1.035252024	3.335083227	-6.066214217	8.06E-09
DBF4	1.404369777	4.594125005	6.061805102	8.25E-09
BRIX1	1.216996251	5.142522604	6.048123417	8.84E-09
AFF3	-3.301413084	0.844420321	-6.044907429	8.99E-09
E2F3	1.042330982	4.662415174	6.035553203	9.43E-09
ETFDH	-1.392446372	4.401532222	-6.035152605	9.45E-09
CHGA	-5.589452804	-0.669578367	-6.032204094	9.59E-09
PLIN4	-3.346277107	1.939493844	-6.027065475	9.85E-09
IL11	2.771619688	1.041833004	6.017320204	1.04E-08
ADAM12	3.244792318	3.990845865	6.017235248	1.04E-08
MT1A	-3.666898103	-0.981559291	-6.008010507	1.09E-08
TNFRSF25	1.623558596	4.037347583	6.004746893	1.10E-08
FNDC5	-2.860371823	-0.103597854	-6.004331327	1.11E-08
OTX1	2.153009754	3.030258556	6.003371152	1.11E-08
MINDY1	-1.641568376	4.440588924	-6.002761851	1.11E-08
APOBEC2	-2.746165573	-1.007343921	-5.999664634	1.13E-08
ASAH2	-2.441538871	0.336520509	-5.999598711	1.13E-08
RFWD3	1.028253283	5.968464577	5.997996736	1.14E-08

NDC1	1.082129739	5.828840077	5.976921741	1.27E-08
ABCC8	-3.962122971	-1.980180419	-5.976297845	1.28E-08
MASTL	1.102542901	4.47405886	5.973454713	1.29E-08
ZNF367	1.492913169	3.980170053	5.967892044	1.33E-08
BLACAT1	3.245336366	1.922738824	5.966796355	1.34E-08
CHAD	-2.953066192	-0.019976881	-5.964024552	1.36E-08
PSCA	-5.781590596	3.17740284	-5.956960353	1.41E-08
ASXL3	-2.961341237	-1.119927701	-5.949110607	1.46E-08
USP53	-1.355412918	5.665731602	-5.937718439	1.55E-08
GEN1	1.064130347	4.835886004	5.937345448	1.55E-08
CENPH	1.511467096	3.128423413	5.935614479	1.57E-08
PRX	-1.411579631	1.801291963	-5.934373498	1.58E-08
ISG15	2.561917578	5.574955745	5.927257854	1.64E-08
GCNT4	-2.691521761	1.864466443	-5.924224296	1.66E-08
CDH24	1.637758997	3.923174063	5.917898176	1.71E-08
CLDN4	3.082512865	7.437619956	5.916191835	1.73E-08
ECRG4	-4.127448379	-1.318112751	-5.899509383	1.88E-08
NOX4	2.400289176	1.116980247	5.894820053	1.93E-08
ALKAL2	-2.708370306	-1.142939483	-5.885059263	2.02E-08
PLA2G7	2.416346817	2.407284507	5.880203052	2.07E-08
SH3BP1	1.923603369	6.062604078	5.876637524	2.11E-08
ATAD2	1.29510954	6.329341787	5.866425269	2.22E-08
H2BC8	2.565580519	0.954125406	5.855658773	2.35E-08
PRIM1	1.284815655	3.134007962	5.853515818	2.37E-08
PACC1	1.178937155	3.158420235	5.846737998	2.45E-08
DTYMK	1.150772297	4.601629131	5.841012517	2.52E-08
NCAM1	-3.617667255	1.169330991	-5.838292463	2.56E-08
MFAP2	2.832122638	3.703293766	5.838043219	2.56E-08
BDH2	-1.265066608	3.372026762	-5.833647313	2.62E-08
CXCL1	3.688691563	4.88916258	5.833562878	2.62E-08
CDH3	3.276668817	6.869137145	5.827646957	2.70E-08
ARHGEF28	-1.549075093	4.841163116	-5.826624952	2.71E-08
DBF4B	1.209696394	3.864844406	5.824711975	2.74E-08
DCLRE1B	1.061916014	3.618654012	5.821380843	2.79E-08
ADGRG2	-2.433812271	0.431321517	-5.816292496	2.86E-08
DLX4	2.542145871	0.871025377	5.81615508	2.86E-08
GREM2	-4.126089667	0.308757952	-5.807835077	2.98E-08
CMTM4	-1.786192033	5.850400172	-5.799997968	3.10E-08
XYLT2	-1.349491983	5.442651128	-5.795206829	3.17E-08
HRH2	-2.696838725	0.1586014	-5.784539336	3.35E-08
BORA	1.232409025	3.124618751	5.756073497	3.86E-08
FZD2	2.292512159	2.655787952	5.752555085	3.92E-08
MIS18A	1.225443607	3.329954801	5.751601524	3.94E-08
CLEC3B	-2.650901539	0.378859552	-5.75097611	3.96E-08
BCKDHB	-1.40080085	4.027772399	-5.741996707	4.14E-08
MELTF	2.506160383	5.162158299	5.739184803	4.19E-08
MCM8	1.210127521	4.950380268	5.73609287	4.26E-08
CCT5	1.20011177	7.721235154	5.733580497	4.31E-08
LRIG1	-1.93366285	6.030203667	-5.728697841	4.42E-08
SERPINH1	1.721911657	7.494270186	5.724854101	4.50E-08
H2AX	1.382878557	6.155616188	5.72281195	4.55E-08
OLFML2B	2.450792777	3.92617289	5.717104995	4.68E-08
PTGDR2	-2.329624458	-0.562812015	-5.717075642	4.68E-08

METTL7A	-2.521660376	4.961493177	-5.708360682	4.88E-08
ACADS	-1.392537111	4.135414302	-5.703664059	5.00E-08
TMEM220	-1.972566713	1.868193712	-5.692769136	5.28E-08
PTPN21	-1.187684241	4.604308956	-5.687304812	5.42E-08
ULBP1	2.859223229	0.560536236	5.680573116	5.60E-08
TONSL	1.360113396	5.463436229	5.679869272	5.62E-08
GLUL	-1.995544394	8.656226248	-5.669134955	5.93E-08
SASS6	1.121110409	3.601827843	5.666381234	6.01E-08
ADGRD1	-3.414829462	1.397277887	-5.663067001	6.11E-08
RANBP1	1.169733448	6.596103549	5.662782108	6.11E-08
ANGPT2	1.721555077	3.726354704	5.661162037	6.16E-08
FAM72C	2.037926644	-0.419953197	5.657375832	6.28E-08
IGIP	-1.29105571	3.088854312	-5.656763015	6.30E-08
PSRC1	1.534303767	2.872763437	5.650553848	6.49E-08
FAM110A	1.390419279	4.546401319	5.649868532	6.51E-08
ILDR1	2.45859393	2.971295137	5.649750677	6.52E-08
TFRC	1.80016033	8.173982985	5.646037405	6.64E-08
GPT2	-2.006884903	5.47547098	-5.64550661	6.66E-08
PRKAB1	-1.076966841	5.323681468	-5.642133417	6.77E-08
NUP107	1.12662903	5.479307215	5.635489323	6.99E-08
SALL4	3.188724189	1.606770172	5.635115175	7.00E-08
SERPINB5	3.79192612	7.092987495	5.634911305	7.01E-08
H2BC11	2.400805594	1.574858924	5.62921644	7.21E-08
ALG3	1.169603055	6.546784417	5.625894344	7.33E-08
COLGALT1	1.078296073	6.942275403	5.61350957	7.78E-08
PLP1	-3.968872204	-1.408174058	-5.610151986	7.91E-08
HS6ST3	-3.784576848	-1.628146293	-5.604985505	8.12E-08
PPIA	1.005961318	9.205571723	5.604162721	8.15E-08
SDS	2.586253076	2.138023883	5.60202289	8.23E-08
COL7A1	3.204757651	6.721219509	5.597632678	8.41E-08
PCCA	-1.45644614	3.608690623	-5.594896042	8.52E-08
PGC	-9.356290607	2.106732903	-5.58508958	8.94E-08
DNMT3B	2.016560525	3.368924347	5.582532684	9.05E-08
MYO18B	-3.612852128	-0.960723497	-5.580685445	9.14E-08
INHBA	3.068569689	5.318750849	5.57234811	9.51E-08
SORCS1	-3.475454985	-1.730863804	-5.571373642	9.56E-08
GPD1L	-1.62209397	4.959861441	-5.569646787	9.64E-08
TUBA1B	1.089481973	8.057399493	5.568824227	9.68E-08
MAGI1	-1.834770197	5.336152919	-5.565817664	9.82E-08
SKP2	1.427539905	5.209293282	5.565203612	9.85E-08
CFD	-2.717242016	3.478149781	-5.563220344	9.95E-08
ARPC1B	1.466926552	7.348800832	5.553971569	1.04E-07
SLC16A7	-2.918056482	3.441696174	-5.546724136	1.08E-07
SINHCAF	1.083162276	6.298562508	5.543029483	1.10E-07
FAP	2.886492732	2.955879514	5.536804113	1.13E-07
UCN2	3.179620241	0.310718401	5.535672184	1.14E-07
MEST	1.90427563	5.367337814	5.53404027	1.15E-07
NEXMIF	-3.667857696	-1.36267952	-5.533781122	1.15E-07
FIGNL1	1.17883335	4.430505161	5.528005794	1.18E-07
MCM5	1.349433064	6.341520407	5.52203197	1.21E-07
CENPQ	1.165521596	2.844203691	5.521591871	1.22E-07
SESN1	-1.401526495	4.374119072	-5.518731671	1.23E-07
DDX11	1.085622239	5.191230137	5.518424447	1.24E-07

VSIG2	-5.134986817	2.001291317	-5.516719006	1.25E-07
FOXP3	1.997364979	2.032745838	5.51398053	1.26E-07
PMAIP1	1.873851427	3.921725567	5.512825814	1.27E-07
IGFALS	-3.376999937	-0.68595938	-5.503480515	1.33E-07
PYCARD	1.522179574	4.737907014	5.502315785	1.34E-07
PAICS	1.11726242	7.088577601	5.494265304	1.39E-07
FAM189A2	-3.430438683	1.151686626	-5.490025383	1.42E-07
ACOT7	1.3032254	5.638997497	5.487053306	1.44E-07
SCG3	-3.488015269	-2.36961093	-5.48563828	1.45E-07
AAMDC	-1.088308124	2.380948524	-5.476145134	1.52E-07
CGNL1	-2.82961839	3.08789889	-5.475081855	1.52E-07
ARHGAP24	-1.794457585	3.189361521	-5.474155968	1.53E-07
ACER2	-2.315796369	2.449773025	-5.473412527	1.54E-07
RTKN	1.065705509	5.736246811	5.472039273	1.55E-07
PLAUR	1.829310272	5.54780149	5.45896946	1.65E-07
RPA3	1.094657647	4.670713733	5.453368916	1.69E-07
VIP	-2.895298972	-2.119071587	-5.451201249	1.71E-07
H2AC8	2.266040629	0.779184819	5.446754003	1.74E-07
SULT2A1	-3.749201869	-2.983638514	-5.444903686	1.76E-07
HMGA1	1.515973063	8.720574431	5.4403169	1.80E-07
BOP1	1.301545299	6.255347133	5.43605556	1.84E-07
MSH2	1.002882955	5.103816092	5.434539952	1.85E-07
ADH1B	-4.907548608	0.398149118	-5.430837932	1.88E-07
NUP37	1.004692589	4.298043536	5.428554801	1.90E-07
TSPYL4	-1.158551684	4.712752959	-5.423950596	1.95E-07
PUS7	1.240649602	5.011428059	5.414612725	2.03E-07
SORBS1	-2.73291864	5.278927465	-5.411941763	2.06E-07
SLC18A2	-1.942321455	-0.08141639	-5.411026647	2.07E-07
TCOF1	1.013653016	6.534113728	5.403660818	2.14E-07
CFAP46	-3.526842381	-0.706275832	-5.395423788	2.23E-07
SCARA5	-4.112880196	0.405810697	-5.395083733	2.23E-07
PTCHD1	-2.909944169	-0.555155254	-5.393108154	2.25E-07
CENPJ	1.186275368	3.873227231	5.38736262	2.32E-07
FBXL13	-2.053913108	0.30448359	-5.386073033	2.33E-07
PKNOX2	-2.313027847	-0.183935185	-5.385214651	2.34E-07
CLN8	-1.058442202	4.10468839	-5.374581212	2.46E-07
RGN	-3.106312991	-0.187555557	-5.373453677	2.48E-07
ATAD5	1.113015661	4.249724815	5.371811244	2.50E-07
RHEBL1	1.397320561	0.04015524	5.370428277	2.51E-07
CCNE1	1.955234038	3.412527108	5.363556086	2.59E-07
HOXA1	2.031011156	2.036327062	5.358329895	2.66E-07
PINK1	-1.122891933	4.384672248	-5.357436882	2.67E-07
PDK4	-3.804873459	4.065155835	-5.356603342	2.68E-07
FXYD4	-3.471731027	-2.13595274	-5.354467485	2.71E-07
BGN	2.326293904	8.10804405	5.354155987	2.71E-07
ZNF778	-1.019169355	3.487246505	-5.348359168	2.79E-07
PSMB3	1.054519138	6.84456649	5.346836032	2.81E-07
ZNF57	-1.144641854	2.85779213	-5.337862092	2.93E-07
MPC1	-1.321198982	4.504432436	-5.337755236	2.93E-07
RELL2	1.627893231	1.382060883	5.334759999	2.97E-07
TYMP	2.206058353	6.899627215	5.334710203	2.97E-07
CLDN1	2.831055858	7.025023562	5.318274268	3.21E-07
CENPO	1.047336527	4.038011174	5.317869502	3.22E-07

PDE1C	-2.246879833	1.031165994	-5.316306247	3.24E-07
ROR1	-2.592529088	2.71725726	-5.314494743	3.27E-07
GPR146	-1.416157274	0.141998294	-5.313103722	3.29E-07
WNT2	2.889018335	1.140446684	5.310914869	3.33E-07
C10orf55	2.43216931	0.16767762	5.310426194	3.34E-07
KIAA1958	-1.511394314	4.078584208	-5.310407418	3.34E-07
SNRPD1	1.030893017	5.911582601	5.309621174	3.35E-07
SLC2A4	-2.797955376	1.097166175	-5.30084259	3.49E-07
GTF2IRD2	-1.349827735	1.130130821	-5.288611159	3.70E-07
FUT9	-5.291932439	-1.122431857	-5.284175995	3.77E-07
PTGER3	-3.162524266	1.635549807	-5.282461521	3.81E-07
PI16	-4.183247293	-0.811148882	-5.281766323	3.82E-07
NEUROD1	-3.206160313	-3.26102481	-5.28137096	3.83E-07
GATA5	-3.903978865	-2.050670831	-5.275237871	3.94E-07
PFDN4	1.012753483	4.056006383	5.274671436	3.95E-07
H1-4	1.817650669	0.566105792	5.274096541	3.96E-07
TAFA2	-1.688143996	-0.334692124	-5.271606973	4.00E-07
WNT3	1.971988633	1.53441789	5.270622046	4.02E-07
SMC4	1.13445251	6.795435165	5.267868449	4.08E-07
S100A3	2.23337819	1.120628591	5.266838272	4.10E-07
CBX2	2.620360313	3.766946568	5.265037752	4.13E-07
LOXL2	1.936657482	5.654254499	5.259201686	4.24E-07
MMP1	3.416743961	6.061346649	5.253935262	4.35E-07
ESPN	2.367911257	4.659716045	5.252407674	4.38E-07
DLEU7	1.488339556	-0.05021254	5.245634775	4.52E-07
C1QTNF6	1.935105909	4.090622582	5.243881483	4.56E-07
CDC25A	1.41667153	3.21256842	5.243527109	4.57E-07
TNXB	-3.377118814	3.751051611	-5.239052756	4.66E-07
CALCOCO1	-1.02084409	5.612603153	-5.23868658	4.67E-07
HMGB2	1.227039087	6.303016284	5.235459608	4.74E-07
FAXDC2	-1.728291064	3.17471285	-5.231459257	4.83E-07
KCNK2	-3.552926875	-1.608322795	-5.231080865	4.84E-07
FAM110D	-1.915182236	-0.018916064	-5.227824907	4.91E-07
FERMT1	1.777229652	6.515619526	5.223050514	5.03E-07
ABCA8	-3.508863814	1.164046561	-5.206154496	5.44E-07
ME3	-2.240439794	2.194332532	-5.201804529	5.55E-07
NFIC	-1.218038214	7.925236702	-5.200790017	5.57E-07
SCTR	-2.958872238	-1.414652423	-5.200780667	5.57E-07
GDF7	-2.43760064	-0.003150017	-5.189850276	5.87E-07
CEP72	1.309301725	3.306836445	5.18352236	6.04E-07
ZNF385B	-3.352447477	-0.664521705	-5.180032653	6.14E-07
TREM1	2.849684792	1.088710829	5.175735298	6.26E-07
HOXD9	2.171538529	1.52150811	5.174188262	6.31E-07
VIPR2	-2.692831317	-1.655699372	-5.17287761	6.35E-07
UPRT	-1.189519849	2.842706469	-5.171709967	6.38E-07
MCM3	1.077546258	6.918301532	5.168912192	6.46E-07
GGCT	1.060800146	5.158547947	5.165596466	6.56E-07
CKLF	1.294121573	2.563399134	5.155651984	6.87E-07
DNAJC28	-1.36291436	-0.164358033	-5.147839407	7.12E-07
ITGA2	1.58827521	6.786078095	5.144732224	7.23E-07
CYFIP2	-2.239728632	4.027976078	-5.141511156	7.34E-07
FAM13A	-1.426819568	4.198918576	-5.139414268	7.41E-07
ERO1B	-2.049010879	3.684841848	-5.131397868	7.69E-07

TFAP2A	2.779326815	5.341094341	5.130477541	7.72E-07
MYZAP	-2.769369447	0.656508791	-5.130302697	7.73E-07
MITF	-1.846344632	3.10284928	-5.129116519	7.77E-07
CMSS1	1.145632022	3.991635239	5.129113651	7.77E-07
DLG2	-2.276132199	0.390412249	-5.127848736	7.81E-07
OAS3	1.503072562	7.553306365	5.120595741	8.08E-07
MMP14	1.466516716	8.014102648	5.112959595	8.37E-07
KLHDC1	-1.536252855	1.144322696	-5.107184262	8.59E-07
THY1	1.910463686	5.684209038	5.103118849	8.75E-07
TXNIP	-1.98312687	8.165599802	-5.102239663	8.79E-07
FCGR3A	2.564242292	3.874811456	5.097463765	8.98E-07
GBGT1	-2.015495238	1.656365952	-5.095811765	9.05E-07
COL1A1	2.61161065	11.0357415	5.092645379	9.18E-07
BID	1.055258194	4.911407175	5.090740171	9.27E-07
RHBDF2	1.196988967	6.065136727	5.090252766	9.29E-07
COL10A1	4.186447821	2.982248478	5.088438432	9.36E-07
C20orf27	1.081623811	4.804417534	5.085560222	9.49E-07
SLC5A12	3.228423497	0.958195761	5.084707722	9.53E-07
NPM3	1.281877013	3.004401966	5.084053965	9.55E-07
SORBS2	-2.784199481	3.797980595	-5.083224671	9.59E-07
YDJC	1.082133186	4.847228225	5.078795749	9.79E-07
PDGFD	-2.196676443	2.483770905	-5.07857898	9.80E-07
KRT80	3.047009774	5.238435424	5.077194033	9.86E-07
KIT	-2.531376385	2.68555282	-5.063454999	1.05E-06
PSAPL1	-4.155676722	-0.831007364	-5.062202013	1.06E-06
GFRA1	-3.386343643	1.572231334	-5.059831041	1.07E-06
TAP2	1.161699906	5.468619979	5.057613792	1.08E-06
PAFAH1B3	1.230429745	4.327713516	5.053062979	1.10E-06
KAT2B	-1.55112626	4.44270189	-5.051141608	1.11E-06
GNB1L	1.132417136	2.690971696	5.045407105	1.14E-06
KLF9	-1.812053199	5.420344506	-5.043134165	1.15E-06
SLC4A11	2.235178154	4.390299207	5.041002539	1.16E-06
CST1	3.922135005	3.314855425	5.029909089	1.22E-06
IKBIP	1.270152764	3.701670116	5.01406944	1.31E-06
PFKP	1.14400432	7.007248002	5.011632145	1.33E-06
MZT1	1.014161344	4.721066191	5.009103171	1.34E-06
COBLL1	-1.43317818	6.144516865	-5.008073062	1.35E-06
ITIH5	-2.293611251	3.698783361	-5.006731888	1.36E-06
STRIP2	2.06277606	2.846864187	5.004996173	1.37E-06
RBPMS2	-2.543655664	2.007710518	-5.002960948	1.38E-06
BCL11B	2.064167737	4.502479247	4.999913906	1.40E-06
STRA6	3.12129597	2.818498744	4.999391057	1.40E-06
SLC5A5	-4.048131565	-0.409663354	-4.989594909	1.47E-06
XPR1	1.182357614	6.390744533	4.98775298	1.48E-06
BICD1	1.110790185	4.270978807	4.984416484	1.50E-06
MMP10	3.609638256	2.256079505	4.9832035	1.51E-06
FMOD	-2.218512445	5.268493919	-4.980654306	1.53E-06
SPINDOC	1.12660923	4.088300827	4.97416989	1.57E-06
XKR4	-2.990668388	-1.676408745	-4.973683244	1.58E-06
RAI2	-2.153568142	2.643418982	-4.97277832	1.58E-06
CCDC149	-1.471763328	3.258556994	-4.969563247	1.61E-06
CACNA2D2	-2.238705451	0.968375973	-4.967414654	1.62E-06
PRKACB	-1.61433883	4.982549009	-4.958134966	1.69E-06

LDHA	1.263665909	9.694494211	4.956496757	1.70E-06
NWD1	-2.903376686	0.356851677	-4.956453493	1.71E-06
TUBA1C	1.216826577	7.797058698	4.955131337	1.72E-06
PAFAH2	-1.160324828	4.335718079	-4.95368869	1.73E-06
FBP2	-2.919332037	-1.090734305	-4.951291185	1.75E-06
RGS11	-2.464938329	0.596559191	-4.951040963	1.75E-06
LIMK1	1.102007714	6.008737654	4.948306341	1.77E-06
PRRX2	2.226361202	2.734075101	4.946324851	1.78E-06
CDHR3	-2.024665519	1.508993127	-4.941291807	1.83E-06
RTN4R	1.539981138	3.127237342	4.939123029	1.84E-06
ADORA2B	2.298944309	3.086267775	4.939063968	1.84E-06
CEP85L	-1.436203958	2.51311624	-4.935327591	1.88E-06
SLC16A3	1.353955812	5.989396884	4.934459076	1.88E-06
PMM1	-1.145917393	3.908323981	-4.921447521	2.00E-06
TNFSF9	2.634583043	1.911214102	4.921165614	2.00E-06
CTPS1	1.112188183	5.269109275	4.920983731	2.00E-06
EPM2A	-1.194010087	2.071890083	-4.920880136	2.00E-06
PCSK2	-3.210288668	-2.01638526	-4.917179118	2.03E-06
ZNF471	-2.368816817	0.444185672	-4.915182984	2.05E-06
ASAP3	-1.531746465	4.182089792	-4.912137766	2.08E-06
CARD14	1.977006817	3.397874003	4.910343303	2.10E-06
SH2D2A	1.460672283	2.702010327	4.907528894	2.12E-06
AP1S1	1.062505099	4.918122565	4.905576495	2.14E-06
AR	-2.257255772	0.762229118	-4.901614676	2.18E-06
TMEM8B	-1.46379023	3.472307036	-4.8967183	2.23E-06
NEURL3	2.654070083	0.422919542	4.893257584	2.26E-06
PSME2	1.008243066	6.490023122	4.892505717	2.27E-06
EPB41L4A	-1.995818213	2.798900998	-4.891369847	2.28E-06
SPP1	3.770315242	5.761544869	4.888692988	2.31E-06
IQANK1	1.920584449	5.523595799	4.887601649	2.32E-06
IL4I1	1.866563732	2.17139738	4.884767491	2.35E-06
ETFBKMT	-1.086313126	1.523850077	-4.878612713	2.42E-06
ADGRF4	3.423624092	2.314117007	4.87729672	2.43E-06
NMI	1.034361488	4.98253026	4.873672031	2.47E-06
OLR1	2.860650049	1.72606086	4.871437385	2.49E-06
PHYHD1	-2.577697229	1.575468466	-4.870157387	2.51E-06
TNS4	3.69596674	7.220297571	4.868093586	2.53E-06
KCNB1	-3.429198669	-0.901918742	-4.860474254	2.62E-06
NTN4	-1.856692638	4.100803425	-4.858544473	2.64E-06
NR2F2	-1.652613977	5.62146542	-4.850997625	2.73E-06
BMP1	1.311530399	5.880873308	4.850160581	2.74E-06
RGS19	1.146392447	3.755534301	4.847359062	2.78E-06
BHLHA15	-2.455064461	-0.54319124	-4.842318318	2.84E-06
NCAPD2	1.115337047	7.378952008	4.839108134	2.88E-06
ENO1	1.086444596	9.792768842	4.835606594	2.92E-06
ZNF852	-1.237817347	1.135115322	-4.835132384	2.93E-06
MT-CO1	-1.369411703	14.0907485	-4.835046038	2.93E-06
AK9	-1.360077102	2.217649252	-4.832477393	2.97E-06
IFITM3	1.393003773	8.343619953	4.831387617	2.98E-06
TACSTD2	2.393950244	8.044215331	4.83049914	2.99E-06
ADAMTSL1	-2.54441233	1.68897542	-4.824350285	3.07E-06
MMP9	2.788129035	4.247312694	4.821194033	3.12E-06
NEGR1	-2.433790739	1.900716177	-4.817149186	3.17E-06

SLC4A2	-1.134941602	6.432131324	-4.815216381	3.20E-06
C7	-4.365704084	1.313914212	-4.813093554	3.23E-06
PRR5L	1.919723569	3.136231631	4.811318794	3.26E-06
TRIB3	1.860960546	4.628061667	4.80941444	3.28E-06
HOXC13	3.533204527	1.207496823	4.805545995	3.34E-06
TSC22D3	-1.69698664	5.950511016	-4.803183548	3.37E-06
ITPR1	-1.571706586	4.270296062	-4.801532688	3.40E-06
TAP1	1.423441455	7.258966314	4.795406209	3.49E-06
GRIK3	-3.178289522	-1.799455254	-4.79459479	3.50E-06
MAGI3	-1.121061382	4.860560201	-4.790354785	3.57E-06
ECHDC2	-1.348816803	5.062298193	-4.789761791	3.58E-06
MFSD2A	1.874019481	4.18541551	4.789126142	3.59E-06
PGF	1.971387696	3.415805897	4.78600459	3.64E-06
THRB	-2.181131263	3.610731494	-4.785275524	3.65E-06
CCDC34	1.318058501	3.654485444	4.783876275	3.67E-06
COL2A1	-3.716505507	-1.028632255	-4.780255432	3.73E-06
EPOP	1.605130741	3.211595369	4.779762942	3.74E-06
PLAC9	-1.833731694	1.089944777	-4.777885702	3.77E-06
C4orf46	1.026813953	3.774885009	4.777280475	3.78E-06
IER5L	1.147044149	4.428160435	4.765130676	3.99E-06
MUCL3	-6.064147305	1.389507995	-4.759411601	4.09E-06
HAPLN3	1.498152289	3.406693815	4.755237861	4.17E-06
NATD1	-1.204113711	3.640797705	-4.746395212	4.33E-06
DIXDC1	-1.448461342	3.697350149	-4.742849058	4.40E-06
ZNF626	-2.382339115	0.725838697	-4.740623541	4.44E-06
MDK	1.740601444	6.823080803	4.738893574	4.47E-06
CTSV	2.209854408	4.326784459	4.7383434	4.48E-06
RCAN2	-1.992035412	3.158036424	-4.736630849	4.52E-06
DENND2B	-1.003672011	6.296362703	-4.730994727	4.63E-06
IDH2	-1.205850173	6.91846638	-4.730936919	4.63E-06
KCNJ16	-4.124291085	-1.438657262	-4.730121825	4.65E-06
GMPR	-2.137946853	1.574836032	-4.728727498	4.68E-06
PAQR4	1.195679805	4.546548507	4.72716502	4.71E-06
FMO5	-2.971001875	2.848784323	-4.716025662	4.94E-06
LAMC2	2.369745603	8.139228746	4.714651301	4.97E-06
CCDC110	-2.174718827	-0.439726337	-4.713965008	4.99E-06
LDHD	-2.300982635	2.420553876	-4.7089246	5.10E-06
ECE2	1.175343973	3.656717195	4.708882967	5.10E-06
MMD	1.27274886	3.91190777	4.701531527	5.26E-06
TCEA3	-1.665486941	4.477300018	-4.700105876	5.30E-06
SPTBN4	-2.208394671	0.324428652	-4.700078364	5.30E-06
APOC1	2.304696339	3.374164772	4.696402179	5.38E-06
COX6B2	2.530370454	0.561590363	4.694329348	5.43E-06
CAPN9	-3.976941312	-0.260539752	-4.688795358	5.56E-06
SOX11	2.387682811	-0.225744431	4.685804277	5.64E-06
HPGD	-3.136652259	3.943975328	-4.682419025	5.72E-06
GAS2L3	1.339608788	3.646488159	4.679386613	5.79E-06
ITGA8	-2.462372219	2.77235547	-4.676234293	5.87E-06
APLP1	-2.743775127	1.894391345	-4.674646991	5.91E-06
LYVE1	-2.317454718	1.144564506	-4.67379397	5.94E-06
ABRACL	1.047052995	4.840032242	4.669309059	6.05E-06
RHOV	3.082554327	4.144785916	4.668472336	6.07E-06
HES4	1.648389246	3.95869157	4.667865682	6.09E-06

HIF3A	-3.25694205	1.652385461	-4.661717976	6.25E-06
RYR2	-2.601468538	1.643499598	-4.66026376	6.29E-06
MAMDC2	-2.954189399	1.272996029	-4.654703516	6.45E-06
PPP1R35	1.026485579	3.957408582	4.6543124	6.46E-06
LRRC2	-2.710010135	-0.824629944	-4.653713262	6.47E-06
ADHFE1	-2.532141914	0.559658388	-4.651311813	6.54E-06
KIAA0513	-1.509171073	4.298735297	-4.649985019	6.58E-06
WDR34	1.006010774	5.676949892	4.643810236	6.75E-06
SYNE1	-1.944913818	4.640225605	-4.643036806	6.78E-06
NFIX	-1.103747963	6.986217565	-4.640433517	6.85E-06
CYB5A	-1.249669946	4.935024767	-4.630471379	7.15E-06
ZP3	1.63341368	2.091416171	4.628404336	7.22E-06
CCL20	3.252825542	3.831022803	4.626835593	7.27E-06
HOMER2	-2.629122584	3.949631488	-4.626537972	7.28E-06
GPRC5C	-2.272186499	5.087155715	-4.625893224	7.30E-06
TNFRSF4	1.47527133	1.081091437	4.624401093	7.34E-06
GPT	-2.273419149	1.34623387	-4.622176465	7.41E-06
HSPB7	-3.048957943	1.664752883	-4.621455854	7.44E-06
GC	-4.139973316	-2.452374453	-4.621429895	7.44E-06
H2BU1	1.967142501	-0.009769219	4.618603096	7.53E-06
PRRG3	-2.228193093	0.758743458	-4.618067234	7.54E-06
STAT1	1.293341344	8.254959457	4.618015742	7.55E-06
LDB3	-2.300632599	0.59778757	-4.617962865	7.55E-06
TESMIN	1.90825234	2.71209153	4.612985256	7.71E-06
FHL1	-2.337941488	4.710072742	-4.6119053	7.75E-06
C22orf23	-1.927891363	0.326395974	-4.610329918	7.80E-06
PDE7B	-1.662006675	1.398875865	-4.610189457	7.80E-06
HIPK2	-1.19107791	7.003866749	-4.608945831	7.85E-06
NUP62CL	1.813389515	1.619663957	4.605589062	7.96E-06
S100A11	1.38303454	9.438824333	4.602826663	8.05E-06
CDH13	1.953266975	4.891743481	4.600641948	8.13E-06
KRT17	4.793866732	8.679048376	4.599627319	8.16E-06
PSMB9	1.709777325	5.300588489	4.596339863	8.28E-06
MARVELD3	1.396267075	4.108740721	4.595451007	8.31E-06
ARHGAP39	1.113809616	4.324452129	4.595343853	8.31E-06
DUSP10	1.408898572	3.584949314	4.592595414	8.41E-06
IFI27L2	1.357377749	3.885298593	4.592084122	8.43E-06
FOXP1	-1.103044027	6.170426972	-4.587353699	8.60E-06
MYOCD	-3.013879682	1.346642389	-4.586149034	8.65E-06
CXCL6	3.015478301	2.296446046	4.585090583	8.69E-06
SNAI3	-1.429715432	-0.025935732	-4.58428788	8.72E-06
GJB3	2.417783851	5.227546358	4.583359073	8.75E-06
CELF4	-2.412258496	-1.649087366	-4.582769975	8.77E-06
MT-CYB	-1.168719974	12.59319362	-4.576688029	9.00E-06
B3GNT5	1.493628551	6.290667576	4.576119792	9.03E-06
SLC2A12	-2.414581561	2.889029272	-4.571627688	9.20E-06
GRIN2D	2.729301722	4.413424335	4.565001982	9.46E-06
APOBEC3B	2.058895584	3.050306307	4.564707282	9.47E-06
C1QTNF7	-2.336755381	-0.550765379	-4.56387353	9.51E-06
SEM1	1.118858243	6.677711335	4.56281781	9.55E-06
AMT	-1.354690824	2.249727329	-4.562788332	9.55E-06
PCDH9	-3.264960534	0.05870579	-4.561289667	9.61E-06
FAM149A	-2.326968355	1.880544905	-4.560800498	9.63E-06

NEDD4L	-1.304447571	6.34251	-4.557385721	9.77E-06
BMX	-1.918480124	-0.561602732	-4.555022429	9.87E-06
ODAM	-4.009839422	-0.991451613	-4.548735079	1.01E-05
RAP1GAP2	-1.45385481	5.569782094	-4.547012863	1.02E-05
RIMS3	-2.105186395	2.361135815	-4.546375476	1.02E-05
SYPL2	-1.94904174	-0.632317951	-4.541660669	1.04E-05
FAM174B	-1.794087108	3.380288919	-4.54066694	1.05E-05
MRPL14	1.009474228	5.042539133	4.539590615	1.05E-05
E2F8	1.297847644	4.023829168	4.537742895	1.06E-05
TREM2	2.253233266	1.82422473	4.537145274	1.06E-05
HSPB6	-2.917706283	2.591624696	-4.534956605	1.07E-05
CCN4	2.257636874	2.337740402	4.53248805	1.09E-05
MROH7	-1.920600937	-0.118245512	-4.529381793	1.10E-05
NUCB2	-1.098281754	5.531933096	-4.527444994	1.11E-05
RPRM	-2.954300391	-1.242583433	-4.525371512	1.12E-05
ANGPTL1	-2.714000707	0.270719219	-4.519900764	1.15E-05
ZC3H6	-1.123539122	3.994535828	-4.518150382	1.15E-05
PARP12	1.028182553	5.81643324	4.517857933	1.16E-05
CRYBG2	2.615144361	4.933138422	4.516052408	1.16E-05
PLLP	-1.883732845	4.391522709	-4.515883876	1.17E-05
GABARAPL1	-1.231411087	5.227185232	-4.513322678	1.18E-05
STC2	2.41248639	3.21711796	4.5124662	1.18E-05
LY6H	-2.30527804	-2.251759354	-4.508970433	1.20E-05
COL4A3	-2.517649314	0.256706152	-4.507747303	1.21E-05
LONRF2	-3.519443208	1.37256667	-4.506279941	1.21E-05
TDRD1	-2.579701793	-2.135391162	-4.504360926	1.22E-05
COL11A1	4.294224944	2.472590982	4.50421261	1.22E-05
SCN7A	-3.334306342	0.039922947	-4.501953358	1.24E-05
PDE2A	-1.713548948	2.39400625	-4.500301217	1.24E-05
CBX1	1.127597867	5.384288707	4.499672568	1.25E-05
HOXA11	2.839361406	0.518619947	4.499441688	1.25E-05
EFNB1	1.492877577	6.774065225	4.496639722	1.26E-05
CCBE1	-2.425019231	-0.102497104	-4.496141814	1.27E-05
DEPTOR	-2.433493691	3.438336419	-4.489340953	1.30E-05
CADM3	-3.185227925	-0.796152281	-4.488038204	1.31E-05
GPRASP1	-1.551403401	1.583615995	-4.487482414	1.31E-05
MROH8	-1.189849505	0.372971524	-4.486164045	1.32E-05
TBC1D9	-1.241049272	4.927861613	-4.483535821	1.33E-05
CLDN16	2.256280337	-0.137752771	4.483031444	1.34E-05
CCDC186	-1.010284679	5.654491732	-4.482780936	1.34E-05
TNS2	-1.32272044	5.62543256	-4.481022932	1.35E-05
PIK3C2G	-3.881050319	1.140881892	-4.478666825	1.36E-05
TNFRSF18	2.405711963	1.96951408	4.476764209	1.37E-05
ABCA13	3.530084391	3.231496737	4.473363268	1.39E-05
RNF180	-1.95233074	0.829992585	-4.471671786	1.40E-05
TMSB10	1.110806884	9.175601611	4.469802177	1.41E-05
SLC39A10	1.050084868	4.958727105	4.469655716	1.41E-05
ACAN	2.320490367	2.115551246	4.468259856	1.42E-05
RAC2	1.539954991	5.117055934	4.466810026	1.43E-05
ZNF662	-2.138032914	0.458332921	-4.464445904	1.45E-05
WDR76	1.007659967	3.622668762	4.461406976	1.46E-05
FAM83H	1.432851084	8.2897501	4.459941865	1.47E-05
CYSTM1	-2.432308107	6.042596453	-4.456217777	1.50E-05

SLC2A1	2.356846213	8.189703509	4.454776187	1.51E-05
SCGN	-2.853004082	-3.104743721	-4.45380911	1.51E-05
CTNND2	-2.649067906	-2.075146991	-4.452279992	1.52E-05
YPEL1	-1.725297405	0.165094029	-4.45012864	1.54E-05
CELF3	-2.692901577	-2.298146693	-4.442520647	1.58E-05
SQLE	1.594498197	6.291538355	4.441957048	1.59E-05
GJB4	2.871097039	1.811360287	4.441504384	1.59E-05
KCNAB1	-1.225681902	1.438907805	-4.441337268	1.59E-05
GPR19	1.638905602	0.354052006	4.437610789	1.62E-05
RPS6KA5	-1.135126384	4.273426017	-4.437476933	1.62E-05
SERPINA5	-3.450722409	0.035742933	-4.436673087	1.62E-05
FABP3	-1.736428884	1.501369564	-4.436100527	1.63E-05
CHI3L1	2.568725471	4.423579701	4.431336629	1.66E-05
WIPF3	-2.234585229	2.314108371	-4.43130269	1.66E-05
TMTC2	-1.135898847	4.32354924	-4.428560915	1.68E-05
MT-ND3	-1.136048115	9.242342245	-4.427646938	1.69E-05
KCNJ11	-2.452701257	0.61655804	-4.423267969	1.72E-05
B4GALNT2	-3.739351517	-0.955048146	-4.412678815	1.79E-05
ZC3HAV1L	1.00750098	3.156723376	4.408558588	1.83E-05
UGT2B15	-3.945430946	-1.45766552	-4.408327412	1.83E-05
KIF24	1.062240156	3.531119522	4.406333206	1.84E-05
DHFR	1.00826138	4.167248389	4.405153626	1.85E-05
ABI3BP	-2.76268384	3.104243777	-4.403606001	1.86E-05
SH3BGR	-1.391409002	0.409630963	-4.399455813	1.90E-05
GGH	1.49559221	5.071278264	4.399025318	1.90E-05
STMN2	-2.546598404	-2.256624276	-4.394362316	1.94E-05
TOM1L2	-1.176706851	6.43661354	-4.391919406	1.96E-05
C11orf54	-1.015542796	4.243243523	-4.391570499	1.96E-05
F10	-2.003292122	0.453381996	-4.391546493	1.96E-05
SERPINB9	1.496630384	4.765829598	4.391346778	1.96E-05
MEX3A	1.922684958	3.100296581	4.38872202	1.98E-05
ATP1B3	1.418088406	7.739882269	4.382654769	2.03E-05
LAMB2	-1.176793676	7.040651874	-4.382201837	2.04E-05
COQ8A	-1.372468641	5.346892898	-4.379603227	2.06E-05
ATP1A2	-3.281133177	0.158935382	-4.378509023	2.07E-05
ADAMTS8	-2.531899121	0.237465971	-4.376454014	2.08E-05
INPP5J	-1.654613393	2.340168698	-4.371420334	2.13E-05
EIF4EBP3	-1.160978922	0.798812124	-4.369539909	2.14E-05
PPFIBP2	-1.324116453	5.083224125	-4.369077592	2.15E-05
PARP9	1.026510407	6.60211329	4.368447754	2.15E-05
ST20	1.028216533	1.295580148	4.368222624	2.16E-05
ZNF695	2.104480932	0.59111312	4.365984409	2.18E-05
BTBD3	-1.140924022	5.526037503	-4.363459452	2.20E-05
ATP8A1	-2.502489804	3.811211525	-4.362496607	2.21E-05
DHRS7	-1.022064557	5.192133028	-4.358369022	2.25E-05
AGTRAP	1.08488492	5.189102667	4.357807594	2.25E-05
OSM	2.019487306	0.083068805	4.354637676	2.28E-05
UNC13B	-1.382443085	5.574557989	-4.353839275	2.29E-05
GAB2	-1.354749452	4.359577837	-4.352749546	2.30E-05
STUM	-3.027347992	0.034457001	-4.352103152	2.30E-05
KCNK3	-2.244686877	0.633491197	-4.348439121	2.34E-05
ZC3H12A	1.435071108	6.164885157	4.346910702	2.35E-05
IL1A	3.553894207	2.055479583	4.343524822	2.39E-05

KYNU	2.397769641	3.700140936	4.34154529	2.41E-05
PMEPA1	1.765552806	6.879157884	4.340092626	2.42E-05
GNGT1	3.493693432	0.483481886	4.339138106	2.43E-05
CD80	1.667895691	-0.10343948	4.336761759	2.45E-05
CHADL	-1.337130602	1.244786336	-4.335629768	2.47E-05
ARRB1	-1.821366945	3.992833631	-4.335321529	2.47E-05
APOE	2.180100787	5.998861685	4.335182626	2.47E-05
RELL1	-1.150960662	3.098082231	-4.331721082	2.51E-05
COL5A2	1.911264048	7.302346761	4.331265505	2.51E-05
XDH	2.476599888	3.982908195	4.331115869	2.51E-05
CTNNA2	-2.260756972	-3.097473969	-4.331065866	2.51E-05
MYORG	-1.634843384	5.462892947	-4.329580541	2.53E-05
CLIC6	-3.238186092	3.184242507	-4.320406264	2.62E-05
PTPRN2	-3.029799022	2.889285927	-4.320237071	2.63E-05
TMOD1	-2.275425662	1.274222228	-4.318981793	2.64E-05
FOXD1	2.469619778	2.175217395	4.31783579	2.65E-05
WNT7B	3.71916095	4.189214756	4.316249025	2.67E-05
SPARC	1.642176823	8.963851903	4.31588266	2.67E-05
SERP2	-1.903563895	-0.396294878	-4.315281077	2.68E-05
VSIG1	-4.037746204	2.356445664	-4.31490719	2.68E-05
PTK7	1.545260261	6.714002269	4.312796338	2.71E-05
ADAMTS14	1.666879227	3.035597609	4.31093029	2.73E-05
COL4A1	1.441155042	8.624400728	4.308852221	2.75E-05
TM7SF2	-1.497147682	3.700181993	-4.307810395	2.76E-05
NCF2	1.724390274	3.215251338	4.306670482	2.78E-05
USP54	-1.15178878	5.795654753	-4.305911999	2.78E-05
NRTN	-1.495136072	1.15273401	-4.305310283	2.79E-05
GARNL3	-1.056589447	2.042068099	-4.297522497	2.88E-05
CHN1	1.303698647	3.514622673	4.29659623	2.89E-05
NRXN1	-2.944473804	-1.045300955	-4.296447349	2.89E-05
SLC26A9	-3.553643985	2.066912751	-4.295480494	2.90E-05
FXYD5	1.367277425	6.512324233	4.291353214	2.95E-05
TCP11L2	-1.213397839	3.650812646	-4.29057539	2.96E-05
SBSPON	-2.074199694	2.158562455	-4.284855319	3.03E-05
FKBP5	-1.765128891	6.282184679	-4.284359375	3.04E-05
PGM5	-2.903967333	1.974825183	-4.282255023	3.07E-05
CHRNA5	1.300383652	1.807544011	4.277480713	3.13E-05
AKR7A3	-3.478792148	2.654646881	-4.275669077	3.15E-05
GCKR	-2.732787008	-0.626750608	-4.27363539	3.17E-05
CSF2	2.497709725	-1.374792938	4.27303511	3.18E-05
FZD4	-1.469483455	3.983330444	-4.271312716	3.20E-05
WDR54	1.160436081	3.227510775	4.270866137	3.21E-05
MADCAM1	-1.784413465	-0.74562038	-4.269006328	3.23E-05
MUC6	-6.012313767	2.320068049	-4.265452707	3.28E-05
CD300LF	1.630441198	0.332501895	4.261720931	3.33E-05
IL23A	1.636148761	0.79263269	4.260742781	3.34E-05
NOTCH3	1.725885909	8.535565832	4.257026644	3.40E-05
SYNPO2	-2.89990706	5.033434467	-4.253791655	3.44E-05
PRDM16	-2.95424621	2.25776028	-4.25365993	3.44E-05
FGG	-3.637162264	-2.796543443	-4.250801644	3.48E-05
SELENBP1	-2.671943475	4.298976705	-4.248995359	3.51E-05
P3H1	1.003067312	5.127799301	4.248733458	3.51E-05
H3C10	1.934932781	1.876160883	4.246980017	3.54E-05

KIAA1549L	2.77796608	3.10011052	4.246685146	3.54E-05
KLHDC8A	-1.913506316	0.394492873	-4.243459183	3.59E-05
OSCAR	1.543396081	1.182429116	4.241290217	3.62E-05
SLC28A3	2.493269601	3.116169829	4.235535582	3.70E-05
ZNF132	-1.099807112	1.413512919	-4.233610151	3.73E-05
FNDC1	2.59403512	3.498702703	4.233494581	3.73E-05
HMGA2	2.975302116	3.33093041	4.229105851	3.80E-05
LMF1	-1.116432399	3.311941267	-4.226482115	3.84E-05
FRRS1L	-2.685592288	-1.252591634	-4.224918448	3.86E-05
MTRNR2L8	-1.291167509	2.310018452	-4.22336626	3.89E-05
MT1H	-2.83414565	0.023021497	-4.221159923	3.92E-05
KCTD17	1.140915522	3.335427971	4.219846633	3.94E-05
CITED2	-1.188535482	5.034290032	-4.216153988	4.00E-05
AGRN	1.109589489	9.018691573	4.214103031	4.04E-05
SLC49A3	-1.474619408	1.915543325	-4.214090095	4.04E-05
SULT1B1	-3.923604614	2.427823734	-4.208173442	4.13E-05
LAMB4	-2.235616228	-1.344867319	-4.207558745	4.14E-05
ZMAT1	-2.327755011	1.227727331	-4.206585663	4.16E-05
MT-ND1	-1.062221606	11.78915838	-4.206002859	4.17E-05
NXPH4	3.190632965	2.55984021	4.203412266	4.21E-05
COL12A1	1.730839085	7.73600226	4.201992214	4.24E-05
IFI6	2.024672556	7.241198144	4.200464367	4.26E-05
GNAO1	-2.086880158	1.90270427	-4.200271002	4.27E-05
MTRNR2L12	-1.296472865	3.087164385	-4.197810755	4.31E-05
MYO1B	1.172209995	7.121136911	4.197415887	4.32E-05
FBXO43	1.580889726	-0.160709185	4.19585597	4.34E-05
RHOU	-2.012433665	3.943501424	-4.195395308	4.35E-05
ABCA12	3.276988103	3.590176217	4.192806039	4.40E-05
B3GNT6	-3.724617207	-0.28004756	-4.192681639	4.40E-05
TMEM25	-1.201066124	3.329196381	-4.188683408	4.47E-05
CHST1	2.116230706	2.571389313	4.18512903	4.53E-05
ADAM33	-2.202734035	1.285394126	-4.18381871	4.56E-05
UNC80	-2.346453904	-2.252499613	-4.181975741	4.59E-05
CLCN2	1.069244186	3.97640147	4.181852423	4.59E-05
P2RY6	1.700992213	2.286142383	4.179384501	4.64E-05
C11orf80	1.05413736	4.074914158	4.178232987	4.66E-05
SYCP3	-1.857609752	-0.477975425	-4.176631688	4.69E-05
GNAZ	-2.013031943	1.922081341	-4.175711472	4.71E-05
PITPNC1	-1.162720814	4.424189711	-4.174961015	4.72E-05
IFITM1	1.613644741	6.103513392	4.174359002	4.73E-05
PLEKHG5	1.186170098	5.462161166	4.173984392	4.74E-05
SLC25A34	-1.404965113	0.588523661	-4.172238949	4.77E-05
CCL3	1.883354581	0.515596446	4.170810211	4.80E-05
RPS6KA6	-2.917182256	-0.49576646	-4.170524962	4.80E-05
TRIP6	1.286018936	5.67091019	4.169440344	4.83E-05
FHIT	-1.576528737	1.558785028	-4.169414149	4.83E-05
LARGE1	-1.408575983	4.266663554	-4.166472191	4.88E-05
FAM83B	1.795157136	4.960709592	4.165153773	4.91E-05
PARP14	1.011754638	7.251912946	4.159173755	5.03E-05
ANKRD13B	1.313619147	3.919233987	4.1493876	5.23E-05
ATP8B1	-1.327260641	6.79254174	-4.149382421	5.23E-05
GAD1	2.437820438	0.97918755	4.143855332	5.34E-05
PARD3B	-1.708051553	3.574185583	-4.142426147	5.37E-05

RAP1GAP	-2.019652975	4.727834523	-4.139344824	5.44E-05
C21orf58	1.09595326	2.880527599	4.134584315	5.54E-05
USP18	1.208076796	2.669471549	4.130962593	5.62E-05
GINS3	1.028676969	3.344490782	4.130251261	5.64E-05
TNFSF10	1.64238982	6.465530184	4.130249446	5.64E-05
FOLR1	-3.576729808	-0.527244966	-4.129111306	5.66E-05
EVA1A	2.290860356	1.103854167	4.128251138	5.68E-05
HERPUD1	-1.0201356	6.534066355	-4.127797799	5.69E-05
MMP13	3.970166607	1.447654179	4.124239997	5.77E-05
ITGB8	1.439212403	6.511368222	4.122831468	5.81E-05
CILP2	2.222073624	0.74889112	4.121768387	5.83E-05
STX1A	1.302058305	3.259479534	4.121579107	5.83E-05
SH2D5	2.693080233	0.515792273	4.120428591	5.86E-05
GSN	-1.199320129	8.555470321	-4.120323101	5.86E-05
FAM83A	3.997390301	4.722813278	4.119395307	5.89E-05
FJX1	1.677738788	3.292297289	4.119364308	5.89E-05
CYP27B1	1.431002349	1.852213186	4.111798532	6.06E-05
SCN3A	-2.105312696	-0.233280755	-4.111104279	6.08E-05
ANKDD1A	-1.064787425	1.937455579	-4.110045245	6.11E-05
TGFBR3	-1.892039082	4.375810706	-4.109947002	6.11E-05
SULF2	1.429228524	7.454948788	4.107190279	6.18E-05
CPNE2	1.08781719	5.0502024	4.103975803	6.25E-05
NFIA	-1.085494496	6.229861542	-4.103099595	6.28E-05
MMRN1	-2.614266961	1.374762855	-4.100260495	6.35E-05
TCEAL2	-2.812299122	-1.577153476	-4.09968198	6.36E-05
CABP1	-1.687607434	-0.498412341	-4.09945153	6.37E-05
DUSP2	1.441481689	2.74821309	4.094367244	6.50E-05
DAAM2	-1.861891114	3.453167901	-4.092969405	6.53E-05
GALNT16	-2.197987174	0.005207961	-4.090492578	6.60E-05
PLEK2	1.765735874	4.643365259	4.090181752	6.60E-05
CGAS	1.544234464	4.034380947	4.087681659	6.67E-05
ZIC5	3.174383397	0.776037179	4.087309322	6.68E-05
GRIN2A	-2.739004353	-0.248692868	-4.08641197	6.70E-05
PRR19	1.284376923	0.398378969	4.082817498	6.80E-05
RAB42	1.290270328	0.79076688	4.082703825	6.80E-05
CPED1	-1.912417189	2.68006934	-4.080202944	6.87E-05
RELN	-2.60881102	-0.257223006	-4.079072927	6.90E-05
RAB3C	-2.746569531	-0.476328223	-4.077168386	6.95E-05
CXCL10	2.715565471	3.387770476	4.074777606	7.01E-05
WLS	-1.009024735	5.903576356	-4.074439439	7.02E-05
NTRK3	-2.600004298	-1.122617129	-4.072180577	7.09E-05
ZNF429	-1.862545979	1.68984127	-4.071897529	7.09E-05
SCARB1	1.183333377	5.585390671	4.069783999	7.15E-05
CHRNA7	-2.192979588	-0.279777722	-4.069731124	7.15E-05
FLVCR2	1.442918683	2.313292236	4.067759772	7.21E-05
LAMB3	1.637320214	8.367132176	4.065645858	7.27E-05
MAOA	-1.791217738	5.870643708	-4.065458048	7.28E-05
GCNT2	-1.913022636	3.277330429	-4.063221254	7.34E-05
CTLA4	1.740469151	0.996153253	4.061556099	7.39E-05
CCN5	-2.807994158	-0.059791322	-4.05976229	7.44E-05
KANK3	-1.275603148	1.78117094	-4.058026912	7.49E-05
TTR	-3.331258027	-2.332545033	-4.056232664	7.54E-05
LIPG	2.029645661	3.876508791	4.055724462	7.56E-05

MOCS1	-1.205424943	2.910575235	-4.054553465	7.59E-05
JAM2	-1.672997709	2.503585031	-4.053840981	7.61E-05
ADAMDEC1	2.141714652	1.60685292	4.051228284	7.69E-05
SIX1	2.321678618	1.947311049	4.049963445	7.73E-05
ARSD	-1.341786516	5.365902184	-4.049348116	7.75E-05
H2BC12	1.469974668	4.253007924	4.047822061	7.79E-05
ENPEP	1.430468754	2.867375987	4.04153448	7.99E-05
PSMD3	1.010919188	7.236488719	4.040887835	8.01E-05
NR3C2	-2.504321008	2.312057535	-4.040695351	8.01E-05
ZNF763	-1.358789716	-0.302683181	-4.039446516	8.05E-05
HPN	-3.623739211	-0.444499574	-4.038717794	8.08E-05
LIMD2	1.267971362	3.585908332	4.036521105	8.14E-05
NPR1	-1.549738845	2.017513891	-4.035906553	8.16E-05
FGA	-3.845074382	-2.016564922	-4.030238031	8.35E-05
SLC4A4	-3.542631286	2.099466634	-4.02756938	8.43E-05
RORC	-3.172678528	1.733154615	-4.026286447	8.48E-05
ZNF425	-1.159671047	1.034016264	-4.021842565	8.62E-05
PNPLA1	-2.163321061	-0.757735133	-4.018044629	8.75E-05
AHCYL2	-1.492016888	5.480142613	-4.016140154	8.82E-05
TRPM2	1.231210973	3.287593978	4.01592944	8.82E-05
HOXB9	3.25987323	1.806479033	4.015295901	8.84E-05
PKP3	1.475092441	7.517304078	4.013701527	8.90E-05
REPS2	-2.04900977	3.737053714	-4.013690911	8.90E-05
TFF2	-5.317521375	0.613021371	-4.012666265	8.93E-05
RTKN2	1.557097714	3.300202678	4.010700306	9.00E-05
HENMT1	1.54836643	2.919249497	4.005523475	9.19E-05
PAK3	-2.588149611	-0.319286468	-4.004423548	9.22E-05
ITM2A	-1.747698069	2.529520225	-4.003705704	9.25E-05
CDNF	-1.193973504	-0.268831222	-4.001524484	9.33E-05
AJUBA	1.365033656	5.343356996	4.001288105	9.34E-05
ITGA9	-1.796726394	3.393478433	-3.995569967	9.55E-05
MAPK15	1.701154397	2.141072771	3.994889707	9.57E-05
TNFSF11	1.885324336	-0.176700226	3.993494176	9.62E-05
PPP1R36	-2.051665531	-0.041360924	-3.992225105	9.67E-05
C18orf54	1.187679288	2.703082842	3.992081537	9.68E-05
REEP1	-2.06076133	1.938741956	-3.991829368	9.68E-05
CD83	1.241125354	3.056554479	3.991314537	9.70E-05
FCER1G	1.62446112	4.503934574	3.989535372	9.77E-05
CDH2	-2.657877485	0.90792793	-3.987646385	9.84E-05
COL24A1	1.946957685	0.951618098	3.985814247	9.91E-05
ZNF493	-1.801898567	2.638830445	-3.985249069	9.93E-05
TWIST1	1.976682365	1.947860038	3.982703683	0.000100313
GALNT12	-1.628616886	4.22238443	-3.980482755	0.000101175
RSPO2	-2.457929964	-1.620913228	-3.976573967	0.000102708
WNK4	-2.70412887	1.715307397	-3.97625074	0.000102836
ELL2	-1.362879262	6.168439766	-3.97517403	0.000103263
GPR176	1.769545401	3.093632378	3.974181266	0.000103658
POGLUT2	1.001096476	2.335838163	3.973041532	0.000104114
DMD	-1.878474204	3.719894963	-3.971041959	0.000104917
PDE4D	-1.120239469	4.477987874	-3.968831219	0.000105813
TNFRSF9	1.564115277	1.507386867	3.967627273	0.000106303
TMPRSS13	2.931128654	2.78619816	3.96592629	0.000107
HPSE	1.252784539	3.773737287	3.965067914	0.000107354

NOSTRIN	-2.094304731	2.716785121	-3.964723433	0.000107496
PPM1K	-1.126730653	3.831685167	-3.963874977	0.000107846
THSD4	-1.7429186	4.886802253	-3.963464684	0.000108016
COL5A3	1.713056391	4.972891946	3.96106564	0.000109016
SP8	2.796782844	-1.57762564	3.955208273	0.000111492
METTL27	1.679240238	1.36513015	3.951125248	0.00011325
CAVIN2	-1.825698111	3.281977817	-3.951082059	0.000113269
TUBB3	1.780692924	0.741845402	3.946600093	0.00011523
DKK2	1.877659121	0.332592875	3.942555433	0.000117026
ACSM3	-2.537075333	1.949269457	-3.940864292	0.000117786
TMEM158	1.780539265	3.982375351	3.940618718	0.000117896
CD86	1.592404385	2.499822122	3.937373677	0.000119367
MT1X	-1.752162031	4.928907453	-3.936770233	0.000119643
CPXM1	1.949771904	2.99054989	3.93621786	0.000119896
LAD1	1.378083529	8.178793882	3.934735521	0.000120576
PHLDA2	1.511796061	5.505074608	3.929206419	0.000123148
GSTA2	-2.816473134	-2.305911398	-3.928000188	0.000123716
DISP1	-1.237173404	2.783825297	-3.926944955	0.000124214
ZNF483	-1.640841776	0.294775974	-3.926860033	0.000124255
PLCE1	-1.412346771	4.300802199	-3.925089065	0.000125096
MOV10L1	1.719276014	-0.75725629	3.923830089	0.000125698
FRY	-1.53103324	3.920676547	-3.921138036	0.000126994
RAET1L	2.929373438	1.634305566	3.917834451	0.000128601
DUSP14	1.168232794	4.587766271	3.917711192	0.000128662
DSCAML1	-2.126910213	-1.470097086	-3.916799163	0.000129109
ALG1L	2.005458779	1.606938315	3.915854074	0.000129574
OAS2	1.528961508	6.772758206	3.914103341	0.00013044
MT1F	-1.690642374	2.81505658	-3.911007061	0.000131985
FSCN1	2.411174263	7.725675684	3.910191109	0.000132395
CSRNP1	-1.109474323	5.080929151	-3.908620173	0.000133188
NOTCH1	1.026934345	7.359980323	3.906256503	0.000134389
KCNQ1	-2.453138579	4.291431532	-3.896478901	0.000139469
PLEKHG4	1.430348812	4.694695192	3.895508154	0.000139983
PLP2	1.081362434	7.736990923	3.895477365	0.00014
KALRN	-1.651575932	4.438143939	-3.894354016	0.000140597
BCL2A1	1.613824157	1.433650736	3.893848536	0.000140867
SAA2	2.987543883	1.337085677	3.893254337	0.000141184
KREMEN2	2.557189555	1.690227374	3.890593589	0.000142614
LPCAT1	1.099316521	5.930289978	3.889441042	0.000143238
SLC2A6	1.286295393	2.45384536	3.889168261	0.000143386
XXYLT1	1.024663236	4.800100867	3.88721143	0.000144452
TOX	-1.963321352	2.324757182	-3.886833453	0.000144659
IGFBP2	-2.181175701	6.412860731	-3.886648732	0.00014476
CCDC74A	1.439653522	1.775920239	3.885612932	0.000145329
PTGES	1.875813074	3.660302245	3.885216412	0.000145547
CRYL1	-1.309592331	3.864575546	-3.883294585	0.000146609
PCED1B	1.30123249	2.617827716	3.883014601	0.000146764
SMKR1	1.750867442	-0.380940189	3.882365431	0.000147125
REEP4	1.043809838	5.544696249	3.880501231	0.000148165
PON2	1.140506913	5.834369886	3.880271293	0.000148294
CNKSR2	-2.524196223	-1.291201757	-3.879819844	0.000148547
IL33	-2.486853778	2.718143528	-3.87951007	0.000148721
OGN	-2.503248346	1.61475918	-3.879505521	0.000148724

RGS1	1.889149446	4.747770686	3.878429568	0.00014933
GCOM1	-2.276424858	-0.140649986	-3.876772236	0.000150267
CCT6B	-1.151391582	0.617523434	-3.876058624	0.000150673
CIB2	1.380297855	2.385451544	3.87166176	0.000153195
PIFO	-2.179509455	-0.326142292	-3.870979506	0.00015359
MASP2	-1.303761699	0.482528956	-3.869200966	0.000154624
EIF4E3	-1.105778353	4.376044546	-3.868575817	0.000154989
ARRDC4	-1.558014869	4.935429786	-3.867226757	0.000155779
PPP1R12B	-1.578339773	6.066773846	-3.867176718	0.000155808
AIFM3	1.395816559	0.544835989	3.864088621	0.000157632
ICOS	1.702763072	0.428666397	3.862716578	0.000158449
SNTB1	-1.738742349	4.099575569	-3.862225028	0.000158743
CCL4L2	1.892042179	0.842596266	3.859349051	0.000160471
GBP1	1.541036416	5.752201469	3.859116099	0.000160612
LAMA2	-1.777241967	3.858580825	-3.858394913	0.000161049
LYRM9	-1.190680914	1.165920449	-3.857273458	0.00016173
RDM1	1.584330686	-0.478970898	3.856445166	0.000162235
ARHGEF9	-1.198305289	3.932960663	-3.853609099	0.000163975
CD276	1.055564954	6.521307541	3.85280284	0.000164473
KIAA0895	-1.170241192	3.662143638	-3.851398019	0.000165343
ACO1	-1.023729589	5.535746901	-3.846253787	0.00016857
SAMD13	-1.908082086	-0.255525661	-3.843387087	0.000170394
DNAH3	1.460321206	1.29951005	3.842591621	0.000170903
MAP6D1	1.007155396	1.688420437	3.842536874	0.000170938
EDA	-1.823546037	1.687018381	-3.841274634	0.00017175
PRAME	4.035201738	1.607546905	3.840837144	0.000172032
CACNB2	-1.565871879	1.574222998	-3.840100882	0.000172508
GPR17	-1.829155906	-0.88159368	-3.839567025	0.000172853
GAS2	-2.256782366	-0.578100589	-3.837704266	0.000174065
H3C8	2.003031523	0.076354376	3.835079325	0.000175786
WDR72	2.757207596	4.615635489	3.831597551	0.000178094
GALNT10	-1.079511148	5.776821942	-3.830663857	0.000178717
TNNT1	3.040189152	1.22566045	3.827003918	0.000181182
SPOCD1	1.622842348	1.58861279	3.82619228	0.000181733
RFX6	-2.690030499	-2.573159932	-3.822045947	0.000184572
BMERB1	-1.226535178	2.802343656	-3.813777868	0.000190361
NFKBIE	1.130721966	3.992641787	3.812821961	0.000191041
SEMA5B	1.567738142	1.093417606	3.812016407	0.000191616
ACKR1	-2.855473603	1.948642076	-3.809763936	0.000193232
CAPG	1.021538518	6.375368519	3.808156038	0.000194394
TDRD9	-1.898546628	-1.264808064	-3.806870588	0.000195327
PAIP2B	-2.105615087	2.391189439	-3.805865885	0.00019606
SPRED3	1.105893202	1.517808637	3.804451397	0.000197096
SYTL3	-1.132759975	2.177615384	-3.802012707	0.000198894
TRMT9B	-2.289999408	0.771629588	-3.801713072	0.000199116
LTBP4	-1.098065002	7.539496205	-3.798447793	0.000201551
DCLK2	-1.40422458	1.078091084	-3.797656382	0.000202145
SATB1	-1.138066981	4.431997069	-3.796989639	0.000202647
WNK3	-1.738657551	0.028545514	-3.79582533	0.000203526
RBM20	-2.000639066	-0.286533978	-3.79212749	0.000206343
RAP2B	1.027096625	6.033299306	3.788970654	0.000208777
SNAPC1	1.128598312	3.274893964	3.787227166	0.000210133
ITGA10	-1.205168116	0.908590213	-3.786317815	0.000210844

PTPRN	-2.545459627	-1.029186107	-3.783368802	0.000213163
WSCD1	-1.882851398	0.120233769	-3.782999737	0.000213455
C6orf223	2.950621851	1.348448571	3.781949188	0.000214289
PKIB	-2.045559199	1.631982866	-3.780505287	0.000215439
NRARP	1.248389346	5.79314681	3.776471309	0.000218684
F2R	1.287599475	5.530741832	3.77080558	0.00022332
AMACR	-1.216776126	2.123084877	-3.770741601	0.000223373
FCGR2A	1.572885675	4.403007359	3.769494611	0.000224406
UPP1	1.465895224	5.005892071	3.763925047	0.000229075
ZNF415	-1.74609887	0.427135464	-3.7587399	0.000233504
NMB	1.370106672	3.065490935	3.751416908	0.000239898
MECOM	-1.955898736	5.507734582	-3.750570871	0.000240647
RAB9B	-1.83305614	-0.01947367	-3.749194043	0.000241872
SAA1	2.843288818	3.380758777	3.747517178	0.00024337
COL27A1	1.356359021	5.697777794	3.746932708	0.000243895
PRSS1	-3.316020336	-1.831827754	-3.746291153	0.000244472
NAT14	1.010912869	2.438050612	3.746070044	0.000244671
CACFD1	-1.071765336	4.239659039	-3.745965283	0.000244765
DMC1	1.446026734	0.038471838	3.744957577	0.000245675
BEND5	-1.771163274	-0.677892672	-3.742783181	0.000247649
TNS1	-1.681335391	7.143392055	-3.742053376	0.000248315
SYTL4	-1.055779935	3.307104612	-3.741722787	0.000248618
IER5	1.19352949	5.419368747	3.741282915	0.00024902
TLCD1	1.028617717	3.295887406	3.739705111	0.00025047
MTARC2	-1.430822132	2.981430994	-3.73925136	0.000250888
VDR	1.116170523	5.627411584	3.738994359	0.000251125
TNFRSF12A	1.302376498	5.471386375	3.73714131	0.000252842
SFN	2.413337014	8.420033303	3.735600341	0.000254278
SOCS2	-1.318059731	3.359264007	-3.733105092	0.00025662
NABP1	1.040654326	4.739049531	3.73284375	0.000256866
ALDH3B2	3.378371308	4.121451996	3.731482271	0.000258154
PPP1R18	1.058215745	5.85874293	3.729674594	0.000259872
RIC3	-2.378391496	-0.110337316	-3.72931446	0.000260216
GJA3	2.763544831	1.364525589	3.729210866	0.000260315
NKD2	1.977107647	2.742451076	3.720281401	0.00026898
SSC4D	1.260242051	0.434753402	3.719394073	0.000269855
JUP	1.561602827	10.72277851	3.716784342	0.000272447
FNIP2	-1.032755966	5.488387343	-3.712737335	0.000276512
MAOB	-2.293880449	3.141102526	-3.706762107	0.000282618
PTGER2	-2.189300475	1.541916903	-3.706221273	0.000283177
AKAP3	-1.348127277	-0.367010341	-3.702945245	0.000286586
ISL1	-2.825142369	-1.13144573	-3.700363195	0.000289299
GABRA3	3.299120279	0.387185165	3.700335414	0.000289329
RUNX3	1.624541463	4.514146116	3.698913357	0.000290834
SPON2	1.210180895	5.208060198	3.695733927	0.000294225
CTH	-1.516985497	1.647563912	-3.695539776	0.000294434
TGFBR2	-1.250226766	6.702335229	-3.693267436	0.000296882
H4C9	1.384577612	3.518525447	3.690603491	0.000299777
IL24	1.825764599	1.295436502	3.68843758	0.000302151
SCUBE1	-2.240744336	1.593362932	-3.688106031	0.000302516
CXCL17	-3.392042083	3.458686207	-3.687645155	0.000303023
CPQ	-1.289250982	3.557217894	-3.684809611	0.000306166
ANXA2	1.039235032	9.821411109	3.683544568	0.000307578

COL1A2	1.787029298	10.07002039	3.682196173	0.00030909
ADAM28	-2.239635117	3.675019748	-3.681545026	0.000309822
POTEF	1.29057026	0.458770489	3.67346835	0.000319046
TENT5C	-1.999608665	3.596123278	-3.673082677	0.000319493
CNTFR	-2.530793514	-0.712599486	-3.672626583	0.000320022
KLHDC7A	-3.108341605	0.190165079	-3.67253236	0.000320131
ITGAX	1.4887981	3.667204337	3.670723249	0.000322239
ADAM8	1.571514451	4.43840433	3.66911632	0.000324123
TRIM29	3.174620845	7.765111053	3.667490723	0.000326039
TFR2	-1.84781283	2.171208085	-3.666220708	0.000327543
ANXA10	-3.899933112	1.9951369	-3.663353937	0.000330962
STEAP1	1.289483898	4.032631076	3.658561886	0.000336754
NAGS	1.336694331	1.915882658	3.657762179	0.000337729
TCF21	-1.571073679	0.988777807	-3.651864033	0.000345009
MAPT	-1.977402412	0.706329298	-3.649791275	0.000347602
SSTR1	-3.868495483	0.65034423	-3.648473142	0.000349261
DUSP9	2.849569562	0.450536066	3.646148278	0.000352204
BTG2	-1.215686464	6.537863198	-3.645632082	0.000352861
MICU3	-1.676637408	0.606282538	-3.645086995	0.000353556
HYAL1	-1.818894274	2.983616152	-3.644026345	0.000354911
MAGIX	-1.384275966	1.808298395	-3.641415672	0.000358269
SYBU	-2.33371185	3.044455487	-3.638674004	0.000361827
SHOX2	2.187899852	0.560093586	3.63722801	0.000363717
SCNN1G	-3.034001425	0.449798322	-3.63683747	0.000364229
QSOX1	-1.0142241	7.917255979	-3.635583924	0.000365877
CHRM3	-1.680693773	3.812993286	-3.634834402	0.000366866
PODNL1	1.662499128	2.610363111	3.634623284	0.000367145
LMOD1	-2.338481857	3.795632965	-3.634220909	0.000367677
RCN3	1.373689996	4.159639274	3.629730737	0.000373667
FAM177B	-1.947074947	0.838100796	-3.627429705	0.000376772
TGFBI	1.85467886	7.345511646	3.62708297	0.000377242
SCD	1.386770823	8.103497696	3.624386314	0.000380916
PPP1R9A	-3.052493434	1.556363259	-3.623485992	0.00038215
RNASE4	-1.718796142	0.444084268	-3.617315713	0.000390709
IFI35	1.016059005	4.484368035	3.617263427	0.000390783
CD68	1.121786527	1.357829709	3.616428062	0.000391956
CYP4B1	-2.835746324	-0.036593919	-3.615887486	0.000392716
ZBED3	-1.333639304	3.982992205	-3.613322828	0.000396345
RAI14	1.059497794	6.206981477	3.611681321	0.000398683
MS4A2	-1.928891981	-0.146324966	-3.611152109	0.00039944
IL32	1.604985295	5.636607526	3.60842693	0.000403359
FAM107B	-1.583906187	5.600004342	-3.605589392	0.000407477
TIMP4	-1.714608132	-0.371280143	-3.60172016	0.000413157
SULF1	1.799050292	6.403651742	3.597803684	0.000418982
GRAMD1C	-1.231584181	3.012282328	-3.59639147	0.000421102
CASQ2	-2.565611575	0.013210859	-3.595454629	0.000422513
HMGN5	-1.609224844	2.173998006	-3.593553465	0.000425392
CNTD2	1.833464875	-0.312947827	3.593009707	0.000426218
SH3BGRL2	-2.297634526	5.399743289	-3.592059255	0.000427667
IFIT3	1.552937106	4.94374223	3.590511135	0.000430036
CDCP1	1.011929428	7.232379025	3.588839863	0.000432607
CNR1	-2.42264727	-0.58980714	-3.588590764	0.000432992
HOXA13	3.124597661	1.8765378	3.586866577	0.000435662

ETV7	1.313439349	3.178587783	3.584604622	0.000439189
RUNX2	1.314170065	3.745722936	3.582233419	0.000442915
FAM47E	-1.839239426	0.0247819	-3.579313231	0.000447544
ODF3B	1.008465115	4.131687484	3.574410406	0.00045542
RYR3	-1.363853786	1.185959233	-3.574136788	0.000455863
TSPAN12	-1.846368086	2.708720293	-3.573217673	0.000457355
SASH1	-1.194986536	5.311349927	-3.572913109	0.000457851
UBE2L6	1.098573203	5.94313165	3.568644386	0.000464848
SOWAHA	-2.620357599	0.982271121	-3.567041581	0.000467502
TMEM238L	-3.345890766	0.01672601	-3.566945784	0.000467661
LBX2	1.170399174	0.186476373	3.56641112	0.000468549
ABCA9	-1.935372987	0.607688023	-3.564154562	0.000472317
ZNF677	-1.580939378	0.934313485	-3.563870201	0.000472794
TMEFF2	-2.025912614	-2.538724303	-3.563607652	0.000473234
PXDN	1.437176576	6.29034616	3.561633545	0.00047656
GSTP1	1.156470283	9.064054383	3.56124481	0.000477217
PLK2	1.146630298	5.244311294	3.560418583	0.000478618
ABCA2	-1.24320256	5.938587313	-3.560171954	0.000479036
ZSCAN18	-1.762993758	2.268750925	-3.559575852	0.00048005
WDR66	2.172886624	3.29019231	3.559541143	0.000480109
CAPN6	-3.25516053	0.907596595	-3.559494538	0.000480188
PLOD3	1.102362583	6.578324836	3.557539747	0.000483527
ZIC2	2.74268251	1.658798081	3.557312047	0.000483917
COL5A1	1.728955144	7.81148207	3.55695873	0.000484523
ANO5	-2.554715484	1.194414286	-3.554938494	0.000488003
SOBP	-1.533184246	2.668894203	-3.552482253	0.000492265
HES2	2.899139129	3.546842758	3.551597495	0.000493809
ADCY6	-1.290163471	4.978068432	-3.550382296	0.000495937
SLC9A3	-2.967364325	3.628965085	-3.548952795	0.000498452
MPZ	-1.428529012	0.095664232	-3.548009021	0.000500118
ABLIM1	-1.243880479	7.911407776	-3.546553303	0.000502699
PAQR5	-1.659410126	3.305255799	-3.545748416	0.000504131
KIAA1211L	-1.641495715	3.428937767	-3.545232634	0.000505051
MYOM1	-1.859451046	1.644684801	-3.544835356	0.000505761
STS	-1.374361641	4.176645777	-3.544692396	0.000506017
COL3A1	1.764455969	10.09337634	3.544482191	0.000506393
LINGO1	1.458625104	2.014601308	3.544379952	0.000506576
SLAIN1	-2.163794268	1.384512796	-3.543001769	0.000509048
SPHK1	1.600768693	4.569503111	3.542120921	0.000510635
MTRNR2L1	-1.262074637	0.529792462	-3.541483085	0.000511786
ADAMTSL3	-2.007485994	1.335458054	-3.539321501	0.000515707
TIMP1	1.257440675	7.58656021	3.538956859	0.000516371
ZNF135	-1.665930649	0.011230335	-3.537754399	0.000518567
LGALS1	1.430383592	6.644383641	3.537141195	0.000519691
SEMA3G	-1.365487941	2.314221457	-3.536502401	0.000520863
C2	1.54130029	3.994410367	3.536255562	0.000521317
ABCA6	-1.81705089	1.112806605	-3.535035398	0.000523565
APLN	1.826217725	2.276320527	3.532810466	0.000527689
NECAB1	-1.857752738	0.381271906	-3.532724006	0.00052785
CA2	-2.370316998	5.314591052	-3.532219786	0.000528789
ZFYVE28	-1.137105805	3.188686882	-3.530783654	0.000531472
WASF3	-1.848203464	2.132822296	-3.529957505	0.000533021
EGFL6	1.355208514	2.999007477	3.529842416	0.000533237

GJB2	2.618559089	6.992906436	3.525076274	0.000542261
C1QTNF3	-1.557739682	1.626564796	-3.524609045	0.000543154
CPEB2	-1.016860933	4.896263107	-3.524342231	0.000543664
JAG2	1.288531308	5.524451559	3.521717111	0.000548708
HMCN2	-2.028756724	2.370083913	-3.520820219	0.000550441
VILL	-2.197800666	3.885849783	-3.519685043	0.000552642
SNED1	-1.338164039	3.75803695	-3.518269827	0.000555398
TNFAIP6	1.716170146	2.121352066	3.511598887	0.000568562
THOC3	1.002099835	3.878759242	3.511359773	0.00056904
NEXN	-1.490116935	3.610636375	-3.511148834	0.000569461
PTGIS	-2.763532769	2.46110804	-3.509400706	0.000572964
HAVCR2	1.418984487	2.580445421	3.508549741	0.000574677
PRRG4	1.231677831	6.301263923	3.507715898	0.00057636
CA4	-2.800772111	-2.000876064	-3.506740318	0.000578334
CEP19	1.328087672	1.271861157	3.506598616	0.000578622
LRP1B	-2.37506039	-1.360405053	-3.50607066	0.000579694
SEMA3B	-1.811224354	4.221730965	-3.503814136	0.000584296
LIPM	-1.6622677	0.327257018	-3.501776735	0.00058848
CTSC	1.180721742	7.426319056	3.500749522	0.000590601
CD36	-1.817531196	3.119329056	-3.500212036	0.000591713
ALB	-2.541753795	-2.090074142	-3.499783002	0.000592602
C5	-1.410365887	2.57955619	-3.499137766	0.000593942
CPLX2	-2.969420236	-1.318543493	-3.497546509	0.000597259
NLRC5	1.128848746	6.270933955	3.497317971	0.000597737
ZNF85	-1.570602064	0.771731126	-3.496162735	0.000600157
APOBEC3F	1.033677774	2.575937209	3.494561918	0.000603526
H2AC11	1.369219559	1.112141471	3.491013899	0.000611057
NPY1R	-2.168020287	-1.486680365	-3.488174085	0.000617148
HOXA9	2.1972581	1.262962881	3.486386022	0.000621013
ABHD11	1.086139357	4.987324036	3.486070506	0.000621697
TMPRSS4	1.722401874	6.68992876	3.484899342	0.000624243
CNN1	-2.263618132	3.96317182	-3.482939727	0.000628525
PLPP3	-1.026972547	5.405509657	-3.482863995	0.000628691
TPO	-1.92588894	-0.985765386	-3.479966889	0.000635074
PAEP	2.234437901	-2.184712336	3.477119433	0.000641406
PFKFB4	1.250624205	3.232897547	3.474514291	0.000647252
HOXD8	1.30974558	1.38527203	3.471007421	0.0006552
NECTIN1	1.690656149	7.77524732	3.469792171	0.000657975
KIF13B	-1.392888817	5.939530405	-3.46875811	0.000660345
CCDC69	-1.390360223	4.109912139	-3.46810099	0.000661856
BAIAP3	-1.34268011	1.564905119	-3.468012759	0.000662059
RDH12	-2.311478413	0.4910513	-3.467680554	0.000662824
RPS6KA2	-1.047357788	4.956154102	-3.46599163	0.000666726
FAM3B	-3.180276525	2.199104375	-3.465702022	0.000667398
TPH1	-1.984094722	-0.301111912	-3.464465685	0.000670271
BMPER	-1.881358504	-0.436543576	-3.463247823	0.000673112
HAP1	2.5032605	1.25145859	3.463080643	0.000673503
ACOX2	-1.445514803	0.926903821	-3.463021779	0.000673641
IFI27	1.556951375	7.629674883	3.460528581	0.000679497
VMO1	1.382842944	0.380990381	3.456074467	0.000690079
CASP5	2.101579371	-1.015630709	3.454098833	0.000694822
CXCL11	2.322060322	1.847588214	3.453161292	0.000697083
PDZRN4	-2.31435461	-1.200328656	-3.452756479	0.000698061

ERVMER34-1	2.00802215	2.69496237	3.452403189	0.000698917
ZNF467	-1.168792966	2.565409909	-3.45060366	0.000703287
AASS	-1.182382232	2.565645048	-3.449895509	0.000705014
FLNC	-2.070119655	4.92318785	-3.449621314	0.000705684
ANK2	-1.951872396	2.26841827	-3.448891513	0.000707469
CFAP221	-2.23423194	-1.402409392	-3.44841434	0.000708639
TP73	2.041786175	3.220805853	3.448021908	0.000709602
ANO2	-1.125021698	-0.258927165	-3.4473112	0.00071135
GKAP1	-1.23211957	1.294711966	-3.44536094	0.000716167
C15orf65	-1.095590421	-0.08614142	-3.444482243	0.000718347
ADAMTS2	1.63763449	5.267062159	3.442027853	0.00072447
FCHO1	1.514952301	2.835757742	3.440919834	0.00072725
PLPP2	1.085018819	5.84608486	3.438999504	0.000732092
MARCKSL1	1.10941495	7.147538838	3.438979979	0.000732141
SARDH	-1.551920201	0.690160191	-3.43884142	0.000732492
FAM151A	-1.069137744	-0.147927436	-3.438282689	0.000733907
PDE4C	-2.185965518	1.026256071	-3.436943104	0.00073731
WIF1	-2.769457969	-2.02752641	-3.436933661	0.000737334
SPATA6	-1.211227554	2.731836467	-3.43615164	0.000739328
ZDHHC15	-1.669028837	-0.845177438	-3.433399119	0.000746386
CHSY3	1.247289524	1.097686744	3.432059206	0.000749844
CACNB4	-1.714421995	0.513300867	-3.431692296	0.000750794
DPF1	1.663626751	1.067061204	3.431597611	0.000751039
ORM2	-2.534333471	-2.342227871	-3.430885748	0.000752885
PDGFRB	1.311075147	6.714481759	3.430811878	0.000753077
PLCL1	-1.334467142	1.814612844	-3.430334328	0.000754318
IL13RA2	1.956102359	-0.411904314	3.43025621	0.000754521
CGB7	1.868611173	-0.821410605	3.428797279	0.000758326
STX19	-1.585386907	1.031478319	-3.428207914	0.000759868
KLHL3	-1.193209914	2.241115772	-3.428029265	0.000760336
MAP6	-1.700434364	0.633837946	-3.427883563	0.000760718
P4HA3	1.25903621	1.000228982	3.426642819	0.000763977
RPL39L	1.760904717	2.818105507	3.425966352	0.00076576
MSR1	1.601600708	3.12932724	3.422171024	0.000775834
STK32B	-1.537138856	0.648091047	-3.420217267	0.000781068
OAS1	1.259661036	6.016683992	3.414799414	0.000795755
CDH19	-2.769565272	-0.469696804	-3.411806093	0.000803981
BVES	-1.823065111	2.065167494	-3.410828294	0.000806685
RNASE2	1.63630276	-1.148635545	3.410549836	0.000807456
FILIP1L	-1.28034939	5.239679973	-3.410223445	0.000808362
MYH11	-2.72796381	7.30629023	-3.409406707	0.000810631
CXCL5	2.927969435	1.938010247	3.4077305	0.000815308
FAM229B	-1.207655984	1.210909377	-3.406065723	0.000819978
C20orf204	1.418059358	0.505654352	3.405793907	0.000820742
RIMS1	-2.104945105	-1.434514886	-3.403929454	0.000826006
IQCN	-1.541170608	1.926791134	-3.403477555	0.000827287
PRICKLE2	-1.336909784	3.417123484	-3.40116337	0.000833874
PLXDC1	1.033911325	3.787458782	3.400439048	0.000835946
MME	1.749380154	2.923849681	3.399192038	0.000839525
FSIP2	-1.952346224	1.45278424	-3.398043613	0.000842833
HMGCS2	-4.438397799	1.387685233	-3.392760115	0.000858209
ZNF568	-1.538419856	0.956011334	-3.392063857	0.000860255
HACD1	-1.400937558	1.306891761	-3.390462656	0.000864977

PLA2G4E	3.29705562	1.832774717	3.389348353	0.000868278
NEB	1.674564101	3.268937589	3.388754859	0.00087004
GIMAP5	-1.382731048	-0.203089222	-3.387603398	0.00087347
ST6GALNAC1	-2.774793566	3.640352503	-3.385913023	0.000878527
B4GALNT3	-1.387611651	5.993225468	-3.385780745	0.000878924
ACTG2	-2.256652688	5.411895189	-3.385410891	0.000880035
FOXD2	1.438778981	1.929204208	3.384683136	0.000882224
SHROOM3	-1.827809722	6.387600532	-3.383945805	0.000884447
CHGB	-2.500844456	-0.937569667	-3.383279775	0.00088646
KRT81	1.829913265	-1.103784743	3.382689293	0.000888248
ZNF470	-1.590910943	0.762607929	-3.381157283	0.000892903
KCNS3	1.708569975	3.249263665	3.376844866	0.000906129
ZC4H2	-1.561762676	1.955170592	-3.374256491	0.000914155
ITGB4	1.14927033	9.478190812	3.372941552	0.000918257
SSTR2	-1.306649574	0.931488789	-3.372193704	0.000920598
CST2	2.011331765	-0.866121825	3.371782066	0.000921889
DMBX1	2.030948962	-1.049577434	3.370780148	0.000925038
IGFBP5	-1.575570015	7.806185441	-3.370681938	0.000925348
IGFBP3	1.463409286	6.968244275	3.3677713	0.000934556
CYP4X1	-2.064860313	2.366940743	-3.367359956	0.000935865
PPP1R13L	1.186647587	6.603058213	3.365798276	0.000940847
MREG	1.084350756	4.027284611	3.365338139	0.00094232
TSHZ2	-1.304562029	4.217024876	-3.363624974	0.000947822
SMIM10L2A	-1.945746513	0.208681352	-3.363236252	0.000949075
SLC9A2	-2.527741784	2.936240106	-3.362370231	0.000951871
ANKRD18B	2.858169181	0.66100502	3.361835944	0.0009536
ARMH4	-1.33219251	1.711662989	-3.360962121	0.000956435
PTPRM	-1.155986469	4.619652988	-3.3595868	0.000960911
EFNA3	1.554003066	3.086635247	3.359215368	0.000962124
LRMDA	-1.356545006	1.170466634	-3.358444092	0.000964646
TNIP3	1.674094954	0.307502468	3.355548194	0.00097417
PNPLA4	-1.076391907	3.332979192	-3.35508503	0.000975702
SLC25A20	-1.046137224	3.094613493	-3.354911662	0.000976276
CXCL12	-1.564562181	3.732707547	-3.35455178	0.000977468
MASP1	-1.767421574	0.293253394	-3.354228759	0.000978539
TTC28	-1.134635674	4.342996051	-3.353908152	0.000979603
ZNF583	-1.284019432	0.076870464	-3.353331086	0.000981522
TTLL7	-1.646656151	2.646050032	-3.350660422	0.000990446
POLR3G	1.136245602	2.552638231	3.34824657	0.000998577
GATA4	-3.601168755	2.575726045	-3.348132921	0.000998961
H4C8	1.417755829	0.803071456	3.346853523	0.001003298
DES	-3.42016354	4.791692134	-3.344416736	0.001011606
AOX1	-2.175363778	1.004679104	-3.344295236	0.001012022
PAQR8	-1.908235715	4.598630328	-3.342374922	0.001018617
TGFA	1.245027308	5.338416845	3.341698166	0.001020951
MNDA	1.350187462	1.740539891	3.338338637	0.00103261
IL1RL1	-1.886408222	0.702712549	-3.338290318	0.001032778
TM4SF19	2.447707646	0.259514732	3.338023189	0.001033711
SP6	1.549339692	2.930742806	3.336135792	0.001040322
ALDH2	-1.077051341	6.620894445	-3.335407547	0.001042883
KANK2	-1.112741926	5.747466547	-3.335365426	0.001043032
PPP1R16B	-1.369060294	2.992223082	-3.334781608	0.00104509
PI3	3.272588365	6.584268233	3.333314315	0.001050278

ACSS1	-1.236457071	5.58308943	-3.332387629	0.001053568
ZNF433	-1.379348744	1.134454057	-3.328914782	0.001065981
BHMT2	-1.585816205	-0.058106905	-3.32883071	0.001066284
SLC28A2	-3.047320408	-0.57992957	-3.328147258	0.001068743
MAPK12	1.339977298	2.810317332	3.325576464	0.001078042
SHE	-1.187876752	1.907733579	-3.324153041	0.001083224
INAVA	1.177358059	6.322917012	3.322604297	0.001088888
LEF1	1.281943621	3.805998828	3.322120841	0.001090661
LILRB4	1.524040218	2.465840099	3.32055298	0.001096432
CHPT1	-1.160678428	4.914907021	-3.319685871	0.001099636
CMTM3	1.130836545	4.071316485	3.319524452	0.001100233
COCH	2.085272845	1.705626947	3.318839732	0.001102771
SYP	-1.38231378	0.599936562	-3.318140221	0.001105368
BAMBI	1.850962291	3.243137756	3.31797731	0.001105974
S100A2	3.888583164	6.491569715	3.317594978	0.001107397
ARMCX4	-1.206817438	2.595497165	-3.316379559	0.001111932
HOXD10	3.119021394	0.982910452	3.315240193	0.001116199
PDE1A	-1.352209784	1.2459876	-3.313511918	0.001122701
NID2	1.41197879	4.236509299	3.313422776	0.001123037
TUBA3C	-1.861644974	-3.527271631	-3.313401713	0.001123116
TLR2	1.18581214	3.905036357	3.313307681	0.001123471
ALDH1L1	-2.422345555	1.468730633	-3.313189085	0.001123919
ABAT	-1.275209842	3.576447669	-3.311320745	0.001130994
WFDC1	-1.350611071	2.282143327	-3.310812799	0.001132925
LRRC26	-2.657499136	-0.202919496	-3.306932445	0.001147776
CDH23	-1.382044611	1.299334646	-3.305532203	0.00115318
STARD8	-1.043714041	2.515093098	-3.302182377	0.001166203
BEX1	-2.357140535	-2.447609089	-3.300316669	0.001173516
SEMA7A	1.110379445	3.079877811	3.300147909	0.00117418
LYN	1.000622817	5.83228645	3.300145714	0.001174188
KLF2	-1.283000874	4.964250221	-3.299780868	0.001175624
SNAI1	1.161454476	2.15748144	3.299645851	0.001176156
CD302	-1.186545393	2.60125885	-3.299419644	0.001177047
ASTN2	-1.542917996	1.840273042	-3.297305375	0.001185409
SPATA18	-1.175364668	2.431618071	-3.296696991	0.001187826
PEG3	-1.506896696	0.230581404	-3.295270938	0.001193508
RPH3AL	-1.490097692	2.951420879	-3.294074115	0.001198296
CASP1	1.244945045	4.519492268	3.293687667	0.001199846
GPBAR1	-1.24688795	-0.080383169	-3.291229539	0.001209749
CYP2C9	-2.461484607	0.027734934	-3.290055213	0.001214506
HRH1	1.041520321	3.773492955	3.289341203	0.001217408
SLC2A5	1.36748338	1.668571635	3.289292272	0.001217607
XK	-2.108858175	3.130141166	-3.289225268	0.001217879
SRD5A1	1.072376463	4.98593488	3.289132998	0.001218255
BMP8A	1.242004092	1.076038026	3.289092133	0.001218421
GALNT15	-1.766672559	1.628820168	-3.288783454	0.001219678
AOC3	-1.603563773	3.829448975	-3.288744461	0.001219837
C17orf107	-1.109758247	1.290387289	-3.288334161	0.001221511
MUC5AC	-4.879346278	3.072711956	-3.283342315	0.001242039
GPX3	-1.690133946	5.394491725	-3.283148291	0.001242844
STARD13	-1.141165771	4.06322568	-3.283100237	0.001243043
SHISA6	-2.261656727	-0.443513897	-3.281561716	0.001249439
IFIT2	1.302847411	3.280060772	3.280816057	0.001252551

S100A10	1.060382739	8.70957449	3.279349056	0.001258692
SLC6A14	2.716156243	3.396834774	3.276124587	0.001272291
TTC6	-2.028941965	0.224879048	-3.275827019	0.001273553
CREG2	2.051228234	1.411963499	3.274557099	0.001278951
ADAMTS13	-1.018470627	1.788844705	-3.27414112	0.001280724
IQGAP2	-2.014369034	4.645906434	-3.272544595	0.001287549
EPHA2	1.16362354	7.243502124	3.270892838	0.001294646
HSPB1	1.714066891	9.643533292	3.269217379	0.001301882
SUCNR1	1.670264317	0.589068656	3.268641846	0.001304376
CALHM6	1.535752424	1.07226362	3.267636023	0.001308746
SAMD9	1.589402397	6.730747422	3.266981528	0.001311597
SDC1	1.427854997	8.80252316	3.265907751	0.001316287
COBL	-2.220063835	4.524013557	-3.265711362	0.001317146
ADCYAP1	-1.917655501	-1.29305259	-3.265668659	0.001317333
FAM83D	1.014684448	5.685956642	3.264498004	0.001322467
PRUNE2	-2.344470116	3.6030651	-3.263757824	0.001325723
NECTIN4	1.682346067	5.106203536	3.261926246	0.001333812
ZNF660	-1.151646556	0.176474474	-3.260191197	0.001341517
NDRG2	-1.195056245	5.115115802	-3.256507884	0.001358012
CYBA	1.024045482	6.664022197	3.254410943	0.001367486
CHRDL1	-3.091730585	0.825726565	-3.253625399	0.001371051
H2BC5	1.237208861	3.64609883	3.253327638	0.001372405
BTC	-1.516263794	1.867667592	-3.252885574	0.001374416
SLC6A16	-1.299843904	-0.047120275	-3.250190902	0.00138674
PLAAT5	-2.281953777	-0.863296782	-3.249939155	0.001387896
UBE2QL1	-1.929421912	0.001839145	-3.247913764	0.001397233
MT1E	-1.747614139	4.900389271	-3.246816901	0.001402314
ALDOB	-3.555929405	0.490209976	-3.24636845	0.001404397
S100A1	-1.191080363	0.975165639	-3.244512401	0.001413046
ZNF439	-1.225095011	1.202565481	-3.243873013	0.001416036
DOC2A	1.75614435	0.063093784	3.24035793	0.001432585
NUDT12	-1.01894016	3.550762046	-3.238768223	0.001440127
ICAM1	1.282535801	5.497577197	3.23855881	0.001441124
LUM	1.431603823	7.668771003	3.238534914	0.001441237
TCTEX1D2	1.179357061	1.797299568	3.238315732	0.001442281
DIRAS1	-2.136922616	0.583378025	-3.23763059	0.001445548
GPC2	1.278217979	-0.104567196	3.235612276	0.001455211
FOSL1	1.623040415	5.298692246	3.23515108	0.001457428
IL2RA	1.382584966	1.872824651	3.233926435	0.001463329
WNT5A	1.531289884	5.706867547	3.229439667	0.001485139
LTB4R	1.718668789	4.713354885	3.228669961	0.00148891
LGI4	-1.200520338	0.777888234	-3.225053124	0.001506753
ADH1C	-3.508709047	1.259206603	-3.220797959	0.001527999
VIT	-2.402098722	-0.679852292	-3.216902491	0.001547693
SIGLEC12	1.737527287	0.092174517	3.216849824	0.001547961
KRT6B	4.37270767	6.120432619	3.216122997	0.001551662
MAPK4	-2.342323489	-0.919396551	-3.215335293	0.001555682
PPP1R3C	-2.112589581	2.11775932	-3.215138139	0.00155669
MFAP4	-1.69641085	5.073227823	-3.214265656	0.001561157
PHYHIP	-1.478407516	0.69143788	-3.211559668	0.001575087
ARHGAP6	-1.513705128	1.829178388	-3.209775612	0.001584335
CLEC7A	1.5468593	2.825215514	3.20649195	0.001601487
B4GALNT1	2.202494625	2.380346046	3.20582744	0.001604979

GNA15	1.866859458	4.704123537	3.205437923	0.001607029
ZKSCAN7	-1.212058865	0.001901713	-3.205194663	0.00160831
FOLH1	1.502221467	1.104685537	3.204052808	0.001614339
LHX2	2.355635369	-0.621970398	3.19957511	0.001638182
SNPH	-1.387581571	2.411661837	-3.199244145	0.001639958
SHC2	-1.220537176	2.761566072	-3.199207325	0.001640155
ADCY5	-1.944966556	2.6138675	-3.19785977	0.001647403
ARNTL2	1.172841332	6.073624192	3.197801149	0.001647719
ZNF469	1.536323552	3.659386441	3.196573358	0.001654349
PYGM	-1.541556244	0.020975063	-3.194449627	0.001665876
HPDL	1.563200206	2.449980227	3.194388407	0.00166621
INSM1	-2.55060113	-1.546367182	-3.192744964	0.001675183
TFF1	-4.549003489	2.033849986	-3.192625647	0.001675836
MAT1A	2.098661931	-0.894535418	3.190950611	0.001685031
NRG4	-1.489414302	0.597483478	-3.190560806	0.001687177
SLC38A11	-2.198201542	0.927802503	-3.187637403	0.001703356
PALM3	-2.387847522	0.398447342	-3.187053751	0.001706604
MROH6	1.439403773	4.955782789	3.185861108	0.001713257
ZNF737	-1.706455674	0.743973431	-3.185328158	0.001716238
FAM89A	1.088586208	3.608157587	3.18393592	0.001724048
PPM1L	-1.185279745	4.67935501	-3.18362055	0.001725822
PRLR	-2.208193387	2.416779174	-3.180139168	0.001745515
CCL26	1.734185154	-1.08156415	3.180019987	0.001746193
CRAT	-1.184950742	5.465335203	-3.178859113	0.001752808
TGFB1	1.213573129	6.529685378	3.178480226	0.001754972
IFIT1	1.57396157	4.126995853	3.175882212	0.001769878
SCNN1B	-2.887129172	1.761725615	-3.174836267	0.001775912
EPSTI1	1.334334071	4.479299953	3.174483287	0.001777953
NOVA1	-1.747426952	0.525992322	-3.171664067	0.001794329
ZNF570	-1.294275614	1.387155932	-3.169843309	0.00180498
PTX3	-1.923933697	-0.369473377	-3.168763805	0.001811323
CLU	-1.712818683	7.171036119	-3.165107514	0.001832959
RASD2	1.413388434	2.614352399	3.164549122	0.001836284
CYP1B1	-1.990957526	2.863796446	-3.163103498	0.001844919
GHR	-1.846655614	1.649045069	-3.157275794	0.001880111
HAGHL	1.090048506	1.887318406	3.15601304	0.001887818
GADD45B	-1.114273127	4.424763809	-3.152055828	0.00191216
RBM47	-1.12819468	7.195971837	-3.151514109	0.001915514
S1PR5	2.494813137	1.798604965	3.151178266	0.001917597
SLCO3A1	1.086230899	5.434471089	3.148397846	0.001934919
LYPD8	2.46136217	-1.761844198	3.147586875	0.001939999
RAPGEF3	-1.079578378	3.434329774	-3.147087699	0.001943132
PLPP4	2.144059353	-0.533813593	3.144185178	0.001961441
KCNH6	-1.929757046	-2.487092778	-3.142425544	0.001972619
LNX1	-1.070206428	3.593047039	-3.141217381	0.001980327
FRMD3	-1.442649644	1.428939783	-3.140910653	0.001982289
DNM1	1.280945675	4.659308683	3.139907946	0.001988713
RALGPS1	-1.244314759	4.220515703	-3.139905973	0.001988726
B3GNT4	1.11814183	0.722467778	3.138618178	0.001997006
FNDC11	1.244062523	-0.24305307	3.138608456	0.001997068
SIRPB1	1.313000012	0.453658457	3.138165655	0.001999923
SFRP5	-2.023856157	-1.703467193	-3.137031875	0.002007248
NAALADL2	-1.415346359	3.029193614	-3.1354849	0.002017284

EPHX2	-1.216104465	3.796509451	-3.134758467	0.002022013
KBTBD12	-2.613259026	-0.072577382	-3.13454252	0.00202342
TNFAIP2	1.195229717	6.82132084	3.134018598	0.002026839
RAB31	1.198866528	6.014428666	3.133315998	0.002031433
RECK	-1.142881291	2.511534859	-3.132245947	0.002038447
P2RX1	-1.669447625	0.537375806	-3.131626811	0.002042515
NR0B2	-3.369442297	-0.475528385	-3.129404153	0.002057183
TRIM36	-1.573732038	1.702508579	-3.128972991	0.00206004
EFCAB6	-1.204265726	0.004999518	-3.127384888	0.002070594
ARL4C	1.257522286	5.399797923	3.127119105	0.002072365
ZNF366	-1.121510321	0.7802475	-3.125339239	0.002084262
OVOL1	1.476820944	4.141961448	3.118773227	0.002128698
PER1	-1.127663611	6.128494288	-3.116912566	0.002141449
KLHL32	-1.590815356	-1.589190029	-3.115140422	0.002153659
FAM20A	-1.381764146	2.508331527	-3.11205092	0.002175099
TYROBP	1.295444769	4.250151251	3.110982361	0.00218256
GPR3	1.176458454	0.955022484	3.110650922	0.002184879
GAL3ST4	1.126490879	2.976274714	3.110575396	0.002185408
PRRX1	1.617602312	4.274526933	3.110339927	0.002187058
PRIMA1	-2.419445942	1.599703709	-3.109749927	0.002191195
PRSS22	1.62555591	4.747885793	3.108492527	0.002200038
ANXA2R	1.003080246	0.960437271	3.104838941	0.002225918
RGL3	-2.219888669	1.245412693	-3.104551652	0.002227965
ZNF665	-1.384961396	-0.432787981	-3.102035795	0.002245965
FZD6	1.054155227	6.366807314	3.102033806	0.00224598
SLAMF8	1.253952671	2.77633316	3.100957254	0.002253723
TDGF1	-2.336721955	-1.793724584	-3.098016575	0.002275001
ZNF790	-1.33307715	0.982046021	-3.097533284	0.002278515
FHL5	-1.380615361	0.207561421	-3.096330814	0.002287282
CELF2	-1.360497419	3.944369614	-3.09529533	0.002294856
TSPOAP1	-1.401657404	2.606666229	-3.094697473	0.002299239
CARD9	1.102010162	1.723981559	3.094315258	0.002302046
CRISPLD1	1.447038525	2.579325215	3.093335204	0.002309256
CACNA1C	-1.197334789	3.427521774	-3.092666608	0.002314187
MAATS1	-2.152246283	-0.365762238	-3.092548283	0.002315061
MEX3B	1.099307874	1.937853194	3.090302316	0.002331703
PGPEP1	-1.186566827	4.439849813	-3.090033517	0.002333702
VWA5A	-1.112948934	4.159615662	-3.088855053	0.002342485
HEPHL1	2.825427829	2.098570129	3.088656744	0.002343966
ARHGAP18	-1.065399726	4.706547076	-3.088255211	0.002346967
MYRF	-2.630026447	5.132441707	-3.087188224	0.00235496
KRT23	2.454975186	2.740800729	3.087015246	0.002356258
SLC43A1	-1.720557727	3.33182069	-3.086947875	0.002356764
G6PD	1.273945912	6.994816572	3.086722996	0.002358453
CD44	1.159817199	9.035510001	3.085627095	0.0023667
S1PR1	-1.219401862	3.769066888	-3.085559728	0.002367207
KCNMB1	-1.543028715	2.370055998	-3.085091197	0.002370742
PPP1R14C	2.595558042	3.437190875	3.084265888	0.00237698
DIRAS3	-1.24857885	0.027887526	-3.083236766	0.00238478
HSPA1B	1.004937155	6.286228926	3.080293869	0.002407214
C20orf194	-1.055034996	4.500327379	-3.079178166	0.00241577
AK4	-1.208868242	5.395705184	-3.079032881	0.002416886
RGS16	1.186452725	3.338166272	3.076913073	0.002433227

KRT86	1.170324256	-0.145454377	3.076848754	0.002433725
TRNP1	-1.730258194	4.650857502	-3.076450849	0.002436804
CBLC	1.177627212	5.320436305	3.076282934	0.002438104
CCL22	1.449574446	1.975476541	3.075800131	0.002441847
KCNG3	1.875930822	-0.991114001	3.075454837	0.002444527
HHIP	-2.182462684	0.645154466	-3.069681136	0.002489746
SLC5A9	-1.804962584	-0.554158253	-3.069219737	0.002493393
TMC7	1.322872672	3.314473372	3.068621729	0.002498127
CTSE	-4.793119093	3.02680171	-3.068538228	0.002498788
AMIGO2	1.429774852	3.916673719	3.067230077	0.002509175
GABRB2	-2.188120579	-1.038735048	-3.06605491	0.002518539
KCNS1	1.752660315	-0.77249203	3.065739502	0.002521058
ZNF844	-1.546760361	2.095183393	-3.065498312	0.002522986
RTL5	-1.317306961	1.37888955	-3.062107285	0.002550234
CD1D	-1.267424884	-0.123057571	-3.060695344	0.002561659
PACSIN1	-1.688031957	-0.297681967	-3.060499608	0.002563247
FAM171A2	1.426890402	1.510436733	3.059457894	0.002571711
KIF3C	1.140372058	3.84059225	3.059043757	0.002575083
COL6A5	-1.635245557	-0.405546948	-3.058931278	0.002576
KRT15	2.898134256	5.898494253	3.058746988	0.002577502
MISP3	-1.413652382	2.773156505	-3.058088409	0.002582878
PERP	1.523995061	9.5145329	3.058021157	0.002583428
GJB5	2.798964648	2.790962535	3.055840739	0.002601303
C12orf56	2.312469539	0.771539725	3.053379422	0.002621618
CLDN18	-4.183150016	3.974194987	-3.05317623	0.002623302
C9orf152	-3.040402715	2.269391517	-3.053139561	0.002623606
NDNF	-1.775946603	0.729200635	-3.050129496	0.002648666
KLK6	2.887488435	3.995857873	3.049426239	0.002654553
PRKAR2B	-1.41291119	2.754623222	-3.046234392	0.002681423
SIAE	-1.070540596	4.6320052	-3.044986245	0.002691998
PLCB4	-1.783237458	3.80873433	-3.041862687	0.002718632
PLEKHN1	1.414224129	3.016250147	3.040945176	0.002726502
GABRB3	-2.621494594	0.736977716	-3.04090969	0.002726807
GBP5	1.82596452	3.613024348	3.040566495	0.002729756
KCNQ4	-1.361286331	1.522922864	-3.03951654	0.002738797
CYP2U1	-1.066921564	2.529610974	-3.039198738	0.002741539
SMAD9	-1.375229226	2.764437454	-3.03864223	0.002746347
GJB7	1.970649187	-0.432085559	3.038289433	0.002749399
ARSI	1.974216789	1.997978889	3.036957196	0.002760951
C9orf24	-1.410484212	-0.969811513	-3.036240194	0.002767187
TMEM151A	-2.157243658	-0.438615356	-3.034999945	0.002778005
H3C4	1.255729012	-0.033730502	3.034224152	0.002784791
CYP26B1	1.536403325	2.709884303	3.031419071	0.002809457
IFI16	1.394262927	6.858929106	3.030549095	0.002817147
NECTIN3	-1.686163473	3.312302323	-3.02924368	0.002828724
GSDMC	2.859861769	1.818224688	3.028300164	0.002837118
AMY2B	-1.287843943	1.151547945	-3.028196842	0.002838038
SERPINA4	-3.151441355	-0.709212323	-3.027070957	0.002848088
GDAP1	1.003342062	3.300328206	3.026273896	0.002855223
S100A6	1.021229105	8.826056521	3.026088223	0.002856887
TRPV4	1.732938736	3.093331386	3.024816257	0.002868312
FER1L6	-2.681597862	1.958327982	-3.024250995	0.002873403
RCOR2	1.216849137	1.900033735	3.01887979	0.002922196

SGCA	-1.827059474	0.033054858	-3.015616725	0.002952209
EVPL	1.521572889	7.295016816	3.01519404	0.002956117
GULP1	-1.350569223	4.235082411	-3.015064623	0.002957315
MT2A	-1.411994986	6.601871441	-3.014500173	0.002962544
BCAS1	-2.825527429	3.077388769	-3.014387525	0.002963588
LGALS9C	-1.898083338	0.884473923	-3.01122798	0.002993023
SYN2	-1.851950389	0.191146625	-3.011125778	0.00299398
KLK8	2.765599179	1.816304579	3.010922876	0.00299588
ANKRD24	-1.238233559	0.183326254	-3.010530615	0.002999556
HCAR2	2.348346616	1.696429728	3.009678859	0.003007553
ST18	-1.70469536	-1.270651381	-3.009216561	0.003011902
LTB4R2	1.313220737	2.606224737	3.007907566	0.003024247
ACTL8	3.048530945	-0.736962111	3.006877006	0.003033999
GK	1.00817549	3.761801501	3.006741192	0.003035286
TBX5	1.521905571	0.786905489	3.006294349	0.003039525
ATP8A2	-1.738299621	-0.116035357	-3.005041102	0.003051442
CDA	1.92427824	3.299968569	3.00456524	0.003055979
FZD8	-1.272533686	3.13999234	-3.003608597	0.003065117
ATP6V0D2	1.41616922	-0.438738654	3.002708481	0.003073739
CILP	-2.330276595	0.888504652	-3.001826922	0.003082204
QPCT	1.47989759	2.214681977	3.001294845	0.003087324
SH3D21	1.171369035	3.233469915	3.000735394	0.003092716
PILRA	1.077582285	1.475046715	2.999385845	0.003105757
MMEL1	-1.570521174	-0.71581532	-2.998527353	0.00311408
ARL11	1.298797985	0.701635761	2.998104076	0.003118191
ARL9	1.368115874	-0.245064481	2.998058921	0.00311863
MUC1	-1.972332169	7.142688037	-2.99795984	0.003119593
IL21R	1.300811093	0.723682717	2.997658136	0.003122527
FADD	1.100615916	4.175971149	2.997007169	0.003128868
FAIM2	-1.8192167	0.272044066	-2.996037138	0.003138337
GRIK5	-1.391171855	0.427519515	-2.995139479	0.003147124
FAM183A	1.612937074	-0.68758559	2.992021024	0.003177825
KAZALD1	-1.251597363	2.203424083	-2.99159728	0.003182018
RIPPLY3	1.947379415	-0.140236125	2.991250255	0.003185455
SOX30	1.577702867	-0.176591993	2.9903098	0.003194788
HBB	-1.873185435	2.437901389	-2.988153396	0.003216284
DNAJC6	-1.518788006	1.444742185	-2.988118714	0.00321663
LRRTM1	-1.766589694	-3.006799996	-2.988045452	0.003217363
CCL3L1	1.454518425	-0.432276851	2.985897456	0.003238913
AZGP1	-2.998830016	2.035980379	-2.985543036	0.003242482
ZNF717	-1.395670966	1.760188678	-2.985395268	0.003243971
ZFP3	-1.46059358	2.603644988	-2.98528904	0.003245041
SLC41A2	-1.656152506	3.714408682	-2.984918166	0.003248782
CD70	1.596570752	-0.097273344	2.983588448	0.003262227
STXBP6	-1.914331837	2.034098331	-2.982713498	0.003271101
ZFP28	-1.407079714	0.67653748	-2.98100436	0.0032885
CD14	1.089851743	4.862452443	2.98042044	0.003294464
TAL1	-1.119528491	0.521626649	-2.979822915	0.003300577
CDKN1C	-1.175349447	3.808363417	-2.977259666	0.003326919
SYTL2	-1.614108373	4.465929075	-2.976971437	0.003329894
UNC5A	-1.763056575	-0.43814077	-2.975721567	0.003342819
RGS7BP	-1.753825284	-1.084458147	-2.975204251	0.003348182
MRVI1	-1.317206954	4.473143904	-2.975138036	0.003348869

FAM222A	-1.092651075	3.039108724	-2.973797125	0.00336281
CRISPLD2	-1.185998882	5.688953986	-2.973654305	0.003364298
SCN2A	-2.137084104	-0.320166562	-2.973564743	0.003365232
GABRQ	2.426574944	-0.020175229	2.973422725	0.003366712
GFRA3	-1.805062593	0.089016567	-2.972149458	0.003380014
CD300A	1.097857296	1.526763642	2.972092843	0.003380606
MSC	1.562197176	2.949791905	2.971725092	0.003384458
PAX9	2.179061951	3.816725979	2.970469486	0.003397638
SYNE3	-1.007612347	2.934073423	-2.970466433	0.00339767
CHST6	1.316879603	1.777623193	2.968483411	0.003418582
MFSD6L	-1.861458522	-0.764145013	-2.967636397	0.00342755
SNRPN	-1.32856707	2.770796002	-2.966580742	0.003438757
HTR1D	2.134998108	0.279955495	2.9655269	0.003449979
ZNF488	1.440062901	2.111977834	2.965074713	0.003454804
MAP7D2	-2.406117326	0.055061973	-2.963435774	0.003472346
PPARGC1A	-1.986549585	2.659104437	-2.962713603	0.003480101
SGSM1	-1.739205231	1.199525034	-2.961720337	0.003490793
OSR2	1.038834355	3.086621378	2.96008455	0.003508468
MPP7	-1.168691409	4.871252883	-2.959224948	0.003517789
JCHAIN	-2.490489993	4.994520143	-2.95726272	0.003539152
FOXL1	1.39891578	1.892853146	2.957154073	0.003540338
VSTM2L	-1.929168411	0.43979618	-2.955792503	0.003555235
EPHB2	1.677526269	5.091661625	2.954863442	0.003565434
TMEM38A	-1.293434634	2.41004878	-2.954134315	0.003573456
CHST15	1.039287359	5.183099957	2.953181657	0.003583963
FIBCD1	2.143532399	0.747001823	2.952509999	0.003591388
ZNF418	-1.519241412	0.004719913	-2.952170885	0.003595142
EPB41L1	-1.04706824	6.582539188	-2.951822856	0.003598998
MYEF2	-1.843035812	1.441673889	-2.951730479	0.003600023
INMT	-1.287125237	1.334414083	-2.949224721	0.003627908
KLHDC7B	2.132865739	2.335849024	2.949034856	0.003630029
AQP9	1.566664775	0.943303137	2.947787004	0.003643997
SLC13A3	-1.599913804	1.236216801	-2.946311826	0.003660574
CCDC74B	1.362700699	0.053272016	2.9461878	0.00366197
IL1RAP	1.248282505	5.180252259	2.944906892	0.003676425
PRKAA2	-2.428300296	1.521594268	-2.944579674	0.003680126
NPDC1	-1.179210559	5.040979718	-2.943885854	0.003687984
ICAM5	1.677482997	0.417668942	2.943711437	0.003689962
TNFAIP3	1.048109443	6.267323817	2.94036166	0.003728139
SELENOP	-1.515153542	5.045981418	-2.940279107	0.003729085
MUC16	2.724353078	1.570684531	2.940087206	0.003731283
STARD10	-1.041655563	6.385198893	-2.939737384	0.003735294
SULT1C2	-3.22695566	1.852169082	-2.939426862	0.003738857
GABRR1	2.021598881	-0.191190149	2.937846674	0.00375704
FCGR1A	1.19639154	0.60755297	2.936688725	0.003770415
SERPINE1	1.691408818	6.749034416	2.935300977	0.003786502
TLCD4	-1.695638578	3.064317605	-2.93481089	0.003792198
DLC1	-1.002933344	4.341654818	-2.93458233	0.003794858
ANO7	-1.301803523	1.745511777	-2.933788771	0.003804104
ENPP4	-1.695058334	3.764971376	-2.932741984	0.003816332
WNT7A	2.618455379	-0.845958786	2.930057261	0.003847858
PDZD4	-1.594624126	0.626647197	-2.926431999	0.003890809
KLB	-1.28102653	-0.770705212	-2.926372282	0.00389152

TMPRSS2	-2.76939575	4.64666683	-2.925826784	0.003898022
CFAP70	-1.083634852	0.776108493	-2.924261849	0.003916729
SLC1A1	-1.596768799	2.944942123	-2.924226805	0.003917149
LYPD1	1.381510985	-0.325181904	2.923041805	0.003931371
KRT16	3.898679603	6.249783434	2.920034856	0.003967672
ECHDC3	-2.09720854	2.049693572	-2.919977621	0.003968365
SLC39A14	-1.076261341	7.032676595	-2.919316386	0.00397639
NCMAP	-2.235767902	0.390022162	-2.91824487	0.003989426
MSX2	1.262357096	2.529849171	2.916627323	0.004009179
SHISA3	-1.880910893	-1.217362735	-2.910959591	0.0040791
ASPN	1.402629151	3.831551921	2.90936444	0.004098979
KCNMA1	-1.930605321	3.302898265	-2.909097511	0.004102314
FGF14	-1.77619778	-0.639440159	-2.908602674	0.004108504
TRHDE	-1.986907155	-0.940586703	-2.907442365	0.00412305
ZNF528	-1.383209731	2.269733673	-2.90420286	0.004163913
TBX15	1.454622862	0.238024185	2.902178606	0.004189635
SLC12A2	-1.433570178	6.457113606	-2.901295564	0.004200901
AGTR1	-1.852512483	-1.546091349	-2.900585528	0.00420998
LAPTM5	1.091290726	6.189631849	2.896951024	0.004256735
ALPK3	-1.196074342	3.509779401	-2.895957391	0.004269599
FGF13	-1.667249661	1.064773285	-2.89499036	0.004282154
NLGN1	-1.878820125	-0.644249642	-2.892823415	0.004310408
S100A7	4.261704661	4.112270229	2.891912095	0.004322342
PWWP3B	-2.046301335	-1.862687526	-2.890523863	0.004340579
APOH	-2.026364321	-2.636281116	-2.890346682	0.004342911
NBEA	-1.623894781	1.982486371	-2.890308828	0.00434341
RIBC2	1.485050198	0.614154284	2.889863906	0.004349273
ADGRE2	1.09537398	2.610717585	2.887248771	0.004383881
PTGDS	-1.611031583	2.990861914	-2.885969164	0.004400907
DNAAF3	1.168318511	-0.143079652	2.883986116	0.004427412
COL6A3	1.334399832	8.707351944	2.882864668	0.004442466
NKX2-2	-2.102275159	-3.155795242	-2.880145707	0.004479157
KLC3	2.226180796	1.872121331	2.87918653	0.004492167
LOX	1.250001117	5.093497846	2.878137262	0.004506438
GPLD1	-1.150768447	0.512404032	-2.877677351	0.004512707
PAK5	-2.121140603	-2.197445607	-2.876606705	0.00452733
GATA6	-2.131221454	4.193923192	-2.874875035	0.004551074
EEPD1	-1.106364567	3.94322385	-2.874373055	0.004557978
RASSF6	-1.52866348	4.257229155	-2.871591225	0.004596412
HCAR3	1.966520824	-0.26687091	2.869461324	0.004626039
LMO3	-1.399892883	0.815242103	-2.865249884	0.004685134
HCAR1	-2.229913222	-0.137480718	-2.864530682	0.004695294
ADAMTS20	2.368789072	-1.183684541	2.864321726	0.00469825
TEK	-1.080337271	2.521061275	-2.860514104	0.00475241
SLC2A10	-1.161990928	4.20095022	-2.859388231	0.004768533
BMP3	-2.287331999	-1.232654716	-2.858717949	0.004778155
ALDH1A1	-2.288119293	6.112979623	-2.858463826	0.004781808
DNALI1	-1.732845249	1.236714319	-2.85783323	0.004790883
PTPRT	-2.063159977	-1.274239986	-2.8552267	0.004828563
ZCCHC24	-1.021807712	4.090644876	-2.851281095	0.004886114
PGGHG	1.153314685	4.695880998	2.850808724	0.004893046
C2CD4B	-2.034653394	1.715587251	-2.850680899	0.004894923
SNORC	1.224106046	1.223480664	2.84927631	0.004915595

NTF4	1.884293815	-0.409354522	2.849061941	0.004918757
TLE2	-1.161750935	3.289062965	-2.847106098	0.004947692
VWA2	-1.822433289	2.54178268	-2.84710376	0.004947727
CADPS2	-1.223479463	4.28384906	-2.84554728	0.004970864
ASCL2	1.824707548	2.522713431	2.84466785	0.004983981
ZNF69	-1.719010939	1.304874155	-2.844206972	0.004990868
APOD	-2.001424081	3.210086327	-2.843088274	0.00500762
ABHD2	-1.025149381	7.91424409	-2.841998731	0.005023985
SOD3	-1.369929959	4.852455963	-2.84198546	0.005024184
CYBRD1	-1.242116127	5.827855233	-2.841507878	0.005031373
SEMA3E	-2.242147569	0.553425339	-2.84134483	0.005033829
RNASE10	1.99203117	-1.927103233	2.840274644	0.00504998
TGFB3	-1.083168091	3.387116787	-2.839670279	0.005059121
PIK3AP1	1.250048467	4.274959836	2.838477962	0.0050772
ANKRD22	-1.31516229	4.158077703	-2.83766224	0.005089602
SLITRK3	-2.024357694	-1.737825447	-2.835378371	0.005124474
DMRTA1	-2.014159896	0.37541582	-2.833464016	0.005153872
HOXB13	2.397631645	0.967430296	2.826691507	0.00525911
TMEM47	-1.161578936	3.801564608	-2.82543875	0.00527879
ATP1B2	-1.166633563	0.616385	-2.824336636	0.005296159
HOXD11	2.89110608	-0.513449555	2.824183134	0.005298583
ZNF682	-1.455143823	0.289704783	-2.823954815	0.005302189
RALGAPA2	-1.113190879	6.183942748	-2.82363249	0.005307284
PLN	-1.969371816	1.542101119	-2.820608448	0.005355301
FAM110B	-1.311947064	1.049651529	-2.8195573	0.005372084
CCL18	1.862634613	2.890054941	2.819359069	0.005375255
RGS9	-1.126312257	-0.073350568	-2.818616958	0.005387139
ZC3H12B	-1.303329025	1.419972241	-2.81826073	0.005392852
SMPD3	-1.914178388	3.407847391	-2.815951213	0.005430026
SYNGR1	-1.526567467	3.162878673	-2.811373453	0.005504399
TMEM154	1.592874865	5.073156518	2.809847095	0.005529403
ACKR3	1.523215145	4.934569537	2.806948922	0.005577163
GNLY	1.610967901	1.922957669	2.806195025	0.005589648
IGFL2	1.967485022	-0.702019952	2.803588067	0.005633018
FOXL2	2.333670643	-0.975095498	2.802741204	0.005647173
ENKUR	1.307333857	-0.267031881	2.802698088	0.005647894
ZNF221	-1.030291712	0.367020012	-2.801071247	0.005675179
WDR17	-1.830474248	-0.894050642	-2.800988003	0.005676578
FADS2	1.393197326	4.829447032	2.800383201	0.005686755
TNF	1.46291497	0.213277	2.800022422	0.005692833
ST8SIA2	1.649770708	-1.112187595	2.799802078	0.005696548
VLDLR	-1.322465279	3.818065574	-2.797910383	0.005728535
TRIM2	-1.024916304	6.786191242	-2.797874258	0.005729147
CEMIP	1.533911773	5.163162816	2.79760912	0.005733644
ADAMTS15	-1.550732554	2.460694288	-2.794923633	0.005779371
IFI44	1.191243321	4.887978271	2.793711876	0.005800112
DSG3	3.833078391	6.193393157	2.793546429	0.005802949
S100A16	1.131792966	8.038483154	2.791775293	0.005833401
PTGS2	1.565262797	4.027233322	2.790146322	0.005861536
TF	-2.304531757	0.20994248	-2.784639074	0.005957573
CD9	1.014502938	8.267326631	2.784581074	0.005958592
ZNF382	-1.038502146	0.439703347	-2.784354115	0.005962581
CAPN13	-2.36379671	1.32494969	-2.783569008	0.005976399

PGLYRP4	2.564010006	0.589105592	2.782111088	0.006002134
RAB17	-1.984559962	2.461712256	-2.780976661	0.006022229
ZMYND12	-1.162689162	-0.188069288	-2.780495075	0.006030778
SLC52A1	1.683895557	1.204686001	2.780346759	0.006033413
PRODH	1.735277144	1.747572905	2.780265088	0.006034865
NAT8L	-1.638085555	-0.359046683	-2.777429538	0.006085457
SERPINE2	1.51443962	5.130706711	2.776643987	0.00609954
KIF26B	1.168911586	4.621762065	2.77636221	0.0061046
HSPA6	1.710259729	2.514451783	2.774515994	0.006137841
RAB38	1.847268118	3.302200487	2.774495327	0.006138214
SVIP	-1.455415902	3.876051714	-2.772848469	0.006168007
SEZ6	-1.252900165	-1.296117366	-2.771559902	0.006191409
ZNF420	-1.112849162	1.699684028	-2.770114175	0.006217762
ZNF347	-1.338252181	1.917530894	-2.768566812	0.00624608
SLC25A27	-1.226474141	1.692859065	-2.768040268	0.006255742
MAL	-3.345247319	1.847865845	-2.766579294	0.006282625
GPRC5B	-1.484841687	4.198731053	-2.766037872	0.006292613
BSN	-1.138236652	0.568359627	-2.765752341	0.006297887
FPR3	1.17471763	3.113137047	2.765692541	0.006298992
CA3	-1.602335591	-0.260803487	-2.763581102	0.006338121
TRPV6	-2.016480577	0.49357169	-2.76183869	0.006370576
ID4	-1.223072191	3.102691688	-2.761564338	0.0063757
MKRN3	2.238828602	0.289927851	2.761131563	0.006383791
ZNF571	-1.099910035	1.213279341	-2.760131381	0.006402525
CCL21	-2.272011264	2.535060043	-2.757014704	0.006461221
GNAL	-1.096227585	3.109172067	-2.756197886	0.006476685
PTPRB	-1.079530166	4.459405071	-2.755301199	0.006493699
ADCY2	-1.632913009	0.03741487	-2.754881463	0.006501677
DLX5	2.440983821	1.060178585	2.75336001	0.006530672
FOXF1	-1.11368321	3.273514918	-2.752728682	0.006542737
INHBE	1.304027414	-1.371554099	2.752674496	0.006543774
OLFM2	1.472844487	3.266829924	2.752444527	0.006548175
KIF5C	-1.203145572	0.564451081	-2.751478261	0.006566694
GRAMD1B	-1.819759406	2.89092335	-2.751255816	0.006570965
ADCY1	-1.196428676	1.827251646	-2.749578881	0.006603237
SLC16A10	-1.268771864	1.989404177	-2.749042217	0.006613596
RETREG1	-1.496536058	3.656927593	-2.749040541	0.006613628
LRRN4CL	-1.062727639	0.657690822	-2.74757597	0.006641972
GIMAP7	-1.038750642	1.87499393	-2.747512338	0.006643206
SMIM10L2B	-1.158005645	-0.218064591	-2.745678347	0.00667886
MYOM2	-1.393797167	-0.003335592	-2.743123794	0.006728812
FZD10	2.160660651	3.029520992	2.740269521	0.006785025
NFE2L3	1.064491242	5.458708537	2.739158295	0.006807025
ATP1B1	-1.069009914	8.248289532	-2.738879169	0.006812561
PYGL	1.698033256	5.300882931	2.737754524	0.006834908
RTN4RL2	1.010564077	1.730454646	2.737410533	0.006841757
PID1	-1.296965549	2.214633898	-2.737331958	0.006843322
LIMS2	-1.112443834	3.802777154	-2.736702084	0.006855882
A4GNT	-2.223594576	-1.632317043	-2.732857361	0.006932996
PTGS1	-1.172154298	4.577779123	-2.732649247	0.006937193
RNLS	-1.586873691	1.094846251	-2.731763024	0.006955089
AATK	-1.380094521	3.043992859	-2.731690282	0.006956559
DLK2	1.162437439	2.26843514	2.731308955	0.006964274

IL36G	3.030472718	0.776309229	2.729986592	0.006991087
CFAP74	1.542630667	-1.071586918	2.72966824	0.006997556
SLCO1A2	2.366762555	0.008102942	2.729127478	0.007008557
ASCL1	-1.768030806	-2.472162893	-2.727536926	0.007041006
SGK2	-2.303735895	1.85830513	-2.725815272	0.007076282
PCSK1	-1.650454786	0.686778226	-2.725619499	0.007080303
PDPN	1.356751917	4.94607565	2.725268999	0.007087508
IGF2BP2	1.036190834	6.136792076	2.7240827	0.007111943
BLK	-1.957155221	-0.999175705	-2.722066528	0.007153647
FGB	-2.700241736	-1.969352818	-2.720286189	0.007190656
CXCL9	1.923519602	4.266649307	2.719169162	0.007213965
EREG	2.277359752	2.909724353	2.717760127	0.007243465
ZNF569	-1.145003518	0.789239276	-2.717592721	0.007246977
C6orf141	1.486042421	2.488016232	2.717447406	0.007250027
SIGLEC10	1.089101061	2.032712254	2.715354902	0.007294073
F2RL2	1.467479953	3.126088005	2.713688093	0.007329333
CLDN5	-1.127355628	3.07022317	-2.712285787	0.007359116
CD177	2.21708901	1.105614622	2.711720675	0.007371149
CA9	-2.417933007	2.926984867	-2.711008032	0.007386349
GALNT17	-1.357871557	0.617588729	-2.710367224	0.007400041
PDE1B	-1.124720365	0.798562563	-2.709412667	0.00742048
CORO2B	-1.182670185	0.498606046	-2.708981046	0.007429738
AKAP6	-1.175864161	2.342787667	-2.70797967	0.007451258
DLX2	1.875309073	-0.667727838	2.707751765	0.007456164
ABCC6	-1.209835007	2.170064673	-2.707111368	0.007469964
C1QC	1.225346919	5.720922658	2.706015673	0.00749363
VSNL1	2.253606768	3.942984794	2.705393441	0.007507099
ENPP1	-1.258143595	2.806713824	-2.703862728	0.007540327
PELI2	-1.168068379	4.121816321	-2.702578892	0.007568299
SLC8A2	-1.40207916	-0.865524936	-2.701355694	0.007595037
ENOX1	-1.039349853	0.471869438	-2.700474452	0.007614353
S100A7A	3.445885917	0.967669712	2.694056003	0.007756385
GPAT3	-1.202412378	2.920343311	-2.694026685	0.007757039
ZNF114	1.633193312	0.166960321	2.69352623	0.007768214
IFFO2	1.147381652	5.836276357	2.693204146	0.007775414
GRHL3	2.17951674	3.685689924	2.69026311	0.007841438
IL22RA1	1.176148706	4.157628132	2.689008032	0.007869767
RAB37	-1.264389946	0.581329518	-2.688840163	0.007873563
RSAD2	1.318507697	3.920308407	2.688813512	0.007874166
ZFHX2	-1.027226424	1.695546471	-2.68860419	0.007878902
RNF152	-1.113714976	4.357143387	-2.688330668	0.007885095
ARHGAP44	-1.287070839	2.915465823	-2.686105383	0.00793564
SLFN11	1.05427592	4.502605542	2.685837255	0.007941749
SLIT3	-1.281707852	4.612477151	-2.68476953	0.007966122
FA2H	-2.002797015	3.875159885	-2.684476125	0.007972832
DTNA	-1.537357444	1.920986567	-2.684010259	0.007983495
GRIN2B	-1.572035453	-1.639903211	-2.683761159	0.007989202
GADD45G	-1.031084345	1.811991276	-2.682152714	0.008026142
RAB27B	-1.172433368	5.385010052	-2.681347978	0.008044681
SNAP25	-1.567999986	-0.461190858	-2.680406138	0.008066429
PRELP	-1.736922235	3.442770816	-2.67881276	0.008103341
REG1A	-4.411082273	2.148495798	-2.677542383	0.00813288
CSAG3	2.091473797	-2.328751119	2.67598723	0.008169172

TMEM40	2.751772293	1.892262569	2.675424508	0.00818234
HTR2C	2.195700671	-1.59660235	2.674247209	0.008209952
H2BC18	1.044463922	1.06734428	2.671263852	0.0082803
CTNNA3	-1.791035331	-1.348600854	-2.667999898	0.008357887
ODC1	1.121426709	7.526745143	2.664973852	0.008430404
APOA1	-2.072480835	-1.193233809	-2.663368719	0.0084691
CCDC9B	-1.111034944	3.516272578	-2.663305512	0.008470627
SLC14A1	-1.468179497	-0.449914709	-2.662827155	0.008482192
RAB26	-1.131421366	0.805896494	-2.660808155	0.00853116
THSD1	1.029023087	2.556346713	2.658166217	0.008595623
TMEM171	-1.343564011	2.481102745	-2.657385216	0.008614763
GAL3ST2	2.004251342	-0.24979455	2.657246119	0.008618176
MESP2	1.450437158	-1.052966145	2.656729976	0.008630851
SLC11A1	1.000586595	3.025831766	2.656694384	0.008631726
KIAA1324	-2.331281747	4.872791849	-2.65521939	0.008668043
SLCO2A1	-1.147365793	5.087354741	-2.650590735	0.008782905
CD109	1.591585815	5.715149357	2.650332896	0.008789344
CTSF	-1.212261762	3.401984044	-2.649884123	0.00880056
CNGA1	-1.15469629	1.492157626	-2.648403589	0.008837656
HCK	1.008885635	3.207925657	2.64835075	0.008838983
NFATC2	-1.202558624	4.509253074	-2.648204816	0.008842647
STK33	-1.45887474	0.505449251	-2.647535226	0.008859479
TACR1	-1.43664424	-0.682327566	-2.646504752	0.008885439
AADAC	-2.044015744	1.033776957	-2.644938563	0.008925026
PREX2	-1.107323986	2.674180367	-2.644720993	0.008930538
PKP1	3.599504703	6.761999096	2.644505518	0.008936
LRRC31	-2.548812878	-0.326003613	-2.644448059	0.008937457
VCAN	1.32867261	6.664523094	2.643293164	0.008966786
LAMA1	2.039294503	1.709492175	2.642903242	0.008976708
SMOC2	-1.462543257	3.751159222	-2.64161028	0.00900968
COL4A4	-1.664424989	1.75654965	-2.641487038	0.009012828
C14orf132	-1.262143832	3.34116235	-2.641286833	0.009017945
COL14A1	-1.300042971	4.44094925	-2.637450009	0.009116512
PERM1	1.165908801	1.348894115	2.634241158	0.00919969
LIF	1.326016325	3.428125706	2.633171573	0.009227567
CYP1A1	-1.918838746	-2.598326052	-2.632927701	0.009233933
GPR87	2.957898298	2.151518215	2.628153093	0.009359382
HK3	1.190414496	0.878797972	2.627422199	0.00937872
ANXA8	2.812655487	2.506134023	2.627128194	0.009386509
APBB1	-1.023185156	2.935737302	-2.626998504	0.009389947
DLX6	2.407701513	-0.60049482	2.625113064	0.009440052
CLDN6	2.023837583	-1.816333183	2.624226949	0.009463684
SYNM	-1.661521478	4.587458462	-2.623504094	0.009483001
OSR1	-1.202324371	1.323250557	-2.62281487	0.009501453
EPN3	-1.069263754	5.377789246	-2.622079885	0.009521165
SERPINB2	2.836754901	2.563187252	2.62091211	0.00955256
GSTA1	-2.956406952	1.081965781	-2.61927281	0.009596788
TMEM170B	-1.039844292	2.057564766	-2.619262365	0.009597071
PDZRN3	-1.310292993	3.601789312	-2.61857715	0.009615613
NALCN	-1.451720305	-0.9677736	-2.616283175	0.009677924
ENPP2	-1.15648352	3.583011241	-2.613545927	0.00975275
RGS20	1.872745347	0.300639354	2.61293079	0.009769637
FPR2	1.351425649	-0.456253899	2.611194817	0.009817436

CMPK2	1.054950895	3.962454837	2.608849839	0.009882336
RIMBP2	-1.930262348	0.843804479	-2.608666492	0.009887427
LIMCH1	-1.115753036	4.07015995	-2.608604238	0.009889156
SLC7A7	1.073929892	3.834853334	2.60640465	0.009950421
SYNPR	-1.75764628	-2.338151684	-2.606236559	0.009955117
MMP7	2.251364192	3.931523238	2.60549006	0.009975995
GGT6	-1.832390055	3.516232802	-2.602585045	0.010057619
LANCL3	-1.12816844	0.739345049	-2.602015662	0.010073687
ZNF829	-1.259163594	0.487846783	-2.600026436	0.010130006
ALOXE3	1.864332346	0.90679847	2.59946073	0.010146074
PLEKHH1	-1.111237884	4.577256342	-2.598626701	0.010169804
ITGA7	-1.0534779	3.519935606	-2.598117062	0.010184329
GAST	1.897364667	-1.815192034	2.596542679	0.010229319
ANG	-1.321253791	2.124417847	-2.596273579	0.010237026
USP2	-1.106024021	1.688859963	-2.594650352	0.01028363
SLC18A1	-1.495873529	-2.566484957	-2.59403263	0.010301415
MAN1C1	-1.023184173	2.818830894	-2.593596818	0.010313979
FYB1	1.174756739	4.302484795	2.593090086	0.010328605
L3MBTL4	-1.672731013	0.890387685	-2.592745293	0.010338568
PAPPA2	-1.838484626	-0.791311275	-2.592366502	0.010349523
H2AW	1.106339409	2.898359831	2.592271404	0.010352274
APOBEC3A	2.107330655	1.444511775	2.59212866	0.010356406
ZNF345	-1.208106184	0.634727063	-2.591140314	0.010385056
CYB5R2	1.041952821	3.634080293	2.590923082	0.010391363
TERT	1.421782021	-0.408574006	2.587158455	0.010501203
ZNF43	-1.229696912	2.097277619	-2.58092373	0.010685405
UNC93A	1.814993275	-0.26159475	2.577298172	0.010793849
GATA3	1.23948515	1.706146965	2.575041745	0.010861837
ADAMTSL4	-1.142590235	4.214085402	-2.574450577	0.010879713
CPA3	-1.36491061	2.355327055	-2.574396626	0.010881346
C1QL3	-1.29802142	-0.439245647	-2.574028378	0.010892496
EPB41L4B	-1.034454362	4.925746453	-2.573344745	0.010913223
MUCL1	1.998987988	-1.645336201	2.568166667	0.011071365
ZNF793	-1.426546901	1.231707964	-2.567445006	0.011093567
MTMR8	-1.299415798	-0.258103477	-2.566782463	0.011113986
SCRG1	-1.507642291	-0.921747145	-2.565793318	0.011144533
VCAM1	1.091654147	3.881962195	2.564705161	0.011178224
SPARCL1	-1.123324607	6.644131866	-2.564624105	0.011180737
NMU	1.32309069	2.367921557	2.564272283	0.011191652
HTRA3	1.208031056	5.132731654	2.56334656	0.011220417
PHGR1	-3.37256596	0.372431605	-2.560806027	0.011299699
GPR50	2.052578719	-2.427201142	2.559560717	0.011338744
BMP5	-1.659939358	-1.269082209	-2.559354122	0.011345233
PPBP	1.696627661	-1.870680584	2.558872431	0.011360376
LYPD3	2.511571992	5.628708173	2.55801216	0.011387466
SMIM5	-1.337146098	1.599718081	-2.5573982	0.011406834
KCNK15	-1.681322214	1.014999931	-2.556847266	0.01142424
FAM221A	-1.350515426	2.975767298	-2.556637144	0.011430884
LZTS1	1.063596457	3.268814613	2.555906585	0.011454013
PLAC1	1.502321487	-1.131356477	2.555676119	0.011461318
LAMP5	1.533112101	0.237614484	2.554513741	0.011498226
MYO5C	-1.372532868	5.077943412	-2.553592132	0.011527564
CABYR	1.364151384	1.769708143	2.551493223	0.01159463

PLEKHA6	-1.930431513	4.983934963	-2.550324851	0.011632114
IL2RB	1.000413173	3.76895325	2.54943856	0.01166062
ADAMTS1	-1.120846814	5.106749194	-2.549377473	0.011662587
HOXD13	2.715237891	-0.736467111	2.54857655	0.011688406
MS4A8	-2.459412282	-0.970527735	-2.548377762	0.011694822
TACR2	-1.340841413	0.820301274	-2.548263898	0.011698498
RGS5	-1.18703936	6.715062463	-2.54807092	0.011704732
LIPH	-1.6981792	4.544668197	-2.547973698	0.011707873
PADI3	2.450253559	1.072388327	2.547430749	0.011725431
APOBEC3G	1.028236157	3.121076433	2.546271771	0.011762988
CDK5R1	1.171728727	2.980499551	2.542632649	0.011881616
SMIM6	-2.060127725	-0.190199169	-2.542363063	0.011890446
NTM	1.208402967	1.987776723	2.540926385	0.011937604
CHD5	-1.489611864	-0.392177973	-2.537979531	0.012034856
TFCP2L1	-1.185150575	5.522030404	-2.537624513	0.01204662
UBD	1.911888155	1.6997032	2.533632293	0.012179617
FFAR2	1.298011613	-0.045519043	2.533094291	0.01219764
ACP7	2.001120812	-0.756329395	2.53284907	0.012205863
FMO1	1.28274717	0.971027493	2.532497696	0.012217654
SOX21	-2.375169082	1.752857588	-2.529322937	0.012324651
IGSF9	1.094374103	5.403121091	2.528767498	0.012343457
FGFBP1	2.686402925	4.427523051	2.527785578	0.012376764
MYOM3	1.474931346	1.76318067	2.527577476	0.012383834
C1QTNF12	1.291504567	1.197993338	2.526835536	0.012409067
KCND3	-1.374943251	3.352213753	-2.525917855	0.012440341
HPSE2	-1.833643114	-0.414290737	-2.524320134	0.012494958
PODN	-1.392190306	3.663532171	-2.521977007	0.012575443
LTF	-2.496892333	1.946191116	-2.521953381	0.012576257
GP2	-2.97668981	-0.430001695	-2.517468497	0.012731613
NKX2-8	1.943196017	-1.159140481	2.517318439	0.01273684
PLXNA4	-1.3836143	1.556171275	-2.514631135	0.012830781
TPSB2	-1.375259479	2.316879721	-2.513516	0.012869944
AGMAT	1.401446292	2.604760393	2.51348444	0.012871054
WNT10A	1.618684969	2.609337392	2.513369465	0.012875098
TMEM52B	1.373044493	-0.820670388	2.513305576	0.012877346
CDO1	-1.601059339	-1.12214296	-2.512818089	0.012894509
ZNF556	1.347802496	0.132714913	2.511974035	0.012924273
CAMK2B	-1.502635347	-0.571191064	-2.511191719	0.012951916
PTPRS	-1.370549234	5.89215517	-2.510915055	0.012961704
SHISA9	-1.790857972	-1.989918355	-2.509939768	0.012996261
PBX1	-1.014576599	5.586647929	-2.509254129	0.013020605
CYP2C18	-1.825115339	2.425098075	-2.508285079	0.013055081
GATM	-1.49420861	5.053725305	-2.508143751	0.013060115
C1QB	1.228277039	5.802988408	2.507894737	0.013068991
GZMB	1.155998649	1.95031652	2.506434406	0.013121148
TESC	-2.280257549	2.337149222	-2.505721119	0.013146691
GJB6	3.173134477	3.556459966	2.504952127	0.013174278
MACROD2	-1.519434466	1.049085956	-2.503818905	0.013215027
EFCC1	-1.056352506	1.14464112	-2.501929237	0.013283226
TCHH	1.87610427	0.414174754	2.501360364	0.013303818
ZNF257	-1.252347856	-1.132324286	-2.499756015	0.013362046
ACKR2	-1.102722333	0.696701052	-2.496907182	0.013466002
RGS2	-1.052578479	4.814730487	-2.496847634	0.013468182

SLC7A9	1.450481926	-0.940663743	2.494359457	0.013559579
NTN1	-1.302188568	4.571935465	-2.494150635	0.013567275
LPIN2	-1.096875283	5.714115148	-2.491261139	0.013674161
ZNF577	-1.003973435	1.877379872	-2.490379562	0.013706921
KRT20	-3.353943239	0.704071665	-2.488607605	0.013772979
ANO1	1.418094344	7.115995825	2.488324218	0.01378357
EEF1A2	-2.024461826	0.981942519	-2.487727416	0.013805897
ARHGAP23	1.032827354	6.864124465	2.486589994	0.01384854
TMEM35A	-1.524367769	-1.012319313	-2.486310745	0.013859027
LRRC17	-1.498626418	1.286714547	-2.486065032	0.013868261
DOK7	-1.3522275	0.71534742	-2.485256144	0.013898696
EGF	1.755122292	-0.485967579	2.484326067	0.013933765
EN1	2.810958389	-0.4040465	2.483764747	0.013954968
FEZF1	1.892347717	-0.625974815	2.481182234	0.014052889
SLC7A2	-1.593064196	3.435109267	-2.480533089	0.014077598
LYPD5	1.225985525	2.957400589	2.479814398	0.014105
IDO1	1.88491979	2.838427541	2.477301064	0.014201201
TBX4	1.500393541	-0.979962787	2.475151921	0.014283925
FGF11	1.348674849	-0.244012394	2.475054658	0.014287679
BTBD16	1.257248194	-0.617844267	2.474494745	0.014309306
H2BC4	1.073464124	1.674139341	2.472551688	0.014384585
THBS2	1.328581821	6.310121578	2.471247636	0.014435304
BIRC7	1.39849032	-2.270399389	2.470276442	0.014473181
DUOX1	-1.349346029	5.96894375	-2.470047783	0.014482112
OMD	-1.61387356	0.234331446	-2.46940308	0.014507318
KIF19	-1.261525556	-0.847823228	-2.468184491	0.014555068
HOXC12	2.389993061	-0.988463627	2.468145734	0.014556589
COL8A1	1.365145877	4.724510604	2.465026597	0.01467946
FOXC2	1.562520459	1.009267083	2.463104774	0.014755625
SP5	-1.990398521	0.234267815	-2.460634247	0.014854051
SMIM24	-2.781739218	1.593232241	-2.460577375	0.014856323
STK31	1.330070538	1.935962422	2.459433122	0.014902114
ALOX15	1.902662659	-0.631667024	2.459263372	0.014908918
SAMD11	-1.118226945	0.781780928	-2.458076	0.014956586
CTF1	-1.096096357	2.317086648	-2.457725783	0.014970672
SVEP1	-1.01106046	3.597297791	-2.457648431	0.014973785
NIBAN1	-1.06689386	5.786481388	-2.453666247	0.015134808
ELOVL5	1.115911149	5.369307391	2.453301442	0.015149636
PGR	-1.254151119	0.230513047	-2.453287186	0.015150216
ZNF229	-1.524985604	0.090709238	-2.44756626	0.01538445
FGF19	2.524201724	-0.775282802	2.445218751	0.015481492
VWA7	-1.030409059	2.328975322	-2.443962012	0.015533666
LGALS9B	-1.549020115	0.643284993	-2.443154375	0.015567278
CRABP2	1.762258699	4.671984763	2.442473737	0.015595655
MAGEA6	3.039652218	-0.497888565	2.438383258	0.015767161
ZNF71	-1.038245524	1.62618828	-2.438326288	0.015769562
ZBED2	1.877808391	1.145687602	2.437819026	0.015790949
NPAS3	-1.240928831	-0.371487987	-2.435600412	0.015884795
VGLL1	1.711897132	-0.556255388	2.43494912	0.015912438
CYP3A5	-2.005300131	4.171749301	-2.434724945	0.015921963
CAND2	-1.183215343	1.278945765	-2.433583952	0.015970519
SPINK1	-3.215516159	2.695734906	-2.433458521	0.015975865
XPNPEP2	-1.992964167	-0.121208442	-2.432790442	0.016004365

CHRD	-1.000778958	1.768255793	-2.431903504	0.016042271
ZDHHC11B	-1.327836519	2.503854551	-2.431550047	0.0160574
RAMP1	-1.35188326	2.591232164	-2.429930779	0.016126868
AKAP12	-1.19295073	4.61310417	-2.429592856	0.016141399
NETO2	1.146661904	4.336007032	2.429314288	0.016153386
CACNA1H	-1.177219564	4.155104829	-2.429163066	0.016159897
HBA2	-1.327678876	1.889223201	-2.428546668	0.016186459
SLC38A3	-1.631431769	-0.973966601	-2.428004101	0.016209872
NOX1	1.442555767	0.534774568	2.42622837	0.016286708
SLCO1B3	2.227052106	-0.28588103	2.42560059	0.016313949
KCND2	1.30850872	0.009867242	2.425600425	0.016313956
XCL1	1.317339618	-0.191259808	2.422048912	0.016468828

adj.P.Val	B
2.67E-21	47.15268277
4.42E-19	41.23767422
4.42E-19	41.08796465
1.39E-18	39.68301256
3.48E-18	38.56994544
8.76E-18	37.48997857
1.36E-17	36.78534512
1.36E-17	36.78170499
1.59E-17	36.51384959
1.84E-17	36.26628381
1.86E-17	36.16218154
1.90E-17	35.99881347
1.90E-17	35.9784225
2.09E-17	35.81408869
2.20E-17	35.69410235
2.95E-17	35.31051073
2.95E-17	35.2866884
3.59E-17	35.03920133
3.61E-17	34.97954136
4.81E-17	34.64920179
9.98E-17	33.88778854
1.13E-16	33.7245293
1.16E-16	33.65202209
1.66E-16	33.25931499
1.73E-16	33.162992
1.73E-16	33.12891683
1.73E-16	33.0747525
1.73E-16	33.0693346
1.79E-16	32.99711226
1.79E-16	32.96704205
2.04E-16	32.80717321
2.05E-16	32.77093952
2.05E-16	32.70933953
2.05E-16	32.70086807
2.05E-16	32.68285037
2.35E-16	32.52346381
2.43E-16	32.46461458
3.35E-16	32.12489784
5.11E-16	31.68644409
8.26E-16	31.19331002
9.96E-16	30.98589247
1.12E-15	30.84783062
1.19E-15	30.76731896
3.13E-15	29.77682546
3.13E-15	29.77650211
3.16E-15	29.73853961
3.16E-15	29.70701036
3.16E-15	29.70453329
3.22E-15	29.65835804
3.22E-15	29.64478311

3.22E-15	29.62742169
4.11E-15	29.36968834
5.65E-15	29.03914007
6.13E-15	28.93465427
6.13E-15	28.92307525
6.22E-15	28.87445813
6.22E-15	28.87370484
6.24E-15	28.85466015
8.19E-15	28.57159737
8.21E-15	28.55326055
8.95E-15	28.45229997
9.12E-15	28.41878166
9.35E-15	28.37840317
9.44E-15	28.34580462
9.44E-15	28.33836326
1.43E-14	27.91617363
1.47E-14	27.87897073
1.78E-14	27.67394782
1.82E-14	27.63880277
2.23E-14	27.42662063
2.27E-14	27.39382401
3.05E-14	27.09223951
3.05E-14	27.07839788
3.15E-14	27.03383904
3.27E-14	26.98396809
3.27E-14	26.97287739
3.37E-14	26.92973111
3.40E-14	26.90934126
3.58E-14	26.84583505
3.75E-14	26.78865837
3.77E-14	26.77145957
4.58E-14	26.56820443
4.76E-14	26.51734496
4.76E-14	26.49271795
4.76E-14	26.48542514
4.76E-14	26.47447159
4.76E-14	26.47365953
4.87E-14	26.44085445
5.50E-14	26.31097512
5.50E-14	26.29999399
7.03E-14	26.04846227
7.16E-14	26.02082589
8.96E-14	25.79092006
9.17E-14	25.75791689
9.17E-14	25.74796689
9.41E-14	25.70898481
9.41E-14	25.70239632
9.73E-14	25.65941921
1.03E-13	25.59536568
1.07E-13	25.54264206
1.10E-13	25.51120752
1.26E-13	25.36885504
1.54E-13	25.16368311

1.67E-13	25.06413838
1.67E-13	25.05772373
1.67E-13	25.05300338
1.72E-13	25.01735713
1.82E-13	24.95424053
1.83E-13	24.9361263
2.37E-13	24.67872907
2.43E-13	24.64352311
2.68E-13	24.54010717
2.80E-13	24.48869162
2.91E-13	24.44145831
2.99E-13	24.40846737
2.99E-13	24.39907803
3.10E-13	24.35430948
3.56E-13	24.21096933
3.77E-13	24.14887666
5.26E-13	23.81516014
5.69E-13	23.72895241
6.31E-13	23.62070681
6.69E-13	23.55549941
7.10E-13	23.48997444
7.41E-13	23.44077909
9.50E-13	23.19039954
1.17E-12	22.97516433
1.24E-12	22.91616934
1.61E-12	22.65087505
1.78E-12	22.54680857
2.72E-12	22.12403865
2.72E-12	22.11736645
2.89E-12	22.05098124
2.94E-12	22.02655868
3.14E-12	21.95644216
3.23E-12	21.92195138
3.28E-12	21.89983222
3.30E-12	21.88792745
3.76E-12	21.75185086
4.00E-12	21.68452942
4.36E-12	21.59414283
4.61E-12	21.53232322
4.70E-12	21.506821
5.68E-12	21.31536242
6.31E-12	21.20566203
7.19E-12	21.0726663
7.44E-12	21.03211365
8.17E-12	20.93421026
8.43E-12	20.89705039
8.59E-12	20.8726324
1.29E-11	20.47002303
1.36E-11	20.41365192
1.53E-11	20.29089772
2.43E-11	19.83173517
2.54E-11	19.78487163
2.62E-11	19.74822882

2.63E-11	19.73596752
2.75E-11	19.68866651
3.07E-11	19.57561304
3.10E-11	19.560033
3.21E-11	19.51766779
3.49E-11	19.43152311
3.58E-11	19.3992883
3.65E-11	19.37447915
3.74E-11	19.34563581
4.18E-11	19.23226755
5.40E-11	18.97612909
5.89E-11	18.8816043
5.89E-11	18.87807488
5.89E-11	18.8745244
5.89E-11	18.86838696
6.15E-11	18.82084772
7.07E-11	18.67976296
7.25E-11	18.64926886
7.45E-11	18.61797435
7.62E-11	18.59021578
8.31E-11	18.49949175
1.07E-10	18.25069205
1.20E-10	18.12809782
1.31E-10	18.04010912
1.39E-10	17.97695977
1.42E-10	17.953084
1.43E-10	17.93851149
1.44E-10	17.92521881
1.48E-10	17.89364352
1.49E-10	17.88374431
1.54E-10	17.84397685
1.75E-10	17.71369128
1.91E-10	17.62511257
2.12E-10	17.51826085
2.21E-10	17.47379989
2.55E-10	17.33058571
2.57E-10	17.31538053
2.62E-10	17.29458168
2.78E-10	17.22981886
2.94E-10	17.17212591
3.89E-10	16.89281446
4.07E-10	16.84491548
4.41E-10	16.76091278
4.43E-10	16.75142277
4.56E-10	16.71957411
5.42E-10	16.54687932
5.55E-10	16.5176782
5.84E-10	16.46450387
5.96E-10	16.43912814
6.05E-10	16.41966488
6.14E-10	16.40100125
6.76E-10	16.30320211
7.64E-10	16.17922066

7.80E-10	16.15403809
7.89E-10	16.1382555
7.99E-10	16.12130302
8.77E-10	16.02642897
9.99E-10	15.8959152
9.99E-10	15.89138398
1.08E-09	15.80691664
1.09E-09	15.79450556
1.13E-09	15.75347358
1.18E-09	15.71168006
1.18E-09	15.7043979
1.18E-09	15.70243259
1.19E-09	15.68571589
1.31E-09	15.5926388
1.31E-09	15.58548254
1.34E-09	15.55905248
1.40E-09	15.51566962
1.50E-09	15.44000045
1.50E-09	15.43974677
1.64E-09	15.34125474
1.72E-09	15.29370232
1.75E-09	15.2707558
1.75E-09	15.26778046
1.97E-09	15.14954844
2.07E-09	15.09611877
2.11E-09	15.07416921
2.11E-09	15.06853806
2.22E-09	15.01707824
2.54E-09	14.88278092
2.83E-09	14.77091212
2.87E-09	14.75392315
2.93E-09	14.73051135
3.11E-09	14.66704649
3.36E-09	14.58780297
3.43E-09	14.56489262
3.49E-09	14.544634
3.58E-09	14.51579197
3.59E-09	14.50930043
3.66E-09	14.48776189
3.72E-09	14.46660025
4.01E-09	14.39116611
4.37E-09	14.30260313
4.69E-09	14.23085122
5.39E-09	14.09099053
5.61E-09	14.04856681
5.67E-09	14.03376694
6.03E-09	13.97076458
6.41E-09	13.90781925
7.05E-09	13.8084293
7.23E-09	13.77924039
7.51E-09	13.73949521
7.59E-09	13.72459274
8.11E-09	13.65673036

1.00E-08	13.44949593
1.07E-08	13.37931583
1.15E-08	13.30784755
1.16E-08	13.29617995
1.16E-08	13.2936845
1.19E-08	13.2659183
1.21E-08	13.24682576
1.25E-08	13.21152818
1.26E-08	13.19447116
1.27E-08	13.18798503
1.31E-08	13.15141432
1.33E-08	13.12980657
1.33E-08	13.1295983
1.35E-08	13.11567806
1.42E-08	13.06036852
1.51E-08	12.99815682
1.51E-08	12.99180918
1.53E-08	12.97789459
1.55E-08	12.962931
1.58E-08	12.9374569
1.63E-08	12.90753456
1.65E-08	12.89307862
1.68E-08	12.87209166
1.68E-08	12.86635676
1.69E-08	12.85981449
1.74E-08	12.82786035
1.74E-08	12.82342252
1.75E-08	12.81578962
1.79E-08	12.78724987
1.82E-08	12.77038975
1.92E-08	12.71090763
1.92E-08	12.70775311
1.96E-08	12.68334165
2.07E-08	12.62695753
2.09E-08	12.61264689
2.09E-08	12.61084836
2.10E-08	12.6047498
2.15E-08	12.57846322
2.16E-08	12.57048562
2.17E-08	12.56057023
2.32E-08	12.49363919
2.40E-08	12.45837079
2.44E-08	12.43960181
2.47E-08	12.42313642
2.62E-08	12.36345706
2.64E-08	12.35361882
3.10E-08	12.19181031
3.15E-08	12.17305252
3.15E-08	12.16986464
3.18E-08	12.15788992
3.23E-08	12.13921662
3.39E-08	12.09038238
3.43E-08	12.07617581

3.52E-08	12.04605204
3.61E-08	12.02041898
3.97E-08	11.92422925
4.02E-08	11.9096898
4.05E-08	11.89837561
4.88E-08	11.71228664
5.17E-08	11.65321365
5.20E-08	11.64289131
5.20E-08	11.64154871
5.41E-08	11.60028426
5.42E-08	11.59626287
5.62E-08	11.55847246
5.70E-08	11.53868158
5.75E-08	11.52670183
5.87E-08	11.5039456
6.32E-08	11.42935569
6.38E-08	11.41789119
6.60E-08	11.3814315
6.66E-08	11.36994745
6.93E-08	11.32892773
7.05E-08	11.31026385
7.27E-08	11.27732524
7.29E-08	11.27202435
7.70E-08	11.21252501
7.81E-08	11.19621537
8.10E-08	11.15869618
8.17E-08	11.1470253
8.39E-08	11.11832094
8.39E-08	11.11600579
8.41E-08	11.11058456
8.82E-08	11.05884856
9.36E-08	10.99933666
9.39E-08	10.99364391
9.85E-08	10.94400254
1.08E-07	10.85187518
1.14E-07	10.80136518
1.14E-07	10.79344762
1.14E-07	10.79334761
1.15E-07	10.78340627
1.15E-07	10.7815486
1.15E-07	10.77191235
1.16E-07	10.76768371
1.18E-07	10.74445429
1.18E-07	10.74231255
1.18E-07	10.73616121
1.20E-07	10.71917142
1.22E-07	10.70084994
1.23E-07	10.69265964
1.27E-07	10.65232954
1.27E-07	10.6501993
1.31E-07	10.62054659
1.36E-07	10.58511022
1.37E-07	10.57578923

1.38E-07	10.56293427
1.47E-07	10.50107822
1.50E-07	10.47602361
1.51E-07	10.46823212
1.54E-07	10.44769203
1.61E-07	10.39564714
1.65E-07	10.37193218
1.65E-07	10.3672817
1.67E-07	10.34854037
1.69E-07	10.33420506
1.70E-07	10.32941109
1.74E-07	10.30497569
1.76E-07	10.28861515
1.97E-07	10.18045142
1.98E-07	10.16860791
1.98E-07	10.16828099
2.01E-07	10.15097732
2.02E-07	10.14425604
2.04E-07	10.13365504
2.12E-07	10.09250795
2.12E-07	10.08565378
2.12E-07	10.08533869
2.14E-07	10.07479924
2.30E-07	10.003568
2.40E-07	9.952655084
2.40E-07	9.952075866
2.44E-07	9.933192979
2.52E-07	9.900652627
2.53E-07	9.894589362
2.54E-07	9.886490855
2.55E-07	9.881483529
2.57E-07	9.868045752
2.57E-07	9.867031414
2.63E-07	9.842593477
2.86E-07	9.76156463
2.89E-07	9.747928219
2.95E-07	9.726086091
3.16E-07	9.658374094
3.20E-07	9.642472173
3.35E-07	9.596249884
3.35E-07	9.594271429
3.39E-07	9.579712083
3.47E-07	9.554349276
3.64E-07	9.50628773
3.64E-07	9.505868969
3.80E-07	9.460421416
3.86E-07	9.444353398
3.86E-07	9.442307814
3.87E-07	9.437581793
3.87E-07	9.434583046
3.91E-07	9.419342777
3.91E-07	9.419018448
3.94E-07	9.41113778

4.36E-07	9.307590089
4.37E-07	9.304528318
4.42E-07	9.290578297
4.54E-07	9.263297198
4.55E-07	9.257925547
4.61E-07	9.244339568
4.75E-07	9.209733075
4.93E-07	9.171309764
5.21E-07	9.115605852
5.21E-07	9.113783236
5.25E-07	9.105325842
5.27E-07	9.099263494
5.45E-07	9.06451874
5.52E-07	9.049714596
5.69E-07	9.018858356
5.73E-07	9.01053923
6.22E-07	8.929288522
6.34E-07	8.906476699
6.64E-07	8.859032533
6.79E-07	8.835447355
6.88E-07	8.818138836
7.23E-07	8.768602862
7.62E-07	8.716440295
7.68E-07	8.706065482
7.93E-07	8.673268233
8.13E-07	8.645582827
8.21E-07	8.632436397
8.21E-07	8.631231971
8.36E-07	8.609995116
8.36E-07	8.60958731
8.58E-07	8.581024273
8.60E-07	8.576091819
8.67E-07	8.566860864
8.79E-07	8.550791506
8.99E-07	8.526257194
8.99E-07	8.525594818
9.33E-07	8.485510019
9.68E-07	8.447786826
9.88E-07	8.424741905
1.04E-06	8.373478099
1.19E-06	8.236992755
1.21E-06	8.220154443
1.21E-06	8.215592113
1.22E-06	8.212600084
1.27E-06	8.169666121
1.28E-06	8.156230633
1.30E-06	8.141462273
1.31E-06	8.129466084
1.34E-06	8.106162322
1.36E-06	8.087826503
1.37E-06	8.078088194
1.41E-06	8.050886183
1.41E-06	8.05074632

1.47E-06	8.009242352
1.50E-06	7.98689303
1.58E-06	7.935096572
1.62E-06	7.909143503
1.67E-06	7.877194276
1.67E-06	7.87385526
1.76E-06	7.822966813
1.77E-06	7.809922751
1.80E-06	7.794229333
1.80E-06	7.792880613
1.81E-06	7.785211858
1.84E-06	7.767295357
1.84E-06	7.764396249
1.89E-06	7.73503406
1.89E-06	7.731794658
1.89E-06	7.731237597
1.93E-06	7.713690345
1.93E-06	7.711182688
1.95E-06	7.695250356
2.00E-06	7.66388789
2.00E-06	7.662122529
2.00E-06	7.661160635
2.06E-06	7.634300887
2.09E-06	7.618640867
2.21E-06	7.560316185
2.24E-06	7.544519231
2.29E-06	7.520224357
2.30E-06	7.516356716
2.32E-06	7.506299892
2.36E-06	7.485674943
2.39E-06	7.472824028
2.50E-06	7.426809593
2.52E-06	7.414821124
2.54E-06	7.406162367
2.64E-06	7.367106507
2.65E-06	7.362544288
2.66E-06	7.354460931
2.67E-06	7.350611163
2.70E-06	7.336543096
2.71E-06	7.333670519
2.73E-06	7.324394159
2.85E-06	7.281164953
2.94E-06	7.247324917
2.99E-06	7.23008546
3.08E-06	7.201055559
3.09E-06	7.195779623
3.11E-06	7.18817457
3.11E-06	7.186967028
3.18E-06	7.160066196
3.27E-06	7.132261329
3.27E-06	7.130213735
3.30E-06	7.116909211
3.30E-06	7.115480412

3.32E-06	7.107549962
3.36E-06	7.094819376
3.37E-06	7.089452672
3.52E-06	7.046047871
3.53E-06	7.040641809
3.67E-06	7.003297543
3.74E-06	6.983644902
3.78E-06	6.969875215
3.80E-06	6.963321203
3.97E-06	6.91938212
3.98E-06	6.914464043
3.98E-06	6.910182
3.99E-06	6.906744098
4.01E-06	6.900394613
4.25E-06	6.840019815
4.36E-06	6.814179602
4.39E-06	6.804183255
4.47E-06	6.783683197
4.50E-06	6.775157424
4.58E-06	6.754031501
4.67E-06	6.734415736
4.70E-06	6.727441711
4.77E-06	6.710412724
4.82E-06	6.699914582
4.92E-06	6.678753285
5.12E-06	6.635874539
5.18E-06	6.623619278
5.19E-06	6.619421403
5.36E-06	6.585650691
5.56E-06	6.547924207
5.56E-06	6.546367594
5.60E-06	6.537325684
5.75E-06	6.511042603
5.77E-06	6.50514608
5.79E-06	6.50122176
6.07E-06	6.452644921
6.09E-06	6.447497985
6.13E-06	6.440002024
6.16E-06	6.433691515
6.33E-06	6.402350645
6.47E-06	6.378535496
6.49E-06	6.374467786
6.50E-06	6.370671417
6.54E-06	6.360945537
6.54E-06	6.359527327
6.68E-06	6.333145818
6.72E-06	6.32621735
7.00E-06	6.285425064
7.00E-06	6.284939629
7.07E-06	6.271335423
7.07E-06	6.271109296
7.59E-06	6.196555626
7.59E-06	6.194721671

7.63E-06	6.18763966
7.69E-06	6.179434864
7.72E-06	6.17313591
7.79E-06	6.163226521
7.79E-06	6.161014587
7.79E-06	6.160929603
7.80E-06	6.157371071
8.11E-06	6.11766489
8.56E-06	6.062419682
8.73E-06	6.04241016
8.79E-06	6.034678432
8.80E-06	6.031543822
8.80E-06	6.029761285
9.05E-06	6.002121863
9.06E-06	5.999570324
9.07E-06	5.99698088
9.16E-06	5.9857697
9.19E-06	5.981335361
9.29E-06	5.968941284
9.33E-06	5.964305604
9.38E-06	5.956205032
9.61E-06	5.929962158
9.80E-06	5.906298759
9.86E-06	5.899438106
1.01E-05	5.86903721
1.02E-05	5.861171976
1.02E-05	5.85958249
1.04E-05	5.839520202
1.04E-05	5.837878876
1.05E-05	5.823418044
1.06E-05	5.805500449
1.06E-05	5.80380614
1.08E-05	5.789230753
1.10E-05	5.767869908
1.18E-05	5.692389806
1.21E-05	5.672985739
1.21E-05	5.668461958
1.21E-05	5.668420269
1.27E-05	5.619721541
1.31E-05	5.591562456
1.33E-05	5.57604402
1.35E-05	5.556944479
1.36E-05	5.550071518
1.36E-05	5.544249903
1.37E-05	5.539064404
1.39E-05	5.52664296
1.40E-05	5.511928275
1.46E-05	5.467837455
1.51E-05	5.433242314
1.53E-05	5.419493937
1.55E-05	5.405248049
1.56E-05	5.395977596
1.62E-05	5.360562162

1.62E-05	5.356498862
1.62E-05	5.355726975
1.62E-05	5.350490842
1.62E-05	5.350478185
1.63E-05	5.344895463
1.68E-05	5.312903723
1.73E-05	5.279257814
1.78E-05	5.253835353
1.81E-05	5.235952435
1.81E-05	5.232086453
1.85E-05	5.21109427
1.86E-05	5.203836364
1.89E-05	5.189929971
1.90E-05	5.181565586
1.90E-05	5.179426114
1.91E-05	5.171463388
1.93E-05	5.158835823
1.94E-05	5.15509666
1.94E-05	5.15222952
1.95E-05	5.148592924
1.98E-05	5.129178756
1.98E-05	5.128228869
1.99E-05	5.122160705
2.11E-05	5.062029001
2.12E-05	5.056551031
2.14E-05	5.046188032
2.16E-05	5.036500166
2.20E-05	5.016626035
2.21E-05	5.008239066
2.27E-05	4.98322143
2.29E-05	4.973311194
2.31E-05	4.964020108
2.42E-05	4.915714162
2.58E-05	4.846877795
2.61E-05	4.836300034
2.63E-05	4.825328422
2.64E-05	4.820860611
2.65E-05	4.815044667
2.67E-05	4.80751953
2.69E-05	4.798698341
2.72E-05	4.785496668
2.72E-05	4.783231962
2.84E-05	4.740832807
2.85E-05	4.73286758
2.89E-05	4.718444834
2.90E-05	4.713203223
2.92E-05	4.702190604
2.99E-05	4.674196603
2.99E-05	4.672096789
3.00E-05	4.66819257
3.04E-05	4.654325695
3.06E-05	4.645062361
3.18E-05	4.605088756

3.20E-05	4.598037729
3.20E-05	4.597851544
3.21E-05	4.59216215
3.23E-05	4.585955564
3.26E-05	4.575643954
3.26E-05	4.574567971
3.30E-05	4.562811434
3.32E-05	4.554295753
3.39E-05	4.532677148
3.42E-05	4.523366602
3.42E-05	4.523113099
3.47E-05	4.507080184
3.48E-05	4.503354661
3.67E-05	4.447600012
3.67E-05	4.446393259
3.67E-05	4.445614704
3.67E-05	4.445171274
3.72E-05	4.429333917
3.75E-05	4.420795795
3.79E-05	4.407775413
3.82E-05	4.400105698
3.86E-05	4.388080874
3.88E-05	4.379742113
3.94E-05	4.362828714
4.02E-05	4.341939804
4.07E-05	4.327185184
4.08E-05	4.323980667
4.09E-05	4.319140205
4.14E-05	4.307736224
4.15E-05	4.303088242
4.20E-05	4.291021292
4.30E-05	4.264834366
4.32E-05	4.259238396
4.38E-05	4.243831106
4.42E-05	4.234336711
4.43E-05	4.228899832
4.45E-05	4.220135968
4.60E-05	4.187805023
4.63E-05	4.179622498
4.77E-05	4.147646473
4.78E-05	4.144102228
4.84E-05	4.132243303
4.94E-05	4.110918722
4.99E-05	4.097346979
5.06E-05	4.082551241
5.07E-05	4.080548097
5.07E-05	4.080183371
5.12E-05	4.069335691
5.14E-05	4.064734764
5.15E-05	4.060984285
5.28E-05	4.035042795
5.35E-05	4.021736583
5.44E-05	4.004693873

5.48E-05	3.996553952
5.52E-05	3.987616628
5.55E-05	3.980147001
5.60E-05	3.972134265
5.68E-05	3.955864871
5.74E-05	3.945934102
5.77E-05	3.93899676
5.92E-05	3.913267578
5.93E-05	3.909861764
6.03E-05	3.892072089
6.04E-05	3.889585036
6.05E-05	3.886919348
6.13E-05	3.873832559
6.14E-05	3.870776963
6.17E-05	3.864913546
6.25E-05	3.849746831
6.25E-05	3.847684602
6.30E-05	3.839825427
6.31E-05	3.837292111
6.62E-05	3.786488177
6.78E-05	3.762608294
6.89E-05	3.745194768
7.14E-05	3.708340417
7.23E-05	3.693575531
7.29E-05	3.684313612
7.33E-05	3.677116321
7.34E-05	3.674827818
7.38E-05	3.667705599
7.55E-05	3.644279837
7.55E-05	3.644039679
7.56E-05	3.640653653
7.60E-05	3.63486245
7.64E-05	3.628374397
7.99E-05	3.582166931
8.02E-05	3.576471711
8.03E-05	3.573628258
8.20E-05	3.552754555
8.20E-05	3.552582209
8.43E-05	3.522168898
8.46E-05	3.516275133
8.46E-05	3.516161411
8.57E-05	3.500970167
8.62E-05	3.492408558
8.79E-05	3.469565273
8.89E-05	3.457227296
9.01E-05	3.443270711
9.12E-05	3.430775413
9.22E-05	3.417792641
9.26E-05	3.411257928
9.27E-05	3.407746859
9.43E-05	3.389294921
9.45E-05	3.385853972
9.47E-05	3.383359455

9.71E-05	3.358094686
9.76E-05	3.35212217
9.98E-05	3.329299334
9.98E-05	3.327694734
1.00E-04	3.325236905
0.000100911	3.315387949
0.000101295	3.309948115
0.000103705	3.284647413
0.000103944	3.28148018
0.000105008	3.270822584
0.000109489	3.230081421
0.000110353	3.221636553
0.000110985	3.21522943
0.000111014	3.214014066
0.000111209	3.211381382
0.000111697	3.205289689
0.00011254	3.196210381
0.000112673	3.1932701
0.000112673	3.193164185
0.000113559	3.181633595
0.000113559	3.179448418
0.000113559	3.179238449
0.000113559	3.179022837
0.0001152	3.158734546
0.00011539	3.154334987
0.000115896	3.147918582
0.000115896	3.147346577
0.000116399	3.142282712
0.00011757	3.128619737
0.000118783	3.117381823
0.00011978	3.108497742
0.000120066	3.104372901
0.000121645	3.09101304
0.000121925	3.087402105
0.000121925	3.086966833
0.000122558	3.075805073
0.000122558	3.073729224
0.000124816	3.054532229
0.000125097	3.049645921
0.000125542	3.045353507
0.00012573	3.04209876
0.000126108	3.038333256
0.000126304	3.035945279
0.000129249	3.011305438
0.000129403	3.009004635
0.000131186	2.990823898
0.000134495	2.964033359
0.000134495	2.962842469
0.00013469	2.959473578
0.000134932	2.955208482
0.000134932	2.9550894
0.000135666	2.949036169
0.000135821	2.947060711

0.000137162	2.933275113
0.000138416	2.923739129
0.000141235	2.898388255
0.000141876	2.891448986
0.000141997	2.888881305
0.000144596	2.869896702
0.000144942	2.865897328
0.000145334	2.861566314
0.000146207	2.854133164
0.000146443	2.851729516
0.000147671	2.842928749
0.000148949	2.833006543
0.000150781	2.820527149
0.000151882	2.812749454
0.00015294	2.804425765
0.000156089	2.782478626
0.000157104	2.775460976
0.000157156	2.774288692
0.000158184	2.767052567
0.000158184	2.766377245
0.000159614	2.756116759
0.000159903	2.752686619
0.00016184	2.7386916
0.00016253	2.733796911
0.000163391	2.727926243
0.000164232	2.72025085
0.000164232	2.719657745
0.000165653	2.710625058
0.00016608	2.704021909
0.00016608	2.701509876
0.00016608	2.700587364
0.000167753	2.68939473
0.000167956	2.687406381
0.00017237	2.660265106
0.000173164	2.655069717
0.000173416	2.652853573
0.000174073	2.647597609
0.000175389	2.637123263
0.000175607	2.635113704
0.000175638	2.634115689
0.000176477	2.627113085
0.000178075	2.617731479
0.000179189	2.610158431
0.00018129	2.596627881
0.000182107	2.589901385
0.000183334	2.582468874
0.000183334	2.58188673
0.000184092	2.576339321
0.000184894	2.570578873
0.000186413	2.56118894
0.000188474	2.549124547
0.000189147	2.54331045
0.000191581	2.528538699

0.000192454	2.522823191
0.00019305	2.518989815
0.000193812	2.512929921
0.000195119	2.504406903
0.000200718	2.474292444
0.000200981	2.472063194
0.000201162	2.470272896
0.000201162	2.469611982
0.00020374	2.454879589
0.00020374	2.454350578
0.000204082	2.451173984
0.000204398	2.448911655
0.000208002	2.430097238
0.000208002	2.429963257
0.000209689	2.419142293
0.000210314	2.415536284
0.000214001	2.398267652
0.000222884	2.356564985
0.000226539	2.340359987
0.00022657	2.339451116
0.000228074	2.331612482
0.000229004	2.32697723
0.000230288	2.320897206
0.000232975	2.30460111
0.000232975	2.302911433
0.000237128	2.284617733
0.000239127	2.275039972
0.000239127	2.273672383
0.000239127	2.273578287
0.00023913	2.272795521
0.000241543	2.262510594
0.000246675	2.238755254
0.000246937	2.236982921
0.000249399	2.22681733
0.000250326	2.222538316
0.000251849	2.214504263
0.000256719	2.19483781
0.000258302	2.187495673
0.000258587	2.185690938
0.000258845	2.1832325
0.000258878	2.18235382
0.000261067	2.173620077
0.000262956	2.163771725
0.000263791	2.160017442
0.000267247	2.143930831
0.000267653	2.141743694
0.000270522	2.129398972
0.000270984	2.126290863
0.000271986	2.122049372
0.000272495	2.119533849
0.000275543	2.10528047
0.000276627	2.099337633
0.000280279	2.086178519

0.000282124	2.078488934
0.000283589	2.072847778
0.000284258	2.069141895
0.000286757	2.059918668
0.000287704	2.055526532
0.000287784	2.054330724
0.000287784	2.053791874
0.000290992	2.040367866
0.000291099	2.038601757
0.000291099	2.038021707
0.000291099	2.037827876
0.000292649	2.032071058
0.000301726	1.996548505
0.000301726	1.995893961
0.000303046	1.991038413
0.000304236	1.986606537
0.000305748	1.980471686
0.000305921	1.97905548
0.000306259	1.976730232
0.000306494	1.97528521
0.000308678	1.967128939
0.000310567	1.959921256
0.000312265	1.951897575
0.000313358	1.947876131
0.000314581	1.943476959
0.000315221	1.940550328
0.000315546	1.938228883
0.000324329	1.908206801
0.000325264	1.90463893
0.000325264	1.904065514
0.000326303	1.900342065
0.000331334	1.884455107
0.000331889	1.881462439
0.000338313	1.859467946
0.000338365	1.85756208
0.000341017	1.849477244
0.000347186	1.831146392
0.000349223	1.824194969
0.000351856	1.816394344
0.000352454	1.814092418
0.000354662	1.807488905
0.000355044	1.805777111
0.000357467	1.798649775
0.000362384	1.78503821
0.000367092	1.770754096
0.000368008	1.767011704
0.000373309	1.752800113
0.000377322	1.740436719
0.000377322	1.739933458
0.000380872	1.72901633
0.000383103	1.72212034
0.00038323	1.72112066
0.000385393	1.714429018

0.000385573	1.713303922
0.000389778	1.700999225
0.00039292	1.692730455
0.000401272	1.670808565
0.000403712	1.663479143
0.000403712	1.663039298
0.000410614	1.646343438
0.000414675	1.636368762
0.000416693	1.630426544
0.000418701	1.624529711
0.000421827	1.616150756
0.000423757	1.611164986
0.000429476	1.597152985
0.000432433	1.589374782
0.000432433	1.589325731
0.000440624	1.566904224
0.000441164	1.56457626
0.000442497	1.560891589
0.000443218	1.558685077
0.000447212	1.548880024
0.000449129	1.543507393
0.000450933	1.53772858
0.000450965	1.53699733
0.00045479	1.527695809
0.00045487	1.526203332
0.000457017	1.520308454
0.000457281	1.518567967
0.000460662	1.508788005
0.000460662	1.508318253
0.000466785	1.493226483
0.00047215	1.479819913
0.000474299	1.474879923
0.000476985	1.467933939
0.000476985	1.467469252
0.00048011	1.458172
0.000481657	1.453835496
0.000484411	1.447806762
0.000485858	1.444343087
0.00048698	1.441518844
0.000487816	1.439253546
0.00048821	1.437844071
0.00049061	1.43127818
0.000492737	1.425905313
0.000492961	1.424832795
0.000494136	1.420755242
0.000494136	1.420656777
0.00049894	1.409601078
0.000501225	1.404648605
0.000510175	1.382201455
0.000526856	1.34552398
0.000526856	1.345504589
0.000536738	1.324820823
0.000539068	1.319476111

0.000544602	1.30795804
0.000553817	1.290176889
0.000561114	1.276660464
0.000561953	1.274006879
0.000561953	1.274000109
0.000564115	1.269755123
0.000565663	1.266547536
0.000566303	1.264857244
0.000573566	1.25159712
0.000576386	1.246350038
0.000578049	1.242390797
0.0005781	1.241685951
0.00058021	1.237402177
0.00058021	1.23700945
0.000581263	1.233555735
0.000581263	1.233440352
0.00059731	1.205300675
0.000598555	1.202720636
0.000600114	1.198785657
0.000600114	1.198420665
0.000606274	1.188181793
0.000613198	1.176249869
0.000614516	1.172998768
0.000621018	1.16246843
0.000622029	1.160323427
0.000622188	1.15946904
0.000633932	1.140629203
0.000636598	1.13545287
0.000642408	1.126284497
0.000642776	1.125134246
0.000648699	1.115884951
0.000649228	1.114507858
0.000651098	1.11118941
0.000658514	1.097902902
0.000658514	1.097482879
0.000663374	1.088244566
0.000665814	1.084071784
0.000669943	1.077041085
0.000675815	1.068219234
0.000676278	1.066971757
0.000680109	1.058641153
0.000680429	1.05759755
0.000685349	1.049806809
0.000685349	1.04961195
0.000689778	1.04234837
0.000692923	1.034562733
0.00069293	1.033871182
0.000698132	1.025636907
0.00070136	1.019509423
0.000705847	1.012910825
0.000710201	1.006529451
0.000714293	0.999933963
0.000715261	0.998066299

0.000718086	0.993763561
0.000719633	0.991146095
0.000726551	0.981551022
0.000728811	0.976907761
0.000730063	0.974649301
0.0007335	0.969049401
0.000746087	0.945995303
0.000747504	0.943625983
0.0007476	0.942920779
0.000749847	0.93834609
0.000751512	0.935677202
0.000756667	0.927634396
0.000757901	0.925384964
0.000772406	0.904649922
0.000779497	0.894896518
0.000782909	0.890209544
0.000796043	0.87398413
0.000806372	0.860128914
0.000811649	0.853185292
0.000811649	0.852417206
0.000813017	0.850108057
0.00081709	0.844298148
0.00081709	0.844259469
0.000819839	0.840526667
0.000825098	0.833366857
0.000840262	0.814527282
0.000843331	0.810527012
0.000844382	0.807916808
0.000849223	0.7999879
0.000849485	0.79912886
0.000865842	0.778360974
0.000867596	0.775891955
0.000871757	0.770827929
0.000875468	0.76622407
0.000875468	0.765703318
0.000875793	0.764788687
0.000877007	0.76292151
0.000881987	0.756470422
0.000887371	0.749623741
0.000893126	0.742985676
0.000894537	0.740938476
0.000902814	0.73172151
0.000908938	0.723683407
0.000921067	0.709545552
0.000921664	0.708376975
0.000924598	0.704484862
0.000927372	0.700896979
0.000930893	0.696778854
0.00093752	0.689556305
0.000942159	0.681574522
0.000945967	0.67722928
0.000951043	0.67109201
0.000953056	0.66799576

0.000953191	0.666753337
0.000955738	0.663693636
0.00095668	0.662214233
0.000964394	0.653566493
0.000984565	0.632470954
0.000999081	0.617781135
0.000999081	0.617625818
0.001015777	0.601515375
0.001029961	0.586989891
0.001035879	0.580920217
0.001036245	0.580039007
0.001047339	0.568398905
0.001049143	0.566235207
0.001050746	0.564254869
0.001055481	0.558941613
0.001074237	0.539137957
0.001078564	0.534820671
0.001081382	0.531044733
0.001081382	0.530740893
0.001086192	0.524405859
0.001090786	0.519903752
0.001098859	0.510280989
0.001111372	0.498479832
0.001111372	0.498039682
0.001114596	0.494783255
0.001117969	0.49140945
0.001124795	0.485161438
0.001136161	0.474117144
0.001138387	0.471207896
0.001143895	0.465608204
0.001150924	0.457186308
0.001189009	0.422393359
0.00119218	0.418942997
0.00119218	0.418833574
0.001196588	0.414841738
0.001197293	0.413045823
0.00119887	0.410934945
0.001208283	0.401486016
0.001212771	0.397394732
0.001212771	0.396426566
0.001219728	0.389482987
0.001220788	0.388142118
0.001220956	0.387486862
0.001224377	0.383813076
0.001225528	0.382406915
0.001233778	0.375593314
0.001234394	0.374600902
0.001236735	0.372300127
0.001242702	0.365694859
0.001243089	0.364880322
0.00124439	0.363281217
0.00124439	0.362184038
0.00124439	0.362167926

0.001247595	0.358357619
0.001254037	0.352490182
0.001256565	0.349964436
0.00127634	0.334410847
0.001278921	0.331998749
0.001286105	0.325712423
0.001288428	0.32350338
0.001292384	0.318737331
0.001292384	0.318560576
0.001306072	0.307656104
0.001312117	0.302813587
0.001313621	0.30107905
0.00132667	0.290934276
0.001327104	0.290112834
0.00132998	0.287570033
0.001334871	0.283616731
0.001338303	0.280697495
0.001351173	0.270706027
0.00135305	0.267866699
0.001356502	0.262920667
0.001379961	0.244822006
0.001392616	0.234745075
0.001395476	0.231949999
0.001395476	0.231757647
0.001400652	0.227323488
0.001402191	0.225786902
0.001405307	0.223201277
0.001407361	0.221326727
0.001414926	0.214787667
0.001428142	0.20557756
0.001443768	0.193369268
0.001448043	0.190097004
0.001464068	0.177276686
0.001466902	0.174435015
0.001485872	0.159925973
0.001528375	0.131033464
0.001532199	0.127696481
0.001535172	0.124884914
0.001547298	0.117025929
0.001554117	0.111418288
0.001559921	0.106936637
0.001564941	0.103434684
0.00157154	0.098505721
0.001584196	0.090011425
0.001585124	0.088968074
0.001602807	0.0776026
0.001605833	0.074849172
0.001608121	0.07252986
0.001614247	0.068480549
0.001632283	0.055626904
0.001648933	0.04466215
0.0016579	0.038609735
0.001662633	0.035453913

0.001680045	0.025224019
0.001681465	0.023944238
0.001686262	0.020301892
0.001694427	0.015297156
0.001712725	0.001323458
0.001744048	-0.018281324
0.001744048	-0.018502566
0.001751204	-0.022814048
0.001782798	-0.042056148
0.001811858	-0.059948495
0.001853815	-0.085182309
0.00185776	-0.088094927
0.001864749	-0.092833685
0.001873092	-0.098603122
0.001875059	-0.100613539
0.001878533	-0.102820008
0.001878868	-0.103580382
0.001878868	-0.103940634
0.001882007	-0.107405485
0.00189327	-0.11487914
0.001896432	-0.117386743
0.001897776	-0.118522505
0.001899885	-0.120033586
0.001909974	-0.125452536
0.001912194	-0.127010582
0.001913031	-0.127892976
0.001924159	-0.134253749
0.001934108	-0.139541244
0.001946993	-0.148099215
0.001947568	-0.14899526
0.001955669	-0.153662409
0.001965718	-0.159856891
0.001967087	-0.161090682
0.001967087	-0.161445566
0.002025432	-0.192004117
0.002029987	-0.195037347
0.002045391	-0.20395485
0.002069706	-0.217773032
0.002105976	-0.238151571
0.002107007	-0.239994712
0.002128153	-0.251154383
0.002145342	-0.259944126
0.002145342	-0.260038669
0.002155438	-0.264877339
0.002175212	-0.275689889
0.002175682	-0.276349898
0.002189165	-0.284072438
0.002206489	-0.293120696
0.00222287	-0.300473249
0.002224466	-0.301598424
0.002226022	-0.303162351
0.002245815	-0.31278077
0.002252873	-0.317069873

0.002262483	-0.321640201
0.002265999	-0.323846723
0.002318918	-0.351188272
0.002320916	-0.35249259
0.002322373	-0.354034913
0.002322373	-0.354353519
0.002336352	-0.360469458
0.002348736	-0.365899734
0.002359345	-0.371391035
0.00236795	-0.375679725
0.0023835	-0.38535581
0.002415947	-0.401515778
0.002421791	-0.404210828
0.002465755	-0.424072298
0.002481927	-0.431045619
0.002492586	-0.435478414
0.002510018	-0.443293446
0.002511126	-0.445028063
0.002513989	-0.446859534
0.002522135	-0.450422607
0.002538792	-0.459188944
0.00255918	-0.468389357
0.002570129	-0.473239397
0.002572538	-0.474549034
0.002581753	-0.478751858
0.002587516	-0.481264225
0.002588269	-0.481971802
0.002590806	-0.483320298
0.002629312	-0.498359706
0.002644968	-0.506060629
0.002647031	-0.507220692
0.002665345	-0.516239596
0.002672736	-0.519249422
0.002722978	-0.539859768
0.002722978	-0.540034288
0.002728621	-0.542822277
0.002732651	-0.544626129
0.0027528	-0.553181001
0.002766487	-0.558653806
0.00277046	-0.560417752
0.002795058	-0.569497685
0.0028184	-0.578945734
0.002853747	-0.591818699
0.002888673	-0.604836765
0.002899295	-0.609527874
0.002907681	-0.612639008
0.00292481	-0.618950406
0.002929154	-0.620755021
0.002937765	-0.623908805
0.002948649	-0.629044228
0.002961899	-0.634586037
0.002962161	-0.63541184
0.002973662	-0.641126447

0.002995014	-0.648619842
0.003013586	-0.656470792
0.003038236	-0.666133266
0.003083314	-0.682340758
0.003083538	-0.683244706
0.003090849	-0.686280739
0.003092807	-0.687286632
0.003133036	-0.701377292
0.003146348	-0.70666426
0.003146348	-0.706980191
0.003149506	-0.708743322
0.003170998	-0.716182145
0.003171571	-0.717119262
0.003171571	-0.71798444
0.003191933	-0.72448797
0.003193907	-0.725768258
0.003199126	-0.728489017
0.003199126	-0.729301057
0.003199126	-0.731263559
0.003199126	-0.73137782
0.003199126	-0.731531244
0.003216524	-0.737964748
0.003216838	-0.738713943
0.003219444	-0.739876371
0.003235576	-0.746521129
0.003257925	-0.754595569
0.003264435	-0.757502869
0.003276456	-0.761494979
0.003291621	-0.76618959
0.003299727	-0.769288137
0.00330981	-0.774066079
0.003317467	-0.776707144
0.003322066	-0.7783993
0.003325279	-0.779702532
0.003325504	-0.780171463
0.003326269	-0.780860946
0.003326269	-0.781196279
0.003339588	-0.785715788
0.003348533	-0.78860358
0.003354621	-0.790694287
0.003376693	-0.797777146
0.00337696	-0.798971599
0.003388725	-0.802909734
0.003394589	-0.804917571
0.00340077	-0.807008881
0.003402255	-0.807816903
0.003412483	-0.811810377
0.003432964	-0.819089293
0.003432964	-0.819372068
0.003436097	-0.82102105
0.003449058	-0.825716609
0.003456038	-0.828417029
0.003456038	-0.828793176

0.003508475	-0.844361088
0.003511268	-0.845886245
0.003513013	-0.846757119
0.003536482	-0.855322389
0.003544613	-0.858247516
0.003552699	-0.861948857
0.003568888	-0.866561851
0.003642581	-0.888284637
0.003644085	-0.889062608
0.00364523	-0.889748873
0.003661417	-0.89543483
0.0036708	-0.89820179
0.003679986	-0.900912515
0.00368633	-0.904083301
0.003686598	-0.904543791
0.003691862	-0.906259352
0.00371802	-0.913589269
0.003733583	-0.920203895
0.003743876	-0.923537584
0.003749346	-0.925281589
0.003753399	-0.926673525
0.0037603	-0.928766624
0.003778115	-0.933927113
0.003779546	-0.9346681
0.00379166	-0.938413077
0.003808144	-0.943600742
0.003852428	-0.955091241
0.003879439	-0.964280886
0.003898839	-0.970063738
0.003901505	-0.971083897
0.003914215	-0.974869919
0.003937178	-0.981202299
0.003937178	-0.981446959
0.003972185	-0.990802956
0.004001801	-0.99999203
0.004033249	-1.008393416
0.004072643	-1.019694179
0.004086515	-1.023607969
0.00409785	-1.02693729
0.004103779	-1.029052544
0.004103779	-1.029336532
0.004106449	-1.030405735
0.004128923	-1.035840172
0.004131379	-1.036771811
0.004142339	-1.040748217
0.00415633	-1.044664
0.00415633	-1.045201439
0.00415633	-1.045390665
0.004177032	-1.053402843
0.004229969	-1.06770417
0.00425557	-1.074042439
0.004267681	-1.077049174
0.004270193	-1.078347207

0.004273684	-1.079479925
0.004295167	-1.085248006
0.004303965	-1.087517144
0.004306304	-1.088395645
0.004313697	-1.090733574
0.004317639	-1.092261971
0.004321444	-1.093518803
0.004330333	-1.095794653
0.004354364	-1.102037741
0.004365854	-1.104849585
0.004397731	-1.11270036
0.004407486	-1.116242953
0.004430322	-1.122380335
0.004430322	-1.122442721
0.004430322	-1.122885439
0.004437096	-1.124670514
0.004450657	-1.128949294
0.004450657	-1.128979449
0.004457321	-1.131476634
0.004492341	-1.140262165
0.004506259	-1.1445367
0.004509834	-1.145706949
0.004509834	-1.146008924
0.004514419	-1.148279023
0.004514419	-1.148514568
0.004518109	-1.150037194
0.004518109	-1.15028625
0.004533667	-1.154936691
0.004538941	-1.156814842
0.004538941	-1.157384094
0.004538941	-1.157848345
0.004552967	-1.161801024
0.004561781	-1.163955544
0.004614476	-1.176036638
0.004640098	-1.182251189
0.004710596	-1.199468226
0.00475367	-1.208970299
0.004764036	-1.212072675
0.00476672	-1.212956029
0.004769686	-1.213991362
0.004777956	-1.216581726
0.004799874	-1.22189628
0.004814894	-1.227172342
0.004814894	-1.228033579
0.004841996	-1.233939376
0.004847612	-1.235370377
0.004880504	-1.242695964
0.004888822	-1.244987921
0.004907839	-1.248932813
0.004923348	-1.252564718
0.004995696	-1.269260018
0.005005604	-1.27145843
0.005027304	-1.276512671

0.005042587	-1.280028774
0.005048683	-1.281901091
0.005062959	-1.285532827
0.00508569	-1.290862357
0.00508569	-1.291279316
0.005089235	-1.292445066
0.005099934	-1.294738571
0.005106893	-1.297061812
0.00511655	-1.299160014
0.005124902	-1.301019911
0.005145833	-1.305844113
0.005212789	-1.319413377
0.005248183	-1.327550572
0.005265703	-1.331682319
0.005275102	-1.334031547
0.005280486	-1.335324438
0.005286193	-1.338470729
0.005286193	-1.338779092
0.005328673	-1.347914404
0.005332087	-1.349204891
0.005358443	-1.354103001
0.005362767	-1.355545811
0.005385924	-1.360916115
0.005391004	-1.362134321
0.005404846	-1.364847874
0.005412619	-1.366521679
0.005426656	-1.36925868
0.005443836	-1.373565215
0.005448651	-1.374728014
0.00545882	-1.377142198
0.005502361	-1.38620236
0.005508054	-1.387650789
0.005508054	-1.38819291
0.005508054	-1.389318177
0.00551047	-1.390328101
0.005514397	-1.391330391
0.005522111	-1.393134221
0.005561874	-1.401478811
0.005600229	-1.409015967
0.005600295	-1.409370715
0.005622509	-1.413363537
0.005662733	-1.420964705
0.005662952	-1.421343577
0.005695614	-1.427330083
0.005706541	-1.429439123
0.005761937	-1.439903214
0.005761937	-1.440053647
0.005764999	-1.440885281
0.005791125	-1.446759511
0.005801912	-1.449025282
0.005801912	-1.449156319
0.005809059	-1.450972403
0.005827126	-1.455535485

0.005841065	-1.458416447
0.005898507	-1.469206876
0.005898507	-1.469467972
0.005903176	-1.471590304
0.00594823	-1.479570002
0.005970244	-1.483985952
0.005994043	-1.488788804
0.005998039	-1.490287661
0.006020963	-1.495147174
0.006033277	-1.497833876
0.006033277	-1.498333957
0.006042523	-1.500455007
0.00605455	-1.502621478
0.006055661	-1.503125975
0.006059038	-1.504309882
0.006079425	-1.508072684
0.006098317	-1.511598935
0.006115817	-1.516945783
0.006115817	-1.5172215
0.006115817	-1.517286645
0.006115817	-1.517577479
0.006116039	-1.517944275
0.006145641	-1.523721195
0.006153908	-1.525291273
0.006225578	-1.53727859
0.006250376	-1.541601226
0.006302781	-1.551935823
0.006327733	-1.557687753
0.006327733	-1.558207893
0.006327733	-1.558214658
0.0063332	-1.559339084
0.006333795	-1.559755167
0.006335431	-1.560452239
0.00637449	-1.566965457
0.0063852	-1.568838964
0.006406995	-1.573229279
0.006429986	-1.57691258
0.006436005	-1.578101651
0.006482183	-1.585662233
0.006504059	-1.589272409
0.006508628	-1.591466903
0.006508628	-1.591617275
0.006508628	-1.591823186
0.006508628	-1.592106737
0.006508628	-1.592232316
0.006510709	-1.593180841
0.006510709	-1.593300656
0.006517325	-1.594561311
0.006610429	-1.609887786
0.006611089	-1.610483086
0.006611089	-1.610630519
0.006638061	-1.615349802
0.006649889	-1.617636347

0.006680137	-1.622133532
0.006747541	-1.632012121
0.006751851	-1.632923328
0.006773422	-1.636811233
0.006780301	-1.63808448
0.006806845	-1.642969864
0.006834748	-1.648022049
0.006863307	-1.653144434
0.006873879	-1.654903481
0.006889827	-1.657976996
0.006897588	-1.659976502
0.006917409	-1.663256168
0.00691807	-1.663855901
0.00691807	-1.663986302
0.006942608	-1.667560549
0.006957271	-1.669819885
0.006992396	-1.675408679
0.00702225	-1.680700375
0.007089584	-1.691925808
0.007131615	-1.698311538
0.007147727	-1.700702789
0.007152303	-1.701609063
0.007155344	-1.702954402
0.007207018	-1.711151653
0.007210536	-1.711917166
0.007251526	-1.718074078
0.007275383	-1.721406979
0.00728116	-1.722769343
0.007323474	-1.728406132
0.007336445	-1.730347281
0.00740941	-1.741012864
0.00743398	-1.745833049
0.00743398	-1.746467859
0.00743398	-1.746540295
0.00743398	-1.747204675
0.007445711	-1.749281207
0.007492917	-1.755396068
0.007499191	-1.756792875
0.00752183	-1.760501056
0.007618304	-1.774076226
0.007635046	-1.776403373
0.007705511	-1.787332036
0.007778035	-1.800175597
0.007860083	-1.811920305
0.007860083	-1.81207901
0.007876216	-1.814268947
0.007893957	-1.816641807
0.007896405	-1.81723563
0.007916393	-1.819863138
0.007968213	-1.828008279
0.008007945	-1.833375039
0.008071433	-1.84324598
0.008073236	-1.84524246

0.008078142	-1.84641257
0.008081881	-1.847143259
0.008104046	-1.850572436
0.008212769	-1.864009241
0.008217178	-1.865001749
0.008217178	-1.865112162
0.008244078	-1.869152271
0.008244078	-1.869327986
0.008271745	-1.873007659
0.008322742	-1.879369489
0.008322742	-1.879552823
0.008362488	-1.88447325
0.008362488	-1.884830395
0.008402791	-1.889842932
0.008410705	-1.891009087
0.008474494	-1.89975082
0.008487841	-1.901495237
0.008512482	-1.905058928
0.008524475	-1.906651034
0.008557131	-1.910809009
0.00856076	-1.91175065
0.008647029	-1.922139969
0.008647537	-1.922495457
0.008677437	-1.925957436
0.00868529	-1.927087117
0.008742703	-1.934830079
0.008768711	-1.937945761
0.008775904	-1.938997018
0.008850925	-1.947389598
0.008894706	-1.952806321
0.008911352	-1.956016519
0.008991308	-1.966882283
0.008998809	-1.968540729
0.009038177	-1.972833081
0.009186626	-1.990119049
0.00921829	-1.993860854
0.009318988	-2.005578287
0.009327517	-2.007181319
0.009333382	-2.008175007
0.009408553	-2.016398057
0.009427154	-2.018795275
0.009433275	-2.020270558
0.009494512	-2.028844652
0.009536332	-2.03403921
0.00957052	-2.03760429
0.00957692	-2.038509197
0.00959446	-2.041466826
0.00959446	-2.041472645
0.009626663	-2.045269946
0.009626663	-2.04529861
0.009632509	-2.046603967
0.009660245	-2.04994555
0.009699226	-2.054503204

0.009718852	-2.056642712
0.009722509	-2.057278637
0.009729606	-2.058821335
0.009743232	-2.060889792
0.009772841	-2.064039234
0.009786103	-2.065861076
0.009840203	-2.072398727
0.009844961	-2.073666454
0.009889108	-2.07833454
0.009894423	-2.079115577
0.009932293	-2.084344428
0.010124789	-2.103610945
0.010175776	-2.109064076
0.01023056	-2.114255082
0.01032262	-2.123298632
0.010351493	-2.126424641
0.010358463	-2.127394052
0.010358463	-2.127614943
0.0103599	-2.128303584
0.010376076	-2.130028868
0.010408111	-2.133704792
0.010510699	-2.144378274
0.010513758	-2.145217078
0.010585474	-2.152559709
0.010585474	-2.152565512
0.010618639	-2.155705848
0.010705466	-2.164278937
0.010718649	-2.1656872
0.010753157	-2.169190231
0.010782019	-2.172205827
0.010799238	-2.173946529
0.010809042	-2.175059219
0.010839513	-2.17791175
0.010855879	-2.179857291
0.010856589	-2.180201567
0.010923557	-2.186734114
0.010923557	-2.187515649
0.010954434	-2.190941329
0.01095795	-2.191517679
0.010961753	-2.192684564
0.010995666	-2.195784644
0.010997256	-2.196287135
0.010997256	-2.196482835
0.011001722	-2.197136042
0.011028868	-2.200318706
0.011028868	-2.200514317
0.011038492	-2.201874668
0.011053839	-2.204270452
0.01108668	-2.20725708
0.011177148	-2.215792781
0.011209949	-2.219026906
0.011211667	-2.219447974
0.011275857	-2.225589584

0.011275857	-2.225775874
0.011286645	-2.226928261
0.011289189	-2.227414527
0.011299558	-2.228812539
0.011304999	-2.229812261
0.011489372	-2.246513828
0.011502668	-2.247847303
0.011520487	-2.249575321
0.011520487	-2.249816583
0.011554196	-2.253595504
0.011590217	-2.256989036
0.011594708	-2.257899644
0.011600025	-2.258595921
0.011710973	-2.268380079
0.011757175	-2.272451105
0.011758352	-2.273015335
0.011789126	-2.276017643
0.011792606	-2.277210965
0.011792606	-2.277535045
0.011792606	-2.278066007
0.011802102	-2.27996322
0.011802102	-2.280156936
0.01186213	-2.286435475
0.011925823	-2.293518045
0.011927644	-2.294102511
0.011927644	-2.294207983
0.012027025	-2.302862042
0.012046656	-2.304882832
0.012153736	-2.314049267
0.012183298	-2.31763139
0.01227427	-2.326590081
0.012303018	-2.329220033
0.012303018	-2.329321736
0.012308477	-2.330305276
0.012335666	-2.333313655
0.01234062	-2.334224051
0.01235856	-2.335818057
0.01236859	-2.336828439
0.012405709	-2.3406429
0.012426299	-2.342695198
0.012459986	-2.34624418
0.012486698	-2.348463458
0.012586031	-2.356483576
0.012605455	-2.358969605
0.012653486	-2.362698736
0.012675945	-2.365393149
0.01267629	-2.365688158
0.012713622	-2.368902278
0.012737766	-2.371177039
0.012737766	-2.37170686
0.012784916	-2.375335657
0.012800016	-2.376947853
0.012994261	-2.392253695

0.01311608	-2.401540223
0.013125685	-2.402742506
0.013127125	-2.403110588
0.013146452	-2.404715806
0.013147205	-2.40503613
0.01325822	-2.414016151
0.01325855	-2.414306489
0.013263056	-2.414882868
0.013271514	-2.415997053
0.013302981	-2.418415953
0.013314379	-2.419728575
0.013357164	-2.423444273
0.013392364	-2.426368586
0.013394112	-2.426753903
0.013404946	-2.428021531
0.013441729	-2.431575908
0.013457768	-2.432925165
0.013486158	-2.435637048
0.013520134	-2.438187982
0.013545477	-2.440685656
0.013564011	-2.442192842
0.013583728	-2.443777311
0.013637025	-2.44759839
0.013665583	-2.450028297
0.013673573	-2.451226124
0.013673573	-2.451353901
0.013673807	-2.451634261
0.013678691	-2.452487922
0.013694491	-2.454329546
0.013710517	-2.457073153
0.013738389	-2.459611351
0.013844263	-2.468423701
0.013854498	-2.46962051
0.013861434	-2.47060052
0.013885966	-2.473255886
0.013955835	-2.479341638
0.013955835	-2.479439485
0.013955835	-2.479646172
0.014029068	-2.485704066
0.014034795	-2.486703244
0.014034795	-2.487119798
0.014035388	-2.487419241
0.014043484	-2.488464611
0.014085397	-2.492211679
0.014119658	-2.494676417
0.014186613	-2.499489191
0.014208263	-2.501132885
0.014226463	-2.502814578
0.014319473	-2.510025213
0.014328171	-2.510835676
0.01437967	-2.514349332
0.014386715	-2.515803228
0.014386715	-2.515989307

0.014440868	-2.519756755
0.014443014	-2.520157934
0.014443014	-2.520409504
0.014445243	-2.520808404
0.014492445	-2.524383973
0.014492445	-2.524542926
0.014504819	-2.525575365
0.014548886	-2.529099535
0.014548886	-2.529108103
0.014621879	-2.534671206
0.014651898	-2.537046368
0.014695627	-2.54000574
0.014735204	-2.54295908
0.014747432	-2.544226022
0.014809692	-2.548816559
0.014838558	-2.550838575
0.014871492	-2.553618909
0.014942555	-2.558195943
0.014969528	-2.560600247
0.015039143	-2.566086237
0.015039932	-2.566389896
0.01508853	-2.570194511
0.015116635	-2.572789665
0.015146375	-2.574825827
0.015182347	-2.577485539
0.015205228	-2.579360263
0.015216835	-2.580306651
0.015224631	-2.581277815
0.015224631	-2.581535572
0.015325312	-2.588524558
0.015329963	-2.589053908
0.015380309	-2.592532179
0.015421523	-2.596642387
0.015421523	-2.596987869
0.015473723	-2.60055515
0.015484964	-2.601466215
0.015513687	-2.603397697
0.015517665	-2.603883182
0.015651533	-2.613202146
0.015651533	-2.613431684
0.015656389	-2.613965242
0.015668843	-2.6149378
0.015679415	-2.615801009
0.015733716	-2.620192447
0.01578533	-2.623409104
0.015839445	-2.627262607
0.01585886	-2.628623089
0.015865566	-2.629257501
0.015895379	-2.631459832
0.015942042	-2.634364109
0.016029189	-2.641808513
0.016189562	-2.651851131
0.016189562	-2.652016462

0.016206712	-2.653526588
0.016254762	-2.657857451
0.016254762	-2.657954407
0.016300277	-2.661232395
0.016421953	-2.669544922
0.016421953	-2.669703071
0.016441185	-2.671529945
0.016463777	-2.674489556
0.016518081	-2.678955487
0.016760217	-2.694586003
0.016803899	-2.698980163
0.016808729	-2.699715259
0.016825834	-2.701077828
0.016880808	-2.704272002
0.017020293	-2.713183807
0.017111472	-2.71874792
0.017143509	-2.721174056
0.017152616	-2.723124376
0.017314945	-2.733100831
0.017357879	-2.735826312
0.01740421	-2.738478009
0.017495392	-2.744417081
0.017539093	-2.746913583
0.017589349	-2.750715188
0.017591336	-2.75120027
0.017591336	-2.751303902
0.017605594	-2.752521866
0.017702777	-2.759677304
0.017757217	-2.763176378
0.017849187	-2.768596228
0.01789128	-2.771659753
0.018019736	-2.779082806
0.018067239	-2.781699947
0.018119789	-2.784561998
0.01814014	-2.785816182
0.018194057	-2.788735144
0.018269936	-2.793454209
0.018292766	-2.794821699
0.018417508	-2.802396009
0.018526343	-2.808190761
0.018728075	-2.8196372
0.018758712	-2.82159042
0.018765533	-2.822157825
0.018941585	-2.832490527
0.018990738	-2.835543404
0.019024019	-2.837360399
0.019033521	-2.838049173
0.019064595	-2.839758097
0.019194207	-2.846818193
0.019377822	-2.857494192
0.019389	-2.858771433
0.019389887	-2.859117025
0.019452711	-2.86291359

0.0194601	-2.863492876
0.019543846	-2.868776271
0.019543846	-2.868782584
0.019594045	-2.872984804
0.01961796	-2.875358185
0.019631704	-2.876601724
0.019687294	-2.879619429
0.019736928	-2.882557446
0.019736928	-2.882593228
0.019754842	-2.883880727
0.019759325	-2.884320239
0.019796869	-2.88720446
0.019827533	-2.888832827
0.0198932	-2.892044413
0.019931403	-2.894240908
0.02005229	-2.90038763
0.020151584	-2.905536388
0.020504391	-2.923725995
0.020567226	-2.92708631
0.020620843	-2.930041424
0.020620843	-2.930452929
0.020629536	-2.931064963
0.020644015	-2.931928913
0.020798495	-2.940030096
0.020847515	-2.94284419
0.020854434	-2.943374782
0.020895147	-2.945360831
0.020903367	-2.946314007
0.021033091	-2.952491016
0.021234886	-2.964721008
0.021298544	-2.968794811
0.021446161	-2.9765244
0.021481057	-2.978533893
0.02162561	-2.985478881
0.021660589	-2.987733673
0.021660589	-2.987848455
0.021753945	-2.992178155
0.021753945	-2.992399639
0.021787388	-2.994008642
0.021799561	-2.994968299
0.021808232	-2.99555435
0.021910709	-3.000583974
0.021910709	-3.000679991
0.021922328	-3.001384682
0.0220747	-3.008518808
0.022142252	-3.011735853
0.022142252	-3.012174994
0.022235865	-3.016874567
0.022331785	-3.021194516
0.022609857	-3.035782418
0.022609857	-3.03593591
0.022619282	-3.036536515
0.02266026	-3.038613814

0.0227521	-3.042469875
0.022811014	-3.045469052
0.022831888	-3.04674192
0.022835855	-3.047133891
0.022835855	-3.047349723
0.022975726	-3.054839659
0.023011067	-3.056913411
0.023024374	-3.057657136
0.023110561	-3.062528342
0.023110561	-3.062582853
0.023199485	-3.066925529
0.023264219	-3.070321771
0.023334072	-3.074130516
0.023428644	-3.078205007
0.023453948	-3.079591019
0.023548092	-3.083435454
0.023573774	-3.084859691
0.023584485	-3.085610693
0.023584485	-3.085767969
0.02369111	-3.091319147
0.023794673	-3.095897195
0.023807895	-3.096617795
0.023825179	-3.097754362
0.023872475	-3.100380455
0.024063704	-3.108558067
0.024109968	-3.110699854
0.024152511	-3.113050401
0.024176203	-3.114150443
0.024260009	-3.118136576
0.024290669	-3.119790033
0.024290669	-3.11993193
0.024301003	-3.120534125
0.024355511	-3.123063879
0.024355511	-3.123646141
0.02443897	-3.128034223
0.024459358	-3.129438009
0.024459358	-3.129442392
0.02455666	-3.133272087
0.02455666	-3.133438435
0.024676319	-3.138231387
0.024805995	-3.144902576
0.024994834	-3.152349755
0.025057453	-3.155247173
0.025071691	-3.155974798
0.025135472	-3.158905835
0.025154105	-3.159802119
0.025154105	-3.160006837
0.025181794	-3.161647695
0.025430733	-3.171655914
0.025430733	-3.172197289
0.025470667	-3.174502233
0.025470667	-3.174691396
0.025492703	-3.175682938

0.025572169	-3.179120427
0.025583379	-3.179947755
0.025617368	-3.181352878
0.025717209	-3.185484311
0.025833499	-3.189953791
0.025841903	-3.190461863
0.025847898	-3.191371396
0.025913334	-3.194449004
0.026040036	-3.19967673
0.026136774	-3.204290014
0.026208821	-3.207183085
0.026284229	-3.210830897
0.026290028	-3.211264176
0.026290028	-3.211640258
0.026441957	-3.217053712
0.026512604	-3.221363128
0.026613934	-3.22498681
0.026651039	-3.226446626
0.026686737	-3.228287147
0.026723359	-3.229941765
0.026784296	-3.232405842
0.026808034	-3.233519759
0.026869581	-3.236103461
0.026878561	-3.236691369
0.026905984	-3.238343102
0.026977066	-3.241168326
0.027019084	-3.242772267
0.027112708	-3.246716583
0.027206778	-3.250023172
0.027283319	-3.253172243
0.027339639	-3.255440152
0.02777896	-3.271937778
0.02777896	-3.272013053
0.027812354	-3.273297867
0.027824875	-3.274124635
0.028041116	-3.281669876
0.028122046	-3.284887477
0.028122046	-3.285317735
0.028122046	-3.28538604
0.028128242	-3.285922502
0.028136984	-3.286623441
0.028283771	-3.292323622
0.028298837	-3.293010152
0.028345364	-3.295743394
0.028362523	-3.296494299
0.02838702	-3.297686425
0.028400594	-3.298323781
0.028504942	-3.302437891
0.028557289	-3.304495411
0.028614213	-3.30690275
0.028718041	-3.310973649
0.028782006	-3.314217725
0.028890037	-3.318187086

0.028924875	-3.31962285
0.029000138	-3.322625789
0.029207442	-3.330230003
0.029453468	-3.338540483
0.029639526	-3.346236868
0.02974615	-3.35031607
0.02974615	-3.350476653
0.029779804	-3.35169186
0.02993774	-3.356818673
0.030128781	-3.363521895
0.03018883	-3.365502304
0.03019375	-3.365854963
0.030227128	-3.367163406
0.030227128	-3.367253624
0.030326043	-3.370991462
0.030670786	-3.3827087
0.030686141	-3.383360854
0.030703904	-3.3844958
0.030802204	-3.388238816
0.030802204	-3.388372364
0.030803806	-3.388741197
0.030837881	-3.390433271
0.030913923	-3.393036542
0.031030104	-3.396991394
0.031042087	-3.397540621
0.031044597	-3.398084516
0.031044597	-3.398229546
0.031112772	-3.401143942
0.031135281	-3.402127652
0.031223308	-3.405388627
0.031227019	-3.405699379
0.031230349	-3.406204163
0.031535368	-3.41587126
0.031757313	-3.4239462
0.031846229	-3.426635753
0.031860885	-3.427248847
0.032219767	-3.439241657
0.032271557	-3.441075737
0.032290966	-3.441813372
0.032295399	-3.442138729
0.032423215	-3.446867112
0.032482113	-3.449088266
0.032533556	-3.450899677
0.032589418	-3.452626384
0.032625747	-3.454467274
0.032712456	-3.457391177
0.032827486	-3.46149367
0.032827486	-3.461519803
0.032874335	-3.463233904
0.033043888	-3.468969396
0.033246504	-3.475807112
0.033296519	-3.47734283
0.033441506	-3.481674968

0.033574032	-3.487522666
0.033582019	-3.487979674
0.033582019	-3.488134843
0.03374432	-3.493615096
0.033752629	-3.494033715
0.033808164	-3.495892531
0.034054069	-3.503121462
0.034100793	-3.504537459
0.034229764	-3.509482217
0.034268648	-3.510887792
0.034337321	-3.512959541
0.034374685	-3.514225194
0.034518784	-3.518133616
0.034537039	-3.518801442
0.034670919	-3.522828445
0.034715306	-3.524360316
0.034742068	-3.52544087
0.034775747	-3.526697054
0.034801494	-3.527551662
0.034814979	-3.528490418
0.034816443	-3.528726079
0.034822548	-3.529079796
0.034884983	-3.531528404
0.034884983	-3.532066477
0.035167797	-3.541384643
0.035689253	-3.556789217
0.035979495	-3.565731335
0.036150003	-3.571290742
0.036182883	-3.572746524
0.036182883	-3.572879366
0.036211948	-3.573786021
0.03627283	-3.57546886
0.036717259	-3.588201856
0.036782775	-3.589974548
0.036826104	-3.59160162
0.036894786	-3.594030034
0.036957481	-3.596700528
0.03695766	-3.596899408
0.036983706	-3.597762582
0.037030742	-3.600033259
0.037228074	-3.606260957
0.037315886	-3.609311538
0.037320826	-3.609817493
0.037349783	-3.610997014
0.037426986	-3.613103058
0.037482448	-3.614605703
0.037516544	-3.615953807
0.037516544	-3.616467895
0.037563565	-3.618254982
0.037579322	-3.618818647
0.037692111	-3.621660834
0.037771808	-3.623913454
0.037941931	-3.629040851

0.038047351	-3.631893362
0.038114523	-3.634056379
0.038114523	-3.634205438
0.038190596	-3.63615947
0.038196832	-3.636644368
0.03819866	-3.6369221
0.038210711	-3.637392773
0.038212667	-3.637629887
0.038261663	-3.63895392
0.038359233	-3.641779322
0.038670568	-3.650643175
0.038682555	-3.651299341
0.038817513	-3.654795098
0.039042497	-3.661959712
0.039063798	-3.662822338
0.039452511	-3.672514975
0.039502378	-3.673820098
0.03951198	-3.674414887
0.039541633	-3.67526706
0.039810769	-3.682961694
0.039862947	-3.684306986
0.039936189	-3.686684561
0.039950422	-3.68718834
0.040006065	-3.688984132
0.040098289	-3.69120461
0.040222577	-3.695068774
0.040443973	-3.70073163
0.040443973	-3.700788704
0.040835048	-3.711614045
0.040835048	-3.711975936
0.041074896	-3.718453459
0.041159988	-3.721139513
0.041159988	-3.721215518
0.041162593	-3.721492394
0.041162593	-3.721646243
0.04120869	-3.722820028
0.041277482	-3.724851863
0.041356977	-3.726734515
0.041370653	-3.727400179
0.041463343	-3.729746214
0.041514572	-3.731395
0.041589203	-3.733724602
0.041596426	-3.734064287
0.041608472	-3.734662751
0.041764263	-3.738171312
0.04182785	-3.73988435
0.041897885	-3.741730662
0.041996945	-3.744450513
0.042155169	-3.748983371
0.042202698	-3.750347339
0.042351656	-3.754192475
0.042630767	-3.761014616
0.042630767	-3.761157139

0.042842228	-3.767109563
0.042848528	-3.767608874
0.043131719	-3.774513905
0.043207221	-3.776619129
0.043379688	-3.780848505
0.043394858	-3.781524643
0.043456049	-3.782948334
0.043572023	-3.785660807
0.043578327	-3.786326571
0.043597565	-3.786912325
0.043674975	-3.788840246
0.043766874	-3.791056289
0.043824315	-3.792393343
0.044085769	-3.798541217
0.04415407	-3.800085623
0.044203118	-3.801795048
0.044476777	-3.807769458
0.044717224	-3.812873683
0.044719661	-3.813104586
0.044773173	-3.814433657
0.044977283	-3.819043736
0.045106575	-3.82213583
0.045206133	-3.824437683
0.045224629	-3.824979512
0.045284525	-3.826506943
0.045400609	-3.829393013
0.045400609	-3.829484781
0.045707953	-3.836865899
0.04592608	-3.841409371
0.046153481	-3.84724521
0.046153481	-3.847379488
0.046276607	-3.85008052
0.046288172	-3.850481116
0.046405272	-3.853282513
0.046431944	-3.854108548
0.046432016	-3.854290978
0.046873308	-3.863675518
0.046901697	-3.864534522
0.046901697	-3.864568087
0.047480141	-3.878023444
0.047720842	-3.883536205
0.047842419	-3.886485436
0.047926299	-3.888379998
0.047988637	-3.889976192
0.048360847	-3.899560216
0.048360847	-3.899693592
0.048406683	-3.90088104
0.048644758	-3.906071884
0.048709561	-3.907594862
0.048728793	-3.908118982
0.048867447	-3.910785927
0.048873854	-3.911079037
0.04894112	-3.912639982

0.049019092	-3.914711667
0.049053398	-3.915537064
0.049225594	-3.919316973
0.049236543	-3.920105501
0.049236543	-3.92075545
0.049246401	-3.921108249
0.049297362	-3.922546084
0.049343058	-3.923811417
0.049522398	-3.927950796
0.049585189	-3.929413529
0.049585189	-3.929413911
0.049985155	-3.93768231
