# Supplementary material for: Weighted Gene Correlation Network Analysis Identifies Specific Functional Modules and Genes in Esophageal Cancer
Source: J Oncol. 2021 Dec 27;2021:8223263. doi: 10.1155/2021/8223263 (PMC8723838; doi:10.1155/2021/8223263)
Supplement: Supplementary Materials — Supplementary Table 1. The detailed information of DEGs between ESCA tumors and normal tissues in TCGA cohort. Supplementary Table 2. The detailed information of DEGs between ESCA tumors and normal tissues in the GSE38129 dataset. . [file 8223263.f1.zip › 8223263.f1/Supplementary table 2.pdf]

Supplementary table 2. The detailed information of DEGs between ESCA tumors and normal tissues in the GSE38129 dataset.

ID	logFC	AveExpr	t	P.Value
CBX3	1.331228899	9.958653025	14.03779012	1.21E-20
UBL3	-2.030601532	8.578042781	-11.93958966	1.63E-17
KAT2B	-2.563703751	7.867239397	-11.58020464	5.93E-17
KIF4A	2.148217187	6.449535255	11.18479926	2.50E-16
AURKA	1.443287248	6.087479091	11.16957811	2.64E-16
LMBRD1	-1.122055508	9.213949725	-11.04764967	4.13E-16
MGLL	-2.611752339	8.893340468	-10.74752476	1.25E-15
CKS1B	1.712048368	9.298659768	10.68949203	1.55E-15
ECT2	2.377331671	8.231403615	10.56514512	2.47E-15
ATAD2	2.064620715	6.39610046	10.52121921	2.91E-15
MTHFD2	1.691287016	9.471645411	10.44043819	3.93E-15
RFC4	1.891903453	7.924993867	10.43255127	4.05E-15
HOXB7	1.951657231	6.759270724	10.36584245	5.20E-15
KIF14	2.030790078	6.351845982	10.35818041	5.35E-15
GPX3	-2.95508115	9.251858829	-10.31693413	6.25E-15
NUP107	1.177295036	8.505752244	10.22225198	8.93E-15
CRYL1	-1.511701519	7.378811852	-10.21788167	9.08E-15
COL10A1	3.060733685	5.781788467	10.10431869	1.39E-14
HMGB3	1.568979544	7.636900656	10.05154406	1.70E-14
TRIP13	2.071615013	7.456765596	9.999610834	2.07E-14
EPB41L3	-2.731519849	6.08888013	-9.99717496	2.09E-14
CITED2	-2.383849681	7.163797692	-9.971366591	2.31E-14
HSDL2	-1.103290626	6.667678223	-9.830806918	3.94E-14
MCM2	1.843571631	7.794251654	9.818282301	4.13E-14
WDHD1	1.251791032	4.73493224	9.757321546	5.21E-14
ANP32E	1.109652266	8.581373532	9.737427286	5.62E-14
RAD51AP1	1.999907677	6.946766731	9.727665722	5.83E-14
NCAPG2	1.314428319	6.268924231	9.714824449	6.13E-14
LAPTM4B	1.600733221	9.6398827	9.699786297	6.49E-14
MCM7	1.343222271	8.290675799	9.671179301	7.24E-14
GPD1L	-1.925523854	8.334032583	-9.667726024	7.33E-14
FANCI	1.698161892	7.015870553	9.623036026	8.70E-14
CPEB3	-1.837359279	5.585502305	-9.618662377	8.85E-14
ID4	-1.870596685	7.988699423	-9.615599181	8.95E-14
FCER1A	-2.336942913	5.77490647	-9.53496187	1.22E-13
COL11A1	3.884311814	6.064270499	9.493765322	1.43E-13
MAD2L1	1.849288198	7.693125957	9.491301325	1.44E-13
SOX4	1.522042503	7.737989362	9.489229678	1.45E-13
PHACTR2	-1.238560621	7.748320023	-9.434054336	1.80E-13
DNMT3B	1.57072225	6.251373842	9.429395558	1.83E-13
NDC80	1.77514691	6.820648358	9.40501013	2.01E-13
NEK2	1.310309463	5.438693873	9.401563757	2.04E-13
KIF2C	1.434240993	6.489793326	9.387672912	2.15E-13
MTERF3	1.111225813	8.589698008	9.37902462	2.22E-13
ETFDH	-1.553081314	6.382993197	-9.373112232	2.27E-13
HIGD1A	-1.096257414	10.26454098	-9.311089151	2.89E-13
KIF23	2.123586008	5.795187593	9.301554979	2.99E-13
FBXO3	-1.430927989	7.892802577	-9.29757497	3.04E-13
TFRC	1.937885927	10.49219965	9.242692649	3.76E-13

POLR2H	1.026190036	9.226265476	9.240284443	3.79E-13
KANK1	-1.779757308	8.759138586	-9.211616376	4.24E-13
DMXL1	-1.236036723	7.807989175	-9.199473174	4.44E-13
ABLIM3	-1.910183929	6.859519295	-9.164557608	5.08E-13
NUCB2	-1.99248837	9.320540237	-9.108416413	6.32E-13
UBE2C	1.964888916	8.462607958	9.097093262	6.60E-13
ACTL6A	1.386138729	9.107541247	9.096270518	6.62E-13
CDKN3	1.881868597	6.973633812	9.085009761	6.92E-13
KIF20A	1.830122998	6.862969558	9.071903997	7.28E-13
SPAG5	1.398466788	7.406635418	9.0626328	7.54E-13
ABLIM1	-1.758949662	8.98989519	-9.040081656	8.23E-13
PAIP2B	-1.598908272	6.628047599	-9.020560579	8.88E-13
GINS2	1.665380593	7.492074071	9.012617339	9.16E-13
FZD6	1.393668995	9.042498696	8.999663572	9.63E-13
CENPF	1.272963655	5.645921882	8.994379728	9.83E-13
PPP1R3C	-3.1315022	8.489420407	-8.988501481	1.01E-12
PINK1	-1.051417563	7.865421145	-8.974752617	1.06E-12
STMN1	1.156614513	7.87191318	8.958867144	1.13E-12
ARHGAP10	-1.086167194	7.028113773	-8.952186496	1.16E-12
EXO1	1.397239893	5.784787258	8.929088631	1.27E-12
HOXA10	1.654377361	5.801448885	8.921365635	1.31E-12
DNMT1	1.051677274	8.215343093	8.90892181	1.37E-12
YEATS2	1.143049407	7.180458893	8.859805297	1.66E-12
CRIP2	-1.735606937	7.760391264	-8.856266979	1.68E-12
CDK1	2.041154561	7.746036841	8.84145423	1.78E-12
NUP155	1.074701128	6.504719152	8.828450777	1.87E-12
TBC1D31	1.076212509	5.882867963	8.81767237	1.95E-12
BID	1.704426302	6.527653639	8.790886648	2.17E-12
SPP1	4.45474537	7.205695827	8.766574248	2.38E-12
PLAU	1.912233614	6.899629305	8.75280162	2.52E-12
TPX2	1.944996758	7.525574808	8.694016386	3.16E-12
TOP2A	2.182972383	8.355378728	8.662807904	3.57E-12
ARHGEF10L	-1.207885162	7.357862142	-8.657167514	3.65E-12
SPC25	1.764318253	5.504149205	8.650739017	3.75E-12
FSCN1	1.72526339	7.16457589	8.619614427	4.23E-12
FOXM1	1.792870614	6.484733218	8.612614933	4.35E-12
DLGAP5	2.161649362	6.639119474	8.597367462	4.62E-12
PRIM2	1.04710497	4.60800327	8.586799268	4.81E-12
KPNA2	1.320849102	9.964037523	8.581236415	4.92E-12
TRIM13	-1.062555007	7.718443579	-8.578814702	4.96E-12
HPGD	-3.040951021	7.236054163	-8.5632749	5.27E-12
PTDSS1	1.070444299	9.39451385	8.548790734	5.58E-12
PCNA	1.267140499	10.05406125	8.544493791	5.67E-12
KIF18B	1.348295014	7.098571788	8.542809986	5.71E-12
CENPE	1.395878826	6.190682377	8.538481197	5.81E-12
MFAP2	2.219687645	7.48049866	8.519842043	6.25E-12
LRP10	-1.28568362	10.11784868	-8.514518886	6.38E-12
PMM1	-1.358719416	7.293884393	-8.51127865	6.46E-12
DSN1	1.044909795	6.733472702	8.463678289	7.79E-12
CDC6	1.720174381	5.025621197	8.461140814	7.86E-12
MMP1	4.849064064	6.646083476	8.459786342	7.90E-12
CEP55	2.239081941	6.938868582	8.405865453	9.76E-12
MCM6	1.126931379	9.140718591	8.405541789	9.78E-12

SNAI2	1.765878198	8.296516871	8.39344785	1.03E-11
TOPBP1	1.083370823	8.488398316	8.392430176	1.03E-11
KIAA0232	-1.122221964	8.917634723	-8.38913703	1.04E-11
PRC1	1.843624188	8.013505648	8.353519647	1.20E-11
AMOTL2	-1.212124458	8.808587699	-8.352763301	1.20E-11
MMP11	1.647625208	6.425171384	8.328639633	1.32E-11
CDCA3	1.161807784	6.676258368	8.316937679	1.38E-11
FEN1	1.231621687	7.302268307	8.306125556	1.44E-11
FAM63A	-1.301283783	7.62547249	-8.291039115	1.53E-11
HOXC10	1.775549298	5.80418242	8.28548332	1.57E-11
AHNAK	-1.379715876	8.932513285	-8.282695347	1.58E-11
NUDT1	1.083344835	7.476621686	8.25522824	1.76E-11
RNASEH2A	1.219402439	7.9574162	8.244903317	1.84E-11
MMP12	3.499963223	7.083476808	8.227888869	1.96E-11
ADIRF	-2.082099999	9.769580865	-8.21667932	2.05E-11
PBK	2.024753553	7.012561637	8.214300191	2.07E-11
CKS2	1.721199304	9.731536656	8.207643965	2.12E-11
RPL39L	1.733639974	6.005164297	8.205197666	2.15E-11
SUCLG2	-1.229557418	8.592773261	-8.137031484	2.80E-11
FZD2	1.186267112	5.918309916	8.135855461	2.82E-11
LRRFIP2	-1.144402784	7.62790831	-8.115064618	3.06E-11
STIL	1.65515133	6.722003614	8.106056638	3.17E-11
UNC13B	-1.09005733	7.826523004	-8.075888007	3.57E-11
SLC38A6	1.422093172	6.212035236	8.046522125	4.00E-11
GTSE1	1.071905444	6.602064572	8.042613774	4.06E-11
ACAA1	-1.023672726	8.18406946	-8.039490059	4.11E-11
HSPE1	1.227921013	9.690271269	8.034081011	4.20E-11
VRK1	1.10383744	7.646006136	8.027769491	4.31E-11
ACOX3	-1.149448274	6.998862258	-8.021805568	4.41E-11
KIF18A	1.289722525	5.032435496	7.996697153	4.87E-11
DPY19L4	1.040023628	7.423186968	7.970026593	5.41E-11
SECISBP2L	-1.214479002	7.653138253	-7.967207165	5.47E-11
CDK4	1.309109764	9.103198681	7.965764357	5.50E-11
RAD54L	1.226768625	6.707540411	7.964682124	5.52E-11
PALMD	-1.702596623	7.670971071	-7.955977545	5.71E-11
SASH1	-1.936804445	8.961847944	-7.944722051	5.97E-11
CDC45	1.170829253	6.421416085	7.916242872	6.68E-11
DNA2	1.201962125	6.072763622	7.90689208	6.93E-11
GALNT12	-2.150324049	7.712551781	-7.905765228	6.96E-11
KIF15	1.395521255	5.652239539	7.896495776	7.22E-11
BLM	1.542566353	6.490299903	7.890986896	7.38E-11
C1orf112	1.204744893	5.080115784	7.873609485	7.90E-11
HEY1	2.042527537	7.542494614	7.851738986	8.61E-11
AURKB	1.686887785	6.647795914	7.832944645	9.27E-11
FBXO5	1.190654052	6.246732923	7.825223188	9.56E-11
MEST	2.238179245	8.43822802	7.811601591	1.01E-10
CAST	-1.125036301	8.503275822	-7.804347751	1.04E-10
MYBL2	1.098020742	6.844155274	7.781736309	1.13E-10
COL1A1	1.490466677	7.359120994	7.765974784	1.21E-10
SLC20A1	1.297269795	8.488773613	7.743354111	1.32E-10
CHEK1	1.153427512	4.774564659	7.716136187	1.47E-10
CDC25B	1.245855008	8.566710574	7.712664494	1.49E-10
HSPD1	1.302053146	10.75585763	7.711288492	1.50E-10

SPAG16	-1.453127811	7.629466798	-7.684576282	1.66E-10
ILF2	1.113459243	9.282057171	7.677816016	1.71E-10
LMO2	-1.074275927	7.075510364	-7.676001714	1.72E-10
PDLIM2	-1.487907747	7.715431895	-7.673256008	1.74E-10
CCNB2	1.763233697	8.060811346	7.665701597	1.79E-10
CENPN	1.4295236	6.180476131	7.660996552	1.83E-10
HSPB8	-2.300739903	9.460699253	-7.656948614	1.86E-10
GINS1	1.808074328	7.75859993	7.655185299	1.87E-10
THBS2	1.843128844	8.267615452	7.650935292	1.90E-10
RACGAP1	1.088154936	8.736356681	7.649634041	1.91E-10
COBL	-1.904650487	7.431206279	-7.628912631	2.07E-10
RUVBL1	1.204052331	7.281508069	7.627856891	2.08E-10
APMAP	1.097595361	8.640613655	7.62434805	2.11E-10
CXCR2	-3.09042382	6.16922465	-7.616676848	2.17E-10
CENPM	1.085690777	7.312341876	7.610616212	2.23E-10
SMYD3	1.394220945	7.042328876	7.605038938	2.28E-10
KNTC1	1.203348968	6.161969527	7.601954206	2.30E-10
CDC20	1.876099414	8.015036826	7.60112471	2.31E-10
SORBS2	-1.357544009	5.566630148	-7.588959638	2.43E-10
CST1	3.040054962	5.508914244	7.588885464	2.43E-10
RAD54B	1.430818171	5.953347518	7.588726114	2.43E-10
SH3GLB2	-1.306472547	6.346542623	-7.586619829	2.45E-10
SLC39A6	1.177040296	8.533972168	7.566815506	2.65E-10
APOBEC3B	2.796740285	6.643095454	7.561704599	2.70E-10
TK1	1.170138712	7.416015511	7.558432397	2.74E-10
OBFC1	-1.875857349	7.715916229	-7.547602987	2.85E-10
DBF4	1.450831422	7.486114408	7.535304196	3.00E-10
HPRT1	1.011469638	9.479687573	7.505383249	3.37E-10
ENDOU	-3.381356428	6.750113104	-7.482637148	3.69E-10
MPC1	-1.003908351	9.259419593	-7.46842305	3.90E-10
MCM10	1.711378114	5.441406296	7.460008786	4.03E-10
EMP1	-2.379673318	9.588476779	-7.453792898	4.13E-10
NUSAP1	1.504194928	7.460764617	7.421164658	4.70E-10
TLR3	-1.48412877	5.865038882	-7.417190937	4.77E-10
LPIN1	-1.205458214	6.667337161	-7.415784626	4.80E-10
CDT1	1.00795409	5.241439001	7.415252182	4.81E-10
GMNN	1.331875921	8.706101503	7.399911805	5.11E-10
GYS2	-2.490185917	4.541361728	-7.394771407	5.21E-10
GDPD3	-2.125454656	7.410502877	-7.378878523	5.55E-10
BUB1B	1.740660406	7.717363448	7.363072289	5.91E-10
NPEPPS	-1.007798331	8.019316663	-7.349385227	6.23E-10
TAF1A	1.149589892	4.736728702	7.349320173	6.24E-10
CRISP3	-5.319100155	6.697858243	-7.337302013	6.54E-10
SLC24A3	-1.766107749	7.520755958	-7.334914217	6.60E-10
ALDH9A1	-1.198393957	10.59445889	-7.333403624	6.64E-10
MRGBP	1.137936334	6.202927351	7.324408753	6.88E-10
TRIP10	-1.583112323	8.316229437	-7.322322082	6.94E-10
APOC1	1.426431533	7.459102431	7.312569475	7.21E-10
SNAPC1	1.233592803	7.194009223	7.28249906	8.11E-10
SLC16A7	-1.907793926	6.862127274	-7.282381308	8.12E-10
BIRC5	1.295819802	6.370527598	7.271314277	8.48E-10
ABHD5	-1.15921791	6.374223499	-7.258711995	8.91E-10
PLA2G7	1.466316331	4.838821486	7.256548842	8.99E-10

SERPINB6	-1.30324668	8.98389721	-7.255635942	9.02E-10
HLTF	1.243640295	7.832369755	7.24787974	9.30E-10
RPA3	1.003534379	8.871835563	7.24639424	9.35E-10
DUSP5	-2.117306569	8.089179639	-7.238107057	9.67E-10
ITPR2	-1.113966302	5.89049699	-7.22517256	1.02E-09
FAM64A	1.032285619	5.964362461	7.202492358	1.11E-09
NCAPH	1.177006714	6.202251938	7.184185214	1.20E-09
EREG	-2.950221963	6.239615011	-7.177841862	1.23E-09
DTL	1.866176128	7.358776068	7.176932231	1.23E-09
SNX10	2.026359822	6.432124094	7.17275794	1.25E-09
NCAPG	1.451593059	6.567655521	7.143258376	1.40E-09
TMEM246	-1.228897866	6.368872968	-7.133530511	1.46E-09
PDCD6IP	-1.011804759	9.453357312	-7.119943121	1.54E-09
KLF4	-1.729261583	8.862500156	-7.108205179	1.61E-09
FAM189A2	-1.431934368	5.855790503	-7.083010817	1.78E-09
RRAD	-1.747542577	7.032503203	-7.082106204	1.79E-09
DENND4C	-1.067246291	6.876726358	-7.077439434	1.82E-09
VOPP1	1.436502262	9.004261699	7.069354264	1.88E-09
HJURP	1.406186561	6.584383817	7.046279086	2.06E-09
ORC6	1.497752366	8.075470368	7.041944368	2.09E-09
TIPIN	1.189270466	6.124127669	7.022197614	2.26E-09
ARL6IP5	-1.093688794	9.791216014	-7.019445627	2.29E-09
HMMR	1.512215269	6.820746097	7.018514717	2.29E-09
STK39	-1.345899383	8.6631767	-7.014854705	2.33E-09
ECHDC2	-1.426116252	8.851115021	-7.01434054	2.33E-09
HOXD11	1.467349296	5.270022905	7.011740811	2.36E-09
NETO2	1.615062528	7.512736969	7.006801955	2.40E-09
GIPC2	-1.223515504	4.632984047	-7.004178986	2.43E-09
FYCO1	-1.427637734	7.399086349	-6.990668148	2.56E-09
MARCKSL1	1.983540817	8.428222852	6.984037359	2.63E-09
VCAN	2.313177903	7.014512087	6.957429356	2.92E-09
TMEM132A	1.087859682	7.425798287	6.950024839	3.00E-09
TMEM57	-1.034196036	7.1958499	-6.937349187	3.16E-09
CAMK2N1	-1.831368861	8.123066248	-6.937043956	3.16E-09
CYP4B1	-3.108761642	6.632522447	-6.925023008	3.31E-09
GGH	1.535944987	8.521015673	6.91930052	3.39E-09
RAP1A	-1.063053831	10.13572404	-6.88482719	3.88E-09
DDX39A	1.051066014	8.797001855	6.8757744	4.02E-09
SERPINH1	1.643999339	8.164218186	6.866057209	4.18E-09
MAL	-4.259272524	10.0601721	-6.848783831	4.47E-09
EPHX2	-1.432581847	6.489365966	-6.846712307	4.51E-09
CCNG2	-1.796554267	7.946129799	-6.841618485	4.60E-09
SLC25A32	1.086680073	8.284025877	6.826285512	4.88E-09
FNDC4	-1.071432533	7.249163406	-6.818075015	5.04E-09
DHRS1	-1.690165088	8.876733938	-6.811769716	5.17E-09
DUSP1	-1.244223259	8.646419455	-6.808156231	5.24E-09
MELK	1.560613041	8.008960678	6.804580395	5.32E-09
DYNLT3	-1.326776907	10.45175431	-6.789551414	5.64E-09
RPP25	1.118106441	7.054209895	6.787752858	5.68E-09
SHCBP1	1.477515839	6.219281478	6.785468349	5.73E-09
NUP62CL	1.051654111	4.493696915	6.769339375	6.10E-09
SIX1	1.116091996	5.156419974	6.76882066	6.12E-09
HOMER3	1.061629136	6.041256814	6.759971844	6.33E-09

PUS7	1.188553698	8.375475152	6.753520801	6.49E-09
MAFF	-1.135769848	8.708322579	-6.739915325	6.85E-09
ALDH2	-1.00610881	9.62760264	-6.734157568	7.01E-09
SLC16A1	1.201503042	6.474564896	6.726514224	7.22E-09
HLF	-1.914607956	6.619852905	-6.722407713	7.34E-09
LRP12	1.295719177	4.696020895	6.715787136	7.53E-09
GMDS	-1.442927956	7.033975951	-6.715156253	7.55E-09
DDAH1	-1.549108799	8.84264133	-6.711419503	7.66E-09
BORA	1.208892762	6.589906826	6.682890102	8.56E-09
EPS8L1	-1.708197823	7.442757012	-6.67442779	8.85E-09
ACADM	-1.096311212	8.829513286	-6.668315156	9.07E-09
LRRC8D	1.016742884	8.303565495	6.659153532	9.40E-09
NELL2	1.936810082	7.127199406	6.641776907	1.01E-08
POSTN	2.121777643	6.795588431	6.639625934	1.01E-08
EPCAM	2.35762699	9.427710184	6.597588973	1.20E-08
MRPS17	1.083487331	8.286632128	6.579712666	1.28E-08
LMNB2	1.099589133	7.580063889	6.577585252	1.29E-08
AKR1B1	1.265144091	8.819990816	6.573892648	1.31E-08
MMP13	2.369110229	4.983430045	6.567058506	1.35E-08
LPCAT1	1.490876217	7.19315445	6.565106281	1.36E-08
ASPM	1.858555815	7.447521753	6.557332115	1.40E-08
PFN2	1.716179483	9.863243473	6.519976297	1.62E-08
SOX12	1.005844496	5.321969952	6.515688318	1.65E-08
ECM1	-2.678704773	10.05000491	-6.500794023	1.75E-08
UBE2S	1.312039953	8.76249792	6.48974512	1.82E-08
CH25H	-2.141814108	7.076605352	-6.488497323	1.83E-08
SLC39A4	1.014237371	8.054813603	6.483742083	1.87E-08
POLE2	1.34502784	6.603874342	6.475964493	1.92E-08
RFC3	1.035954759	5.763774358	6.475919714	1.92E-08
ADH1B	-2.453041127	5.68279517	-6.469017087	1.98E-08
OIP5	1.497812871	5.899690887	6.455633931	2.08E-08
ASCC2	-1.055701067	7.732088857	-6.427350758	2.32E-08
GULP1	-1.017379726	6.565007124	-6.425325007	2.34E-08
CWH43	-2.482389049	6.027968431	-6.423266692	2.36E-08
RANBP9	-1.457872302	7.948557272	-6.419594458	2.40E-08
MINPP1	1.119203576	6.090160258	6.419038304	2.40E-08
NAGK	-1.065509547	9.540799258	-6.418504353	2.41E-08
SCNN1B	-2.109076334	6.815070242	-6.401219874	2.57E-08
ERVMER34-1	1.244827375	5.013328211	6.394842364	2.64E-08
PLAGL1	-1.200458531	8.043449973	-6.389577085	2.69E-08
KCNS3	1.522618384	7.318350448	6.387978319	2.71E-08
RRAGD	-1.569773246	7.71801777	-6.376957011	2.83E-08
CDH11	1.746343873	6.524205167	6.363448643	2.98E-08
EHD3	-1.68631343	7.595078768	-6.355268791	3.08E-08
CCNA2	1.255447484	6.102411716	6.354421594	3.09E-08
ITGA6	1.461204957	8.055560473	6.342889133	3.23E-08
CPPED1	-1.128305097	5.342588077	-6.339104103	3.28E-08
CHST1	1.273792191	6.092348363	6.332424293	3.36E-08
PIM1	-1.029482137	8.30190973	-6.327732579	3.43E-08
CCNB1	1.606319688	8.085052349	6.325302675	3.46E-08
CRYAB	-1.802493396	9.558887745	-6.310812537	3.66E-08
RORA	-1.718678586	6.772067576	-6.306063983	3.73E-08
SULF1	1.85623994	7.313133721	6.303337457	3.77E-08

TP53I3	-1.689527898	8.332435219	-6.300446768	3.81E-08
FST	1.656128456	6.850757783	6.284770081	4.05E-08
BLVRB	-1.066920718	9.615295128	-6.277938709	4.16E-08
FLG	-3.318918192	6.201555285	-6.265032692	4.37E-08
VPS37B	-1.113807614	7.897460047	-6.262963909	4.41E-08
BEX4	-2.182850805	7.844355017	-6.257344437	4.50E-08
MXD1	-1.919636498	6.045719668	-6.257057844	4.51E-08
PTN	-1.518334494	7.49804352	-6.254678484	4.55E-08
NFE2L3	1.492209556	5.599692297	6.254487339	4.55E-08
P2RY14	-1.730048355	5.845486517	-6.242643608	4.77E-08
XK	-1.572154353	5.762016124	-6.239548936	4.82E-08
C2orf54	-2.033350697	6.332541111	-6.220919557	5.19E-08
RAI14	1.465023743	7.923295964	6.220467218	5.19E-08
LEPREL4	1.242356657	6.424179523	6.216267481	5.28E-08
DPYD	-1.598841907	7.817300923	-6.212906467	5.35E-08
COL3A1	2.109960189	10.76570006	6.200879548	5.60E-08
DFNA5	1.388185796	7.731729665	6.199359835	5.64E-08
CBS	1.250652618	5.098307541	6.196385549	5.70E-08
LAMC2	1.826649074	5.882673053	6.189772034	5.85E-08
FAP	1.61721806	6.067835605	6.187209477	5.91E-08
TFAP2B	-1.36705308	4.761040291	-6.183696923	5.99E-08
GEMIN2	1.049983711	6.145446527	6.180180651	6.07E-08
MEIS1	-1.255261868	8.335748559	-6.17110004	6.29E-08
MKI67	1.078856342	6.230212788	6.169540677	6.33E-08
COL5A2	2.118963542	8.186121645	6.158735177	6.60E-08
COL1A2	2.396906845	10.95578733	6.156206196	6.66E-08
SLURP1	-3.476268348	8.718251885	-6.152015615	6.77E-08
ZNF365	-1.701405295	6.935039626	-6.143229809	7.01E-08
PSCA	-2.639879247	7.286379123	-6.142877074	7.02E-08
ZFP36	-1.368728156	10.61102456	-6.142768642	7.02E-08
PARP12	1.003000453	8.165330419	6.140650482	7.08E-08
ZIC1	2.410119215	5.308149781	6.135981989	7.20E-08
ATP1B3	1.229453291	11.43702103	6.131510928	7.33E-08
TTC9	-2.013763409	6.661084779	-6.122468502	7.59E-08
GPR126	-1.515289802	6.195566199	-6.117508199	7.74E-08
TGFBI	2.029998762	9.167479728	6.103363778	8.17E-08
JAG2	1.004834757	7.563630335	6.100620658	8.26E-08
TJP1	-1.234707819	8.354581708	-6.098555212	8.33E-08
IGF2BP2	1.703120091	8.781146943	6.082673154	8.85E-08
SERPINE1	1.755316754	6.934077303	6.0816666	8.89E-08
FAT1	1.104537253	9.16839448	6.058411383	9.72E-08
ANXA9	-1.842897061	5.609394792	-6.052845091	9.93E-08
COL7A1	1.287634421	7.436208351	6.051916735	9.97E-08
TMEM97	1.090697511	7.634116831	6.040038488	1.04E-07
ITM2A	-1.760589248	7.551787553	-6.034391479	1.07E-07
CDC7	1.131160729	6.827712706	6.033364509	1.07E-07
PRSS3	-2.109720559	7.440895375	-6.007025735	1.19E-07
GNE	-1.111201836	6.932360551	-6.000247222	1.22E-07
LAMB3	1.937185205	8.571078608	5.985954167	1.29E-07
ACOX2	-1.080173104	5.686766134	-5.962709468	1.41E-07
PPAP2C	1.13307142	7.115812833	5.956357165	1.44E-07
BBOX1	-2.436650973	5.585401853	-5.948272099	1.49E-07
ALS2CL	-1.184881257	7.307334495	-5.942168954	1.52E-07

MANSC1	-1.301252713	9.006095897	-5.929406175	1.60E-07
EFNA1	1.028794205	8.518005054	5.924429417	1.63E-07
ABCC5	1.18346226	8.764831281	5.917913457	1.67E-07
PPFIBP2	-1.180663268	7.408984018	-5.912882707	1.70E-07
TMPRSS2	-1.284629527	5.902724929	-5.905517967	1.75E-07
MMP10	2.723596663	5.415518496	5.903320355	1.77E-07
NUDT11	1.165080211	6.575003462	5.901201439	1.78E-07
MFHAS1	1.331933451	7.498537919	5.886656794	1.88E-07
RRM2	1.738216808	9.161244783	5.865355463	2.04E-07
P4HA1	1.122530667	7.686551349	5.85170781	2.15E-07
LRP8	1.242336114	6.452753383	5.842388171	2.23E-07
PRSS2	-1.325682804	6.093087979	-5.841108594	2.24E-07
CLIC3	-3.425161933	8.30032242	-5.841060373	2.24E-07
BLNK	-2.234559671	8.097304262	-5.827986426	2.36E-07
MMP3	2.329265139	5.239332391	5.808757536	2.54E-07
PPFIA1	1.732272635	7.243551699	5.804991886	2.57E-07
FAM107A	-1.243736323	6.266950286	-5.781922374	2.81E-07
CYP2E1	-1.397127985	5.491956848	-5.759383099	3.06E-07
RNF141	-1.260816953	7.559295993	-5.750446645	3.17E-07
DSG2	1.548878677	9.268707661	5.747603479	3.20E-07
CST4	1.118695867	7.069119226	5.732996234	3.39E-07
ARPC1B	1.267598276	9.399256235	5.732445205	3.39E-07
ARTN	1.026512199	5.817541471	5.725494709	3.49E-07
PDZD2	-1.696401097	7.359817568	-5.718121247	3.58E-07
EPS8L2	-1.517222819	8.504431466	-5.717573137	3.59E-07
DEPDC1	1.03470924	5.199286921	5.706588065	3.75E-07
OR7E14P	-1.594578797	5.408648261	-5.704881808	3.77E-07
UPK1A	-2.210858786	5.892515127	-5.691353634	3.97E-07
SLC16A6	-1.698422759	6.343197948	-5.684235306	4.08E-07
CEACAM1	-1.577385166	6.543698163	-5.682047133	4.11E-07
TXNRD1	1.471616101	8.929886818	5.673737047	4.24E-07
SNX24	-1.1170652	6.849058399	-5.665630697	4.38E-07
INHBA	1.297396814	5.312730603	5.658588205	4.50E-07
TGM3	-3.832234314	9.340107805	-5.658506216	4.50E-07
GCLM	1.640238425	7.663520133	5.648142492	4.68E-07
ZWINT	1.368481313	8.490245379	5.643191642	4.77E-07
CXCL1	1.85328565	6.765082255	5.626089986	5.09E-07
ISG15	1.971363282	8.788131488	5.625228253	5.10E-07
LAMP3	1.822988556	7.757428913	5.621496995	5.18E-07
MOXD1	-1.103614824	7.040060317	-5.599773187	5.62E-07
KLHL7	1.183895939	5.247254783	5.592821625	5.77E-07
TM7SF2	-1.165973915	7.007316616	-5.579272725	6.07E-07
GOLM1	1.408377586	6.63321357	5.565273642	6.40E-07
HMGA2	1.887224938	5.460375055	5.564908024	6.41E-07
FUT6	-1.511777963	6.171366984	-5.55907773	6.56E-07
CFD	-2.118084767	8.608763664	-5.557420931	6.60E-07
CRNN	-4.334729425	9.506117304	-5.555102613	6.66E-07
SLC6A1	-1.018700361	4.416132707	-5.535292925	7.17E-07
SERPINB1	-2.160123499	10.11823751	-5.535162278	7.18E-07
CEACAM7	-1.598312389	5.236806093	-5.51934539	7.62E-07
CRCT1	-3.857159234	8.206883515	-5.515098212	7.74E-07
FMO2	-2.674284873	7.637144964	-5.514159006	7.77E-07
NBEAL2	-1.014894877	7.391033632	-5.49696666	8.29E-07

FAM60A	1.178714946	9.149403267	5.48448663	8.69E-07
RBPMS	-1.247614691	6.789221182	-5.484433602	8.69E-07
ETHE1	-1.043853471	8.722818257	-5.476008037	8.97E-07
IL36A	-1.396288164	5.834141738	-5.46918669	9.20E-07
PTPLAD1	1.126313873	6.658483648	5.463357944	9.41E-07
ADRB2	-1.117164135	6.197870456	-5.461474519	9.47E-07
CNN3	-1.514995125	8.398464822	-5.459272875	9.55E-07
MYO1B	1.022382564	6.850420017	5.444660754	1.01E-06
IL13RA1	-1.164694612	7.223973604	-5.434262628	1.05E-06
LMNB1	1.211875436	7.460129313	5.433029895	1.05E-06
SLC12A8	1.232441587	6.189041274	5.426414483	1.08E-06
NDC1	1.007525093	5.432608475	5.416551967	1.12E-06
MAOA	-1.157324028	7.887765553	-5.407538685	1.16E-06
ABAT	-1.117779677	6.140904902	-5.403788598	1.18E-06
GCHFR	-1.285055738	6.940895182	-5.393832273	1.22E-06
CDH3	1.953060531	8.460940503	5.385459614	1.26E-06
ABCA8	-2.471966414	6.048605663	-5.37898847	1.29E-06
NUAK1	1.347938099	7.485184177	5.375120512	1.31E-06
FUT3	-1.58540827	6.424774916	-5.368951241	1.34E-06
KLK13	-1.861037569	6.720663989	-5.362345784	1.37E-06
SLC6A8	1.164911893	8.661959638	5.344858324	1.47E-06
KIAA0101	1.198433089	8.328489219	5.343976221	1.47E-06
TIPARP	-1.021900102	9.420060621	-5.335433541	1.52E-06
NMRK1	-1.115994131	8.746779781	-5.332563599	1.54E-06
FJX1	1.06369423	6.843562701	5.327728957	1.56E-06
TGFBR3	-1.65987085	7.524036117	-5.324844947	1.58E-06
PRAME	1.318076634	5.286174496	5.321606449	1.60E-06
KDELC1	1.020068827	5.387228711	5.309274301	1.68E-06
FOS	-1.916547259	9.120589671	-5.301201915	1.73E-06
PPAT	1.058892091	6.500764304	5.29857556	1.74E-06
YOD1	-1.377260056	4.41416746	-5.295793335	1.76E-06
CYP2J2	-1.466179453	6.604443669	-5.293628267	1.78E-06
LIMCH1	-1.399585431	6.607329676	-5.276585577	1.89E-06
CXCL8	1.868328377	6.255563707	5.273494921	1.92E-06
ANXA3	-1.696220792	8.217049887	-5.268632589	1.95E-06
HOXD10	1.144169477	4.618552555	5.260094029	2.01E-06
KIF11	1.328604789	6.992155241	5.238787712	2.18E-06
TBL1XR1	1.194352507	6.542072609	5.234393976	2.22E-06
TDO2	1.373238098	5.076280729	5.217694346	2.36E-06
GALNT6	1.298541305	6.062377405	5.216697531	2.37E-06
CYP2C18	-2.014419843	6.785492332	-5.208836201	2.44E-06
SEL1L3	1.213377931	6.773729926	5.202330114	2.50E-06
COL6A3	1.658104602	10.33562993	5.168673667	2.83E-06
RUNX3	1.045712717	6.296075947	5.164019934	2.88E-06
ELOVL4	-1.455415084	6.199222953	-5.160805092	2.91E-06
CES2	-1.425237003	9.169676894	-5.159760648	2.92E-06
IGF2BP3	1.584708123	6.005815803	5.152568193	3.00E-06
ALCAM	1.337210276	8.491170918	5.150128881	3.03E-06
ANO1	2.546960241	7.302857586	5.147644321	3.06E-06
GPNMB	1.459214307	10.81117457	5.146057188	3.07E-06
SYNPO2L	-1.792635933	4.321039847	-5.130408591	3.26E-06
MAOB	-1.679314149	7.404996853	-5.123313175	3.34E-06
TNFAIP6	1.607139287	6.600896179	5.119472432	3.39E-06

CXCL10	1.727803909	6.723350043	5.105465878	3.57E-06
BGN	1.194044927	6.694907346	5.0963648	3.69E-06
SULT2B1	-1.915321133	7.657483317	-5.095091377	3.71E-06
SOAT1	1.245602294	5.238108742	5.093070296	3.74E-06
C1orf116	-1.938749938	7.716777713	-5.079264022	3.93E-06
TMOD3	-1.670564255	5.961169013	-5.07479726	4.00E-06
PPL	-2.148804819	10.22861441	-5.065416151	4.14E-06
DNASE1L3	-1.670324393	6.627677734	-5.064436404	4.15E-06
ZDHHC13	-1.268483181	8.003373285	-5.044481724	4.47E-06
CD207	-1.225759129	5.519236176	-5.038805374	4.56E-06
PLOD3	1.036387741	7.732173915	5.038506361	4.57E-06
SPRY1	-1.102631704	7.612014145	-5.023507676	4.83E-06
MMP9	1.623301988	6.626378221	5.018990677	4.91E-06
NCF2	1.185743372	5.91655356	5.00613468	5.14E-06
PAK1	1.059063584	4.90710763	5.004948498	5.17E-06
EN1	1.30149296	4.063533105	5.003830517	5.19E-06
HERC5	1.219026974	6.374257807	4.991316044	5.43E-06
TTK	1.714306174	7.338147103	4.991013689	5.44E-06
ZNF426	-1.533013605	7.225288079	-4.985968859	5.54E-06
ITGA8	-1.701576673	6.152023576	-4.980712738	5.64E-06
SNX16	-1.237622152	5.920913779	-4.976251185	5.74E-06
EZH2	1.370283374	7.092897075	4.934403898	6.68E-06
TGM1	-2.358576178	9.354419241	-4.932502508	6.73E-06
DEPTOR	-1.426645753	6.991021627	-4.930802501	6.77E-06
COL5A1	1.51428014	7.325007022	4.918822117	7.07E-06
UCHL1	2.239654431	7.207359543	4.913829698	7.20E-06
UPK3B	-1.466888857	6.198876945	-4.909211961	7.33E-06
CYP3A5	-1.842872939	6.577555779	-4.908372376	7.35E-06
FADD	1.465426344	8.28094624	4.90733583	7.38E-06
PDE2A	-1.159050917	6.480085149	-4.898731278	7.61E-06
TJP3	-1.042643275	6.219556705	-4.894826314	7.72E-06
SLC39A14	1.507533481	7.949138939	4.892120704	7.80E-06
SCEL	-3.441435803	8.377481511	-4.880478901	8.13E-06
MALL	-2.057866286	9.708695437	-4.86349407	8.65E-06
PANX1	1.077699406	6.284656107	4.860673624	8.74E-06
IL1RN	-1.752643921	7.348822315	-4.847721517	9.16E-06
KLF2	-1.20075278	8.204251986	-4.842651221	9.33E-06
ODC1	1.694190395	10.07041865	4.821406279	1.01E-05
LUM	1.566447661	10.41513257	4.818558651	1.02E-05
MUC1	-1.235041827	7.822875526	-4.792318416	1.12E-05
SLC2A1	1.294749113	7.116169587	4.792023556	1.12E-05
TCN1	-1.928930252	6.525837239	-4.784414459	1.15E-05
POPDC3	1.508764966	4.98036158	4.781265714	1.16E-05
PTTG1	1.053131126	9.566834063	4.777842735	1.18E-05
GPR110	-1.007796278	4.946679277	-4.772796244	1.20E-05
VLDLR	-1.098283826	7.476813152	-4.769651266	1.21E-05
ACPP	-2.232300059	7.467262307	-4.764436113	1.24E-05
PTHLH	2.194587759	6.060038088	4.758995052	1.26E-05
KLK12	-2.087031521	7.697869423	-4.743827111	1.33E-05
S100P	-2.371874235	8.912175664	-4.731973904	1.39E-05
OLFML2B	1.145851381	6.619270828	4.726360619	1.42E-05
TMPRSS11E	-3.069847619	8.260938279	-4.720224071	1.45E-05
NOX4	1.073240987	4.441355684	4.717231998	1.47E-05

CCDC6	-1.052514445	7.623753199	-4.716134022	1.47E-05
PLAUR	1.065452618	6.815236903	4.712947433	1.49E-05
EVPL	-1.678811028	8.135978907	-4.708100136	1.51E-05
LAMA3	1.283018942	7.87119489	4.70566131	1.53E-05
TMEM100	-1.609331594	4.886646867	-4.699229311	1.56E-05
MLF1	1.250998788	6.764065113	4.699076382	1.56E-05
PLAC8	-2.284478753	8.158658739	-4.631561916	1.99E-05
CTSC	1.066204713	9.198402916	4.629279537	2.01E-05
NR3C2	-1.179332823	5.114380832	-4.625546693	2.03E-05
SPINK5	-3.107861524	10.17397517	-4.608242297	2.16E-05
LY96	1.460771206	6.881264798	4.604167515	2.19E-05
EPHX3	-2.098806767	6.923962458	-4.60004669	2.23E-05
PITX2	1.298294823	5.265168048	4.581780726	2.37E-05
MAGEA11	1.954496103	4.266704956	4.574156002	2.44E-05
IL18	-2.229643293	7.517104643	-4.573897343	2.44E-05
CRIP1	-1.223935978	9.14996372	-4.573729959	2.44E-05
TPPP3	-1.375184309	7.587609519	-4.561135068	2.55E-05
IGFBP3	1.652140773	8.320339605	4.560675431	2.56E-05
ALOX15B	-1.174733536	6.56296768	-4.550068965	2.66E-05
FABP6	1.131413706	6.022196208	4.546873969	2.69E-05
ELOVL6	-1.275449586	7.010586021	-4.544752724	2.71E-05
TMEM158	1.330817635	7.233378752	4.541791286	2.73E-05
CLCA4	-3.535008301	8.785177769	-4.539629835	2.76E-05
BAMBI	1.465336694	5.968711581	4.523815844	2.91E-05
THY1	1.062841355	7.606407826	4.506669145	3.09E-05
HOPX	-2.012868073	10.68565111	-4.491387283	3.27E-05
PRSS3P2	-1.093240166	7.127472354	-4.468439027	3.54E-05
IFI44	1.067782416	6.150986151	4.462724041	3.61E-05
MICB	1.031057376	5.830488802	4.453389738	3.73E-05
PER3	-1.017015217	6.74002553	-4.451710411	3.75E-05
CCNA1	1.617924536	4.950398943	4.442557919	3.88E-05
RBP1	1.414786409	8.478814116	4.437350217	3.95E-05
PTH2R	1.462620185	4.294608902	4.434410424	3.99E-05
ACKR1	-1.669497413	6.875837897	-4.433656922	4.00E-05
HSPA2	-1.075628705	9.053855456	-4.420096583	4.19E-05
CDA	-1.778123465	7.056100225	-4.410575327	4.33E-05
ACKR3	1.494352652	8.715287683	4.400931493	4.48E-05
GREM2	-1.066997182	4.60321965	-4.398829586	4.51E-05
PTK6	-1.964197941	8.308245601	-4.389524264	4.66E-05
DENND2D	-1.018757312	7.747063044	-4.382217873	4.78E-05
SMAGP	-1.338990876	8.363127389	-4.376763232	4.87E-05
SIM2	-1.119421136	6.05297613	-4.374478837	4.91E-05
MMD	1.124081745	7.592914449	4.371038451	4.97E-05
BSPRY	-1.198927357	6.95550078	-4.361578701	5.14E-05
POF1B	-1.198529914	6.274042962	-4.361147059	5.15E-05
PLP1	-1.633243144	5.222213787	-4.357346929	5.21E-05
ADORA2B	1.261490247	7.028126159	4.353938905	5.28E-05
RBM47	-1.223727957	9.112490031	-4.351526532	5.32E-05
KLK11	-1.815424935	9.141318518	-4.349785449	5.35E-05
RERGL	-1.247813925	4.277205566	-4.348265796	5.38E-05
CEMIP	1.070225257	5.747422496	4.343763188	5.47E-05
PMEPA1	1.420467509	6.340868815	4.339775376	5.54E-05
CXCL12	-1.454326397	6.790920945	-4.329526269	5.74E-05

COL4A1	1.424241876	8.2136361	4.324497828	5.84E-05
PAX9	-1.164496697	6.775778196	-4.312813025	6.08E-05
CCDC69	-1.638500823	6.913993764	-4.298611128	6.39E-05
IL33	-1.607026767	5.518700452	-4.294526139	6.48E-05
CEACAM5	-1.244034813	7.51577011	-4.272889656	6.98E-05
GATM	-1.050126462	7.743137696	-4.27115022	7.02E-05
ERO1L	-1.640739081	7.834167407	-4.267622958	7.11E-05
ZBTB16	-1.418067739	4.986703892	-4.264007772	7.20E-05
NTS	3.254739389	7.046335208	4.263235601	7.21E-05
ALOX12	-2.046435989	7.380923088	-4.261893106	7.25E-05
SLC27A6	-1.167438344	4.734351607	-4.259837717	7.30E-05
DSG1	-2.152441404	6.429670427	-4.242382244	7.75E-05
GYPC	-1.030555245	7.560338932	-4.213425214	8.56E-05
TWIST1	1.047223408	6.341716032	4.208135957	8.71E-05
SELENBP1	-1.170495429	6.606893333	-4.180554369	9.57E-05
GPM6B	-1.17687746	6.737258844	-4.169208668	9.95E-05
C7	-2.09881265	6.210808786	-4.161210324	0.000102225
SLPI	-1.696687891	10.99322861	-4.156346149	0.000103927
PKP3	-1.151851048	7.863028782	-4.140046372	0.000109834
DHCR7	1.108555928	7.57581754	4.136161328	0.000111289
PROS1	-1.107670632	7.830761831	-4.130644862	0.000113387
LAMB1	1.00699378	7.755303381	4.127501918	0.0001146
MYOC	-1.423990889	6.389274046	-4.112746522	0.000120462
COCH	1.127765248	5.066429877	4.105233154	0.000123557
DHRS9	-1.338831879	7.517183991	-4.102902938	0.000124532
SPRR2C	-2.250084443	7.382373771	-4.097571332	0.000126792
ATP1A2	-1.469343614	5.34813372	-4.096858302	0.000127097
PGBD5	1.185304932	5.887174684	4.081205352	0.000133979
MAGEA6	2.630360521	5.927162436	4.076401224	0.000136163
WFDC1	-1.231369956	7.109572397	-4.0574372	0.000145121
CHST2	1.022282729	7.010731043	4.035850864	0.000156012
KRT4	-2.002507921	8.482290658	-4.025801223	0.000161347
PPP1R1A	-1.092573821	4.991570261	-4.005387127	0.000172731
GIMAP6	-1.062665676	6.132670405	-4.005221342	0.000172826
GLTP	-1.282625129	9.239966779	-3.989851985	0.000181909
PRSS23	1.155872431	7.676964454	3.977919017	0.000189276
SPARC	1.353060847	9.779613823	3.975014216	0.000191112
KRT24	-2.13365177	6.388854638	-3.967679226	0.000195826
FXYD1	-1.341744912	6.144942228	-3.963521524	0.000198547
CTSK	1.264417285	8.075236641	3.963486547	0.00019857
SFRP4	1.600813313	5.907119874	3.956752669	0.000203056
LY6G6C	-1.024166039	7.773495761	-3.903718811	0.000241955
SPAG1	-1.030100386	6.66535198	-3.901850184	0.000243449
ITIH5	-1.272457271	5.725253764	-3.882295859	0.00025962
SCN9A	1.006865162	4.180246277	3.865623562	0.000274219
CLDN1	1.206026658	6.436575215	3.861033982	0.000278374
MMP7	1.158277657	6.699357801	3.859828098	0.000279476
TMEM45A	1.2722226	8.383700254	3.858753034	0.000280462
RSAD2	1.086501952	5.433447977	3.853559015	0.000285272
SERPINB2	-2.483366067	8.567758091	-3.833094824	0.000305004
RGS5	-1.15745082	8.469398545	-3.824116048	0.000314069
CYP24A1	1.032404957	4.799602351	3.823705981	0.000314489
FHL1	-1.369191576	6.890861684	-3.822929755	0.000315286

ADAMDEC1	1.273484717	5.464867993	3.814420408	0.000324148
PLBD1	-1.023417681	9.234629024	-3.799472218	0.000340298
ZNF185	-1.84741002	9.321269922	-3.780042169	0.000362445
SLC7A11	1.447724707	5.827471922	3.766667687	0.000378486
AIM1	-1.438117831	9.558128938	-3.766355473	0.000378868
BASP1	1.038963983	9.220795458	3.755667884	0.000392186
IVL	-1.717791712	8.833750121	-3.750264442	0.000399088
MMP2	1.420819052	8.851450097	3.748537667	0.000401319
ZWILCH	1.132263549	6.387325405	3.747257632	0.00040298
PDZRN3	-1.242249076	7.81046893	-3.742647249	0.000409017
CRABP2	-1.749481389	9.861307453	-3.7408427	0.000411403
ENO2	1.145153156	6.100812754	3.740574666	0.000411759
KHDC1L	1.153122373	4.438997783	3.740340346	0.00041207
SLCO1B3	1.398956422	4.768919178	3.737614834	0.000415705
SEPP1	-1.098256529	10.65157379	-3.733493814	0.00042126
GCNT3	-1.068704449	6.00003579	-3.729019189	0.000427373
EYA2	-1.163424937	6.642810064	-3.72398165	0.000434355
KRT8	1.13021151	8.762467269	3.708749098	0.000456139
PRSS21	1.089633523	5.513825515	3.706364706	0.000459642
SAMD9	-1.318180493	7.69579519	-3.705760987	0.000460534
FOSB	-1.510883586	7.77567357	-3.671912838	0.00051322
HBB	-1.576920574	8.122785537	-3.646601146	0.000556332
ENAH	1.181756675	8.4413312	3.630783934	0.000585005
CTTN	1.028333576	7.954384196	3.618641462	0.000607968
COX7A1	-1.49578902	7.530549478	-3.601042785	0.00064278
GABRP	-1.948538074	6.234039637	-3.595171759	0.00065481
SERPINB13	-1.383334046	6.335424826	-3.592297669	0.000660777
RAB11FIP1	-1.365794252	8.893797121	-3.582795684	0.000680876
PID1	-1.177039201	5.456729126	-3.562366576	0.000726079
TSPAN8	-1.239237836	4.65613534	-3.555566226	0.000741749
SPOCK1	1.122045394	6.714862236	3.553288066	0.000747071
CXCL6	1.189228409	4.274094042	3.545020623	0.000766689
AQP3	-1.460419424	9.524240867	-3.538473898	0.00078257
EXPH5	-1.076768654	6.567679189	-3.534258121	0.000792962
NID2	1.196106934	6.453910886	3.531721894	0.000799277
IFI6	1.419663753	8.635600864	3.528445144	0.000807507
CXCL11	1.076672906	4.179671679	3.523819054	0.000819263
TMPRSS11D	-1.76050237	8.185798216	-3.509665572	0.000856249
RCAN2	-1.623062256	6.934787228	-3.509491884	0.000856713
PXDN	1.329656667	7.331187266	3.508747143	0.000858703
HELLS	1.007585433	6.472604434	3.48684183	0.000919255
HPSE	-1.349425046	7.085069086	-3.478299953	0.000943948
CALB1	1.850914307	4.709161186	3.462500127	0.000991291
ANXA1	-1.000909951	12.79840499	-3.447175475	0.001039357
ASPN	1.427223569	7.736474538	3.441446822	0.001057886
CD24	-1.458211048	10.8043091	-3.440674442	0.001060408
RNASE4	-1.225884955	6.14754727	-3.422215253	0.001122407
PITX1	-1.524607412	8.679760732	-3.421173044	0.001126008
VCAM1	1.081750869	7.306630994	3.417268321	0.001139596
CASQ2	-1.335291963	6.645665184	-3.415785042	0.001144799
VAV3	-1.427682081	7.733291878	-3.415495167	0.001145818
DPT	-1.326960232	5.841355401	-3.397651338	0.001210249
CEACAM6	-2.067073326	9.528303564	-3.396487034	0.00121457

IFI44L	1.348009376	6.458436329	3.394783182	0.00122092
MLLT11	1.071647555	7.947486765	3.385041391	0.001257833
AQP1	-1.173841604	7.425721823	-3.3720916	0.001308539
C3orf14	-1.100971441	7.263327961	-3.365374245	0.001335597
TIMP1	1.132352029	9.821106701	3.361198059	0.001352684
DUOX1	-1.140922905	7.150966183	-3.342574417	0.001431434
OGN	-1.345855187	4.574174776	-3.326641837	0.001502223
MT1M	-1.529292703	5.699970733	-3.324082111	0.001513899
COL4A2	1.161535749	8.435127804	3.32252123	0.001521062
PCP4	-1.664512503	7.158908649	-3.315328923	0.001554479
MPZL2	-1.327493549	7.711391618	-3.313022389	0.001565342
CHRDL1	-1.752435313	6.999070165	-3.300451194	0.001625817
CDH19	-1.354332868	4.976022482	-3.280066609	0.001728585
IL36RN	-1.122158205	6.372678944	-3.27916294	0.00173328
EHF	-1.857474206	6.577911828	-3.26998637	0.001781638
RHCG	-2.279873901	10.43062812	-3.259821126	0.001836689
MAGEA12	1.212600908	4.737532409	3.257853209	0.00184753
CCL20	1.405976308	6.609593005	3.239045382	0.001954223
AMIGO2	1.046681393	7.675064184	3.235202904	0.001976722
EGFL6	1.039877169	8.086389837	3.166092697	0.002425398
ZNF91	-1.247371289	8.251702397	-3.151595303	0.002530919
LCN2	-1.672346759	9.4777671	-3.106780262	0.00288496
NACC2	-1.000828104	7.327718182	-3.099039642	0.002950619
MAGEA1	1.066371302	5.084196296	3.095439196	0.002981633
AOC3	-1.337243668	7.520836513	-3.087310213	0.003052781
CST6	-1.312094977	7.417402884	-3.074571219	0.003167476
AKR1C1	1.297879593	10.06479694	3.049082772	0.003409143
KRT13	-2.310328876	11.48046041	-3.017071371	0.003737036
LMOD1	-1.13968492	6.443549208	-2.926676892	0.004828322
LGALS1	1.193082986	10.80002107	2.909370948	0.005068368
PLN	-1.521493978	5.791038956	-2.90821703	0.00508476
AZGP1	-1.045676445	4.212655313	-2.904552621	0.005137143
MYH11	-1.367957224	8.554116678	-2.8903634	0.005344728
TMEM35	-1.082066856	5.544201184	-2.868320925	0.005682652
CXCL9	1.059880811	6.403919966	2.855961214	0.00588067
S100B	-1.003659156	4.826488471	-2.839339076	0.006157036
GPX2	1.154008156	9.769684474	2.830073566	0.006316243
SPRR3	-1.944323217	11.97742024	-2.787733417	0.007093226
CNN1	-1.468896124	8.214522712	-2.77173001	0.00740914
PDZK1IP1	-1.371587949	8.034430296	-2.71639849	0.008603694
FERMT1	1.003092564	8.684388797	2.63962465	0.010554236
S100A12	-1.457778002	7.622004171	-2.552499677	0.013250299
KRT14	1.590629347	11.61719857	2.524760578	0.014231471
NMU	-1.217787729	8.825563812	-2.515985465	0.014555271
SRPX	-1.031508502	7.167159415	-2.511589744	0.014719962
KLK7	-1.101581182	7.549946311	-2.469257865	0.016394253
PRKAR2B	-1.159340431	6.721293198	-2.469075777	0.016401812
HSPB7	-1.020374898	5.971994226	-2.45705085	0.016907942
ACTG2	-1.382766162	9.689750285	-2.447723794	0.017310147
APOD	-1.033500284	7.023699603	-2.431455119	0.018032318
BCHE	-1.207619776	6.555984638	-2.418858381	0.018609948

adj.P.Val	B
1.52E-16	36.42393634
1.02E-13	29.47445612
2.48E-13	28.22389616
6.62E-13	26.82839987
6.62E-13	26.7742775
8.63E-13	26.33967189
2.24E-12	25.26198377
2.43E-12	25.05232484
3.44E-12	24.60172505
3.65E-12	24.44210979
4.24E-12	24.14798099
4.24E-12	24.11922338
4.80E-12	23.87569937
4.80E-12	23.84769589
5.23E-12	23.6968321
6.70E-12	23.34978933
6.70E-12	23.33374628
9.58E-12	22.91612259
1.02E-11	22.72156411
1.14E-11	22.52981359
1.14E-11	22.52081263
1.21E-11	22.42540753
1.90E-11	21.90457369
1.92E-11	21.85806514
2.25E-11	21.63146696
2.35E-11	21.55743638
2.36E-11	21.52109711
2.40E-11	21.47327854
2.47E-11	21.41725833
2.63E-11	21.31062956
2.63E-11	21.29775247
2.96E-11	21.13100055
2.96E-11	21.1146707
2.96E-11	21.10323257
3.83E-11	20.80180474
4.24E-11	20.64757139
4.24E-11	20.63834155
4.24E-11	20.63058096
5.10E-11	20.42374298
5.10E-11	20.40626564
5.44E-11	20.31475188
5.44E-11	20.30181397
5.62E-11	20.24965598
5.69E-11	20.21717421
5.70E-11	20.19496427
6.94E-11	19.9617871
6.94E-11	19.92591321
6.94E-11	19.91093543
8.28E-11	19.70426016

8.28E-11	19.69518546
9.01E-11	19.58711969
9.29E-11	19.54132445
1.02E-10	19.40958027
1.24E-10	19.19753779
1.26E-10	19.15474002
1.26E-10	19.15162992
1.30E-10	19.1090571
1.34E-10	19.05949635
1.37E-10	19.02442822
1.46E-10	18.93910087
1.55E-10	18.86520661
1.57E-10	18.83513021
1.63E-10	18.78607155
1.64E-10	18.76605684
1.66E-10	18.74378811
1.73E-10	18.69169271
1.82E-10	18.63148391
1.84E-10	18.60615743
1.99E-10	18.51856754
2.02E-10	18.48927227
2.10E-10	18.44206057
2.51E-10	18.25560453
2.51E-10	18.24216576
2.60E-10	18.18589642
2.67E-10	18.13648747
2.76E-10	18.09552413
2.96E-10	17.99369057
3.22E-10	17.90121812
3.36E-10	17.84881634
4.18E-10	17.62501258
4.67E-10	17.50610804
4.73E-10	17.48461165
4.80E-10	17.46010927
5.25E-10	17.3414415
5.25E-10	17.3147467
5.46E-10	17.25658554
5.64E-10	17.21626534
5.71E-10	17.19503909
5.71E-10	17.18579797
6.01E-10	17.12649089
6.31E-10	17.07120016
6.34E-10	17.05479505
6.34E-10	17.04836624
6.39E-10	17.03183809
6.82E-10	16.96065823
6.90E-10	16.94032643
6.93E-10	16.92794959
8.20E-10	16.7460634
8.20E-10	16.73636404
8.20E-10	16.73118652
9.89E-10	16.52499483
9.89E-10	16.52375671

1.02E-09	16.47748962
1.02E-09	16.47359603
1.03E-09	16.46099622
1.16E-09	16.32468818
1.16E-09	16.32179299
1.24E-09	16.22943669
1.29E-09	16.1846267
1.33E-09	16.14321864
1.40E-09	16.0854322
1.42E-09	16.06414902
1.43E-09	16.05346832
1.55E-09	15.94822433
1.59E-09	15.90865476
1.69E-09	15.84343847
1.74E-09	15.80046594
1.74E-09	15.79134475
1.78E-09	15.76582469
1.78E-09	15.75644511
2.25E-09	15.49499169
2.25E-09	15.49047953
2.41E-09	15.41070134
2.48E-09	15.37613151
2.78E-09	15.26033407
3.08E-09	15.14759005
3.11E-09	15.13258278
3.11E-09	15.12058802
3.16E-09	15.09981714
3.22E-09	15.07557964
3.27E-09	15.05267593
3.53E-09	14.95623912
3.89E-09	14.85378366
3.89E-09	14.84295169
3.89E-09	14.83740848
3.89E-09	14.83325056
3.98E-09	14.79980659
4.12E-09	14.75655895
4.53E-09	14.64711834
4.65E-09	14.61118083
4.65E-09	14.60684992
4.79E-09	14.57122292
4.87E-09	14.55004877
5.11E-09	14.48325217
5.54E-09	14.3991763
5.82E-09	14.32691897
5.94E-09	14.29723103
6.24E-09	14.24485547
6.39E-09	14.21696298
6.88E-09	14.13001234
7.21E-09	14.06939833
7.81E-09	13.98240065
8.54E-09	13.87771462
8.57E-09	13.86436121
8.58E-09	13.85906856

9.32E-09	13.75631968
9.45E-09	13.73031534
9.47E-09	13.72333631
9.53E-09	13.71277443
9.74E-09	13.68371469
9.88E-09	13.6656155
9.96E-09	13.65004394
9.98E-09	13.64326081
1.01E-08	13.6269118
1.01E-08	13.62190611
1.07E-08	13.54219323
1.07E-08	13.53813187
1.08E-08	13.52463358
1.11E-08	13.49512281
1.13E-08	13.4718077
1.15E-08	13.450352
1.16E-08	13.43848504
1.16E-08	13.43529397
1.19E-08	13.38849482
1.19E-08	13.38820947
1.19E-08	13.38759645
1.20E-08	13.37949354
1.28E-08	13.30330591
1.30E-08	13.28364417
1.31E-08	13.27105597
1.35E-08	13.22939522
1.39E-08	13.18208206
1.56E-08	13.06697899
1.68E-08	12.97947982
1.75E-08	12.92480307
1.80E-08	12.89243702
1.84E-08	12.86852755
2.07E-08	12.74302874
2.09E-08	12.72774528
2.10E-08	12.72233647
2.10E-08	12.72028864
2.21E-08	12.66128964
2.24E-08	12.64152036
2.37E-08	12.58040072
2.50E-08	12.51961791
2.62E-08	12.46698752
2.62E-08	12.46673737
2.73E-08	12.42052693
2.74E-08	12.41134603
2.75E-08	12.40553796
2.81E-08	12.3709545
2.82E-08	12.36293189
2.89E-08	12.32543718
3.16E-08	12.20984092
3.16E-08	12.20938831
3.28E-08	12.16684947
3.43E-08	12.11841291
3.44E-08	12.11009926

3.44E-08	12.10659074
3.50E-08	12.0767824
3.51E-08	12.07107355
3.60E-08	12.03922651
3.75E-08	11.98952367
4.05E-08	11.90238216
4.30E-08	11.83205327
4.38E-08	11.80768689
4.38E-08	11.80419287
4.41E-08	11.78815916
4.85E-08	11.67486503
5.02E-08	11.637511
5.26E-08	11.58534216
5.50E-08	11.54027946
5.97E-08	11.44357357
5.97E-08	11.44010175
6.06E-08	11.42219162
6.19E-08	11.39116434
6.73E-08	11.3026263
6.79E-08	11.28599666
7.25E-08	11.21025051
7.27E-08	11.19969554
7.27E-08	11.19612521
7.35E-08	11.18208826
7.35E-08	11.18011637
7.39E-08	11.17014626
7.52E-08	11.15120627
7.58E-08	11.14114791
7.97E-08	11.08934247
8.16E-08	11.06392067
8.90E-08	10.96192899
9.10E-08	10.93355265
9.46E-08	10.88498204
9.46E-08	10.88381256
9.87E-08	10.83775834
1.01E-07	10.81583716
1.13E-07	10.68381663
1.17E-07	10.64915841
1.20E-07	10.61196167
1.27E-07	10.54585368
1.28E-07	10.53792679
1.29E-07	10.51843585
1.37E-07	10.45977525
1.40E-07	10.42836951
1.43E-07	10.4042541
1.45E-07	10.39043497
1.46E-07	10.37676063
1.53E-07	10.31929714
1.54E-07	10.31242131
1.55E-07	10.30368798
1.63E-07	10.242039
1.63E-07	10.24005662
1.68E-07	10.20624179

1.71E-07	10.18159308
1.78E-07	10.1296173
1.82E-07	10.10762527
1.87E-07	10.07843468
1.89E-07	10.06275325
1.93E-07	10.03747392
1.93E-07	10.03506518
1.95E-07	10.02079869
2.17E-07	9.911909841
2.24E-07	9.879623019
2.28E-07	9.856304407
2.36E-07	9.821359729
2.51E-07	9.75509868
2.53E-07	9.746898158
2.90E-07	9.586707279
3.10E-07	9.518629441
3.11E-07	9.510529451
3.15E-07	9.496471006
3.22E-07	9.470455178
3.23E-07	9.46302429
3.33E-07	9.433436187
3.81E-07	9.291335382
3.86E-07	9.275031939
4.05E-07	9.218414931
4.20E-07	9.176428418
4.22E-07	9.171687424
4.27E-07	9.153621269
4.36E-07	9.124077147
4.36E-07	9.123907064
4.43E-07	9.097691423
4.61E-07	9.046876205
5.09E-07	8.93954363
5.12E-07	8.931859088
5.15E-07	8.924051435
5.20E-07	8.910122873
5.20E-07	8.908013534
5.20E-07	8.905988433
5.53E-07	8.840449659
5.65E-07	8.816275308
5.76E-07	8.796320097
5.78E-07	8.7902614
6.00E-07	8.748502217
6.31E-07	8.697337097
6.47E-07	8.666364014
6.48E-07	8.663156502
6.73E-07	8.619502007
6.81E-07	8.605177451
6.95E-07	8.579901337
7.07E-07	8.562151015
7.12E-07	8.552958834
7.49E-07	8.498157101
7.61E-07	8.480203162
7.66E-07	8.469895512

7.72E-07	8.458968163
8.14E-07	8.399723864
8.32E-07	8.37391601
8.68E-07	8.325173859
8.73E-07	8.317362495
8.91E-07	8.296146886
8.91E-07	8.295064991
8.95E-07	8.286083216
8.95E-07	8.285361701
9.30E-07	8.240663427
9.38E-07	8.228986883
1.00E-06	8.158720535
1.00E-06	8.157014927
1.01E-06	8.141180399
1.02E-06	8.128509721
1.06E-06	8.083180864
1.07E-06	8.077454416
1.07E-06	8.066247809
1.10E-06	8.041333159
1.11E-06	8.031680887
1.12E-06	8.018451642
1.13E-06	8.005209951
1.17E-06	7.971021146
1.17E-06	7.965151151
1.21E-06	7.924483964
1.22E-06	7.914968153
1.24E-06	7.899202051
1.27E-06	7.866154874
1.27E-06	7.864828298
1.27E-06	7.864420506
1.28E-06	7.856454835
1.30E-06	7.838900318
1.32E-06	7.822090875
1.36E-06	7.788102968
1.38E-06	7.769463217
1.44E-06	7.716329649
1.45E-06	7.706028235
1.46E-06	7.698272405
1.53E-06	7.638654119
1.53E-06	7.634876869
1.65E-06	7.547647193
1.68E-06	7.526779407
1.68E-06	7.523299466
1.75E-06	7.478784664
1.79E-06	7.457629042
1.79E-06	7.453782151
1.96E-06	7.355172785
2.00E-06	7.32981118
2.09E-06	7.276356393
2.26E-06	7.189488465
2.31E-06	7.165763449
2.36E-06	7.135575713
2.41E-06	7.112794652

2.52E-06	7.065173968
2.56E-06	7.046611481
2.62E-06	7.022313889
2.66E-06	7.00355912
2.71E-06	6.97611039
2.73E-06	6.967921453
2.74E-06	6.960026492
2.89E-06	6.905853333
3.11E-06	6.826575805
3.24E-06	6.775822102
3.34E-06	6.741181371
3.35E-06	6.736426365
3.35E-06	6.736247176
3.48E-06	6.687679171
3.69E-06	6.616298765
3.72E-06	6.602327506
4.00E-06	6.516788629
4.28E-06	6.43330539
4.41E-06	6.400230489
4.45E-06	6.389710569
4.66E-06	6.335685537
4.66E-06	6.333648301
4.76E-06	6.307956023
4.88E-06	6.280709853
4.89E-06	6.27868489
5.07E-06	6.238112735
5.09E-06	6.231812849
5.33E-06	6.181882819
5.47E-06	6.155624005
5.51E-06	6.147553951
5.67E-06	6.116914255
5.81E-06	6.087038266
5.93E-06	6.061093226
5.93E-06	6.060791228
6.13E-06	6.022627865
6.23E-06	6.004404118
6.59E-06	5.94149041
6.60E-06	5.938321759
6.68E-06	5.924603343
7.18E-06	5.844787318
7.33E-06	5.819266066
7.67E-06	5.769551687
8.02E-06	5.718224182
8.02E-06	5.716884176
8.17E-06	5.695519582
8.21E-06	5.689449649
8.28E-06	5.680957081
8.85E-06	5.608434209
8.85E-06	5.607956184
9.30E-06	5.550109472
9.43E-06	5.534585252
9.45E-06	5.531152786
1.00E-05	5.468353676

1.04E-05	5.422806599
1.04E-05	5.422613138
1.07E-05	5.391882113
1.09E-05	5.367013403
1.11E-05	5.345771389
1.12E-05	5.338909092
1.13E-05	5.33088833
1.18E-05	5.277681974
1.22E-05	5.239848278
1.22E-05	5.235364543
1.25E-05	5.211308496
1.29E-05	5.175462815
1.33E-05	5.142722671
1.35E-05	5.129106092
1.39E-05	5.092969994
1.43E-05	5.062599101
1.46E-05	5.039136657
1.48E-05	5.025117154
1.51E-05	5.002763572
1.54E-05	4.978839219
1.63E-05	4.915549939
1.64E-05	4.91235938
1.68E-05	4.881469996
1.69E-05	4.871096437
1.72E-05	4.853625741
1.73E-05	4.843206567
1.75E-05	4.831509053
1.82E-05	4.786987861
1.87E-05	4.75786474
1.89E-05	4.748392877
1.90E-05	4.73836068
1.92E-05	4.73055512
2.02E-05	4.669151852
2.04E-05	4.658024031
2.07E-05	4.640522052
2.13E-05	4.609801446
2.26E-05	4.533222313
2.30E-05	4.517444326
2.42E-05	4.457519618
2.43E-05	4.45394487
2.49E-05	4.425761594
2.54E-05	4.402448745
2.83E-05	4.28202208
2.87E-05	4.265393413
2.91E-05	4.253909457
2.91E-05	4.250179105
2.98E-05	4.224498132
3.00E-05	4.215791524
3.03E-05	4.206925016
3.04E-05	4.201261949
3.20E-05	4.145461442
3.27E-05	4.120181569
3.30E-05	4.106503164

3.44E-05	4.056653596
3.53E-05	4.024290863
3.55E-05	4.019764447
3.57E-05	4.01258136
3.73E-05	3.963542277
3.77E-05	3.94768768
3.89E-05	3.914407534
3.90E-05	3.910933203
4.18E-05	3.840228347
4.25E-05	3.820135618
4.25E-05	3.819077443
4.46E-05	3.766030651
4.51E-05	3.750067435
4.72E-05	3.704665397
4.73E-05	3.700478651
4.75E-05	3.696532989
4.94E-05	3.652390226
4.94E-05	3.651324269
5.02E-05	3.633542516
5.11E-05	3.615023765
5.18E-05	3.599310719
5.91E-05	3.452210398
5.94E-05	3.44553884
5.96E-05	3.439574796
6.19E-05	3.397568853
6.29E-05	3.380076856
6.38E-05	3.36390424
6.39E-05	3.360964458
6.41E-05	3.357335317
6.60E-05	3.327221554
6.67E-05	3.313562483
6.72E-05	3.304101285
6.97E-05	3.26341646
7.35E-05	3.204132928
7.41E-05	3.194297005
7.72E-05	3.149159597
7.85E-05	3.131503934
8.40E-05	3.057611854
8.48E-05	3.047718185
9.19E-05	2.956670096
9.19E-05	2.955648231
9.42E-05	2.929287647
9.50E-05	2.918384675
9.60E-05	2.906535708
9.75E-05	2.889073626
9.84E-05	2.878195359
0.000100062	2.860163482
0.000101842	2.841359825
0.000106719	2.788991666
0.000110654	2.748119769
0.000112644	2.728780191
0.000114544	2.707649606
0.000115633	2.697351189

0.000115928	2.693572801
0.000116833	2.682609269
0.000118657	2.665938453
0.000119399	2.657553784
0.000121564	2.635450048
0.000121564	2.634924671
0.000149594	2.403749727
0.000150614	2.395961716
0.000152312	2.383228236
0.000160291	2.324262219
0.000161766	2.310392173
0.000163956	2.296371276
0.000173194	2.234293621
0.000176905	2.208415292
0.000176965	2.207537762
0.000176967	2.206969904
0.000183757	2.164269646
0.00018395	2.16271241
0.000189887	2.126798849
0.000191714	2.115988427
0.000192826	2.108813106
0.000194309	2.098798441
0.000195456	2.091491066
0.000205157	2.0380786
0.000216346	1.980266901
0.000226269	1.928832833
0.000242433	1.851757009
0.000246396	1.83259247
0.000253344	1.801317073
0.000254562	1.795693787
0.000261886	1.765064867
0.000266234	1.747651244
0.00026855	1.737825599
0.000269114	1.735307702
0.000280207	1.690031119
0.000288745	1.658282213
0.000297192	1.626159718
0.000298744	1.61916322
0.000306797	1.588209439
0.000314198	1.563928318
0.000319209	1.54581447
0.000321416	1.538231832
0.000324772	1.526815896
0.000334047	1.495450074
0.000334374	1.494019701
0.000337761	1.481429983
0.00034072	1.470144087
0.000343228	1.462158093
0.00034495	1.456395769
0.000346296	1.451367264
0.00035132	1.436473517
0.00035475	1.423289252
0.000365048	1.389432932

0.000369849	1.372837401
0.000382956	1.334312299
0.000400576	1.28756133
0.00040585	1.274128941
0.000432262	1.203094906
0.000433963	1.197392416
0.000438211	1.185832556
0.000442382	1.173989776
0.00044334	1.171460953
0.000444518	1.167064922
0.000447228	1.160335923
0.000471369	1.103259013
0.000512861	1.008849409
0.000520362	0.991642105
0.000564144	0.902101216
0.000583601	0.865361202
0.000597167	0.839493388
0.000606266	0.823775195
0.000635407	0.771177191
0.000643232	0.758657229
0.000654455	0.740890956
0.000659936	0.730774668
0.000688008	0.683337919
0.000701852	0.659219549
0.000706434	0.651744389
0.000717955	0.63464987
0.000719359	0.63236465
0.00075379	0.582253717
0.000765145	0.566895428
0.000808608	0.506368639
0.000859743	0.437666157
0.000884777	0.40575222
0.00093504	0.34106426
0.000935154	0.34053969
0.0009763	0.291962859
0.001011082	0.254321267
0.001019589	0.245168144
0.001039434	0.222072556
0.001052221	0.208992215
0.001052221	0.208882208
0.001071916	0.187714065
0.001246839	0.021735782
0.001254021	0.015911591
0.001324275	-0.044936976
0.001380779	-0.096674022
0.001399456	-0.110893012
0.001404432	-0.114627293
0.001408821	-0.117955871
0.001426379	-0.134029635
0.00151692	-0.197233221
0.001556453	-0.224900284
0.00155792	-0.226162923
0.001561108	-0.228552785

0.001598825	-0.254732321
0.001668074	-0.300635858
0.001758686	-0.360138704
0.001821027	-0.40098868
0.001822169	-0.401941226
0.001880454	-0.434519127
0.00190472	-0.45096825
0.001912828	-0.456221816
0.001918473	-0.460115251
0.001943333	-0.474131697
0.001952717	-0.479614972
0.001953403	-0.480429279
0.001953878	-0.481141127
0.001966178	-0.48941905
0.001987956	-0.501928329
0.002014399	-0.515501309
0.002042837	-0.530769731
0.002130122	-0.576860581
0.002145376	-0.584064676
0.002147445	-0.585888265
0.002363262	-0.6878327
0.002530213	-0.763682516
0.002645278	-0.810912362
0.002732371	-0.847081008
0.002859128	-0.899364786
0.002906457	-0.9167707
0.00293087	-0.925284919
0.003010442	-0.953402523
0.003183381	-1.013692438
0.003243021	-1.033712066
0.003262876	-1.040413227
0.003335787	-1.064708354
0.003396451	-1.083920818
0.003431066	-1.096280497
0.003454816	-1.103711503
0.003484387	-1.113307055
0.003526625	-1.126844093
0.003663216	-1.168188446
0.00366395	-1.168695137
0.003669961	-1.170867546
0.003898211	-1.234629868
0.003990788	-1.259422433
0.004160728	-1.305175058
0.004332841	-1.349419702
0.004395519	-1.365925694
0.004404502	-1.368149758
0.004610134	-1.421203695
0.004621898	-1.42419344
0.004671564	-1.435389339
0.004688296	-1.439640057
0.004690939	-1.440470626
0.004901937	-1.491506648
0.00491468	-1.494830482

0.004935599	-1.499693225
0.005059333	-1.527464408
0.005235824	-1.564297208
0.005325411	-1.583365561
0.005390119	-1.59520737
0.005669707	-1.647893946
0.00591091	-1.692808871
0.005949392	-1.700011205
0.005975667	-1.70440121
0.006095502	-1.724611435
0.006130434	-1.731086378
0.006321894	-1.766321977
0.006645309	-1.823261163
0.006657238	-1.82577969
0.006817931	-1.851327548
0.006990227	-1.879569884
0.007020839	-1.88503033
0.007368266	-1.937101103
0.007435224	-1.947713375
0.008760477	-2.13706913
0.009078895	-2.176423123
0.010157262	-2.29725916
0.010350676	-2.318004514
0.010450708	-2.327641231
0.010634234	-2.349368991
0.010940128	-2.383335624
0.01165611	-2.450990718
0.012619034	-2.535377684
0.015740656	-2.770119181
0.016409889	-2.814453624
0.016444935	-2.81740273
0.016558149	-2.826762161
0.017095501	-2.862919834
0.018011092	-2.918824848
0.018540365	-2.950030457
0.019275133	-2.99183659
0.019690986	-3.015059615
0.021661183	-3.120442348
0.022480516	-3.1599566
0.025498145	-3.295220231
0.030064596	-3.479370074
0.03611827	-3.683278752
0.038207204	-3.747049877
0.038934032	-3.767106675
0.039315898	-3.777132552
0.042982677	-3.872957065
0.04299351	-3.873366391
0.044053333	-3.900343539
0.044905463	-3.92119424
0.046461915	-3.957407887
0.047666386	-3.985312053
